# *Sergentomyia schwetzi*: Salivary gland transcriptome, proteome and enzymatic activities in two lineages adapted to different blood sources

**DOI:** 10.1371/journal.pone.0230537

**Published:** 2020-03-24

**Authors:** Nikola Polanska, Aygul Ishemgulova, Vera Volfova, Pavel Flegontov, Jan Votypka, Vyacheslav Yurchenko, Petr Volf

**Affiliations:** 1 Department of Parasitology, Faculty of Science, Charles University, Prague, Czech Republic; 2 Life Science Research Centre, Faculty of Science, University of Ostrava, Ostrava, Czech Republic; 3 Biology Centre, Institute of Parasitology, Czech Academy of Sciences, České Budějovice (Budweis), Czech Republic; 4 Martsinovsky Institute of Medical Parasitology, Tropical and Vector Borne Diseases, Sechenov University, Moscow, Russia; Instituto Oswaldo Cruz, BRAZIL

## Abstract

During the blood feeding, sand fly females inject saliva containing immunomodulatory and anti-haemostatic molecules into their vertebrate hosts. The saliva composition is species-specific, likely due to an adaptation to particular haemostatic pathways of their preferred host. Research on sand fly saliva is limited to the representatives of two best-studied genera, *Phlebotomus* and *Lutzomyia*. Although the members of the genus *Sergentomyia* are highly abundant in many areas in the Old World, their role in human disease transmission remains uncertain. Most *Sergentomyia* spp. preferentially attack various species of reptiles, but feeding on warm-blooded vertebrates, including humans and domestic animals, has been repeatedly described, especially for *Sergentomyia schwetzi*, of which salivary gland transcriptome and proteome is analyzed in the current study. Illumina RNA sequencing and *de novo* assembly of the reads and their annotation revealed 17,293 sequences homologous to other arthropods’ proteins. In the sialome, all proteins typical for sand fly saliva were identified–antigen 5-related, lufaxin, yellow-related, PpSP15-like, D7-related, ParSP25-like, and silk proteins, as well as less frequent salivary proteins included 71kDa-like, ParSP80-like, SP16-like, and ParSP17-like proteins. Salivary enzymes include apyrase, hyaluronidase, endonuclease, amylase, lipase A2, adenosine deaminase, pyrophosphatase, 5’nucleotidase, and ribonuclease. Proteomics analysis of salivary glands identified 631 proteins, 81 of which are likely secreted into the saliva. We also compared two *S*. *schwetzi* lineages derived from the same origin. These lineages were adapted for over 40 generations for blood feeding either on mice (S-M) or geckos (S-G), two vertebrate hosts with different haemostatic mechanisms. Altogether, 20 and 40 annotated salivary transcripts were up-regulated in the S-M and S-G lineage, respectively. Proteomic comparison revealed ten salivary proteins more abundant in the lineage S-M, whereas 66 salivary proteins were enriched in the lineage S-G. No difference between lineages was found for apyrase activity; contrarily the hyaluronidase activity was significantly higher in the lineage feeding on mice.

## Introduction

Phlebotomine sand flies (Diptera, Psychodidae) are bloodsucking insects and vectors of viruses, bacteria and protists, causative agents of several important diseases of humans and animals. Out of over 800 described sand fly species, about 100 (all belonging to the two genera: *Phlebotomus* and *Lutzomyia*) are proven or suspected vectors of medical importance [1,2]. Sand flies of the third genus, *Sergentomyia*, are highly abundant in many areas in the Old World, but their vectorial role in human diseases is uncertain. Most of them exhibit a preference to feed on cold-blooded vertebrates (such as reptiles and amphibians), yet feeding on warm-blooded vertebrates, including domestic animals and humans, was also described [3,4]. Traditionally, *Sergentomyia* sand flies were considered as insects with possible vector role in the transmission of reptile trypanosomatid parasites belonging to the genera *Trypanosoma* and *Leishmania* [5–7], but more recently they have been found positive for several human pathogenic viruses, namely Chandipura [8], Toscana [9], and Dashli virus [10], despite that *Sergentomyia* sand flies vectorial capacity is still under consideration.

During the blood feeding process, a female sand fly injects saliva into the host skin. Saliva has anti-haemostatic, immunomodulatory and anti-inflammatory properties, enabling the successful completion of the blood uptake by the sand fly (reviewed in [11]). Sand fly saliva can also facilitate transmission of the *Leishmania* parasites and enhance the *Leishmania* infection in the mammalian host [12,13]. While a number of reports focused on the composition and function of *Phlebotomus* and *Lutzomyia* saliva (reviewed in [11]), only a single study dealt with characterization of *Sergentomyia s*alivary enzymes [14].

The salivary composition of blood-feeding insects differs, likely due to their adaptation to a specific haemostatic pathways of their preferred host [15]. The host’s haemostatic system is a complex physiological process that halts bleeding at the site of injury (reviewed in [16]). The compounds involved in the host haemostasis vary across vertebrates [17]. For example, ADP was shown to activate mammalian platelets, but not thrombocytes of birds and reptiles [18]. Thus, biting insects adapted to feeding on birds or reptiles are expected to have lower level of apyrase activity for successful blood feeding on mammals [15]. This could explain why the triatomine bug *Dipetalogaster maxima*, which prefers to feed on lizards, exhibits very low salivary apyrase activities, compared to other triatomine bugs [19]. Similarly, the saliva of *Culex quinquefasciatus*, the species which appears to be adapted to mammalian hosts only recently, exhibits very low anti-clotting and apyrase activities, and, therefore, is not able to effectively prevent platelet aggregation caused by ADP [15]. In other bloodsucking insects, several studies have focused on comparison of salivary compounds between various insect families (reviewed in [20–22]), but not in respect to host preferences.

Here we present data on the salivary gland transcriptome, proteome and salivary enzyme activities of two *S*. *schwetzi* lineages come from a common origin, that were adapted to blood feed either on mice (S-M) or geckos (S-G), two hosts with a different body temperature and haemostatic mechanisms.

## Materials and methods

### Ethics statement

Animals were maintained and handled in the animal facility of Charles University in Prague in accordance with institutional guidelines and Czech legislation (Act No. 246/1992 and 359/2012 coll. on Protection of Animals against Cruelty in present statutes at large), which includes all relevant European Union and international guidelines for experimental animals. The experiments were approved by the Committee on Ethics of Laboratory Experiments of the Charles University (Prague) and were performed under permission of the Ministry of the Environment of the Czech Republic (number: MSMT-10270/2015-6) and the Certificate of Competency (number: CZ 02451) approved by the Ministry of Agriculture of the Czech Republic. Mice were housed in polypropylene breeding cage and geckos in terrarium. Both cages and terrarium were placed in a room with constant room temperature with a 12-hour-light/12-hour-dark cycle and all animals had free access to food and water. All efforts were made to minimize suffering of experimental animals within the study.

### Sand flies

The colony of *Sergentomyia schwetzi* (Adler, Theodor & Parrot, 1929) was established from a pool of eggs obtained during field work in Ethiopia (Sheraro district, Tigray region) in 2011 and it was maintained under standard conditions as described previously [23]. The females from third generation were fed on two different hosts: BALB/c mice and the common leopard gecko, *Eublepharis macularius* (Blyth, 1854). Consequently, one lineage (S-G) was established from 191 female sand flies that were blood fed on the gecko, whereas the second lineage (S-M) derived from 91 females blood fed on BALB/c mice. Both lineages were maintained under the same conditions (but on different hosts) for over 40 generations (3 years) without any bottle-neck effect and only then used for the experiments described below.

### Sample preparation for illumina sequencing

In the sand fly’s salivary glands the majority of mRNA is transcribed during first two days after emerging from pupae and therefore all transcriptomic studies are using 1–2 days old sand flies [24], from which salivary glands were dissected in sterile Tris buffer (20mM Tris, 150 mM NaCl, pH 7,8) and stored in batches of 60 salivary glands (originated from 40 to 50 individual females) in TRIzol (Thermo Fisher Scientific, Waltham, USA) at -80°C until subsequent processing. Three samples from three following generations, each containing 180 salivary glands, were prepared for both lineages of *S*. *schwetzi*. In one day we dissected 60 to 120 salivary glands (one or two batches) from females emerged on that day or one day before in the same pot (one generation). For another batch we dissected newly emerged females from another pot (second generation). We repeated this procedure to reach required amount of salivary glands. Total RNA was extracted according to the manufacturer’s protocol; its quality was assessed using the Agilent Bioanalyzer 2100 (Agilent Technologies, Santa Clara, USA) and quantified on a NanoPhotometer (Implen GmbH, München, Germany). All six samples (three from S-G and three from S-M lineages) were sequenced at Macrogen (Seoul, Republic of Korea) on the Illumina HiSeq^TM^ 4000 platform (Illumina, San Diego, USA).

### Assembly and annotation of the transcriptome

Raw reads were trimmed for sequencing adapters, ambiguity, quality and length in the CLC genomics workbench with standard settings (software versions 7.0.5 to 8.5.1, Qiagen, Hilden, Germany) as described previously [25,26]. Trimmed reads were stored in FastQ format after which a quality check was performed in FastQC [27].

All raw trimmed reads were *de novo* assembled by Trinity v2.2.0 [28,29] with the following settings:—min_kmer_cov 1;—min_contig_length 200. Additionally, *de novo* assembly was verified by mapping the raw trimmed reads back to the assembled contigs using Bowtie v1.1.2 [30] utility in the Trinity pipeline by running a perl script bowtie_PE_separate_then_join.pl (settings: -aligner bowtie—-p 4—all—best–strata). The assembled contigs were then clustered, compared and filtered by CD-HIT-EST v4.6 [31] with an identity parameter 95%. Contigs, which passed the identity parameter, were screened for open reading frames (ORFs) and the single longest ORF was translated to its amino acid sequence using the TransDecoder v3.0.1 [29].

The protein sequences were annotated by finding their closest homologues in NCBInr protein database (National Center for Biotechnology Information non-redundant database) using BLASTp (basic local alignment search tool) with an e-value cut-off ≤ 10^−5^ [32]. All annotated protein sequences were manually sorted into seven groups according to the BLASTp hit organism: bacteria, fungi, plants, vertebrate, protozoa, other invertebrates, and arthropods. The sequences from the arthropod subset were divided into the following groups: sand flies, mosquitoes, other blood-feeding arthropods, and other (non-blood-feeding) arthropods. Further annotations of specific protein domains and families were performed against the Protein family database (Pfam v31.0) [33], the Protein domain families (ProDom v2006.1) [34], the gene ontology (GO) database [35], and the InterPro (classification of protein families) database using the InterProScan software [36]. The GO hits were annotated using the WEGO v2.0 [37,38]. The putative signal peptide cleavage sites in the proteins were predicted by SignalP v4.1 for all sequences [39], and the protein sub-cellular localization was predicted by TargetP v1.1 for arthropod sequences subset [40]. The theoretical molecular weight (Mw) and isoelectric point (pI) of the annotated proteins was predicted by ExPASy Compute pI/Mw [41]. Finally, the potential O-, N- and C-glycosylation sites of the selected proteins were predicted using the NetOGlyc v4.0 Server [42], NetNGlyc v1.0 Server [43] and NetCGlyc v1.0 Server [44].

### Differential gene expression analysis

The RNA-seq analysis of all six transcriptomes (S-G 1–3, S-M 1–3) was performed in the CLC genomics workbench. The transcripts were statistically compared by an “Empirical analysis of differential gene expression” (EDGE) algorithm with default settings, p-value of ≤ 0.05, and FDR (false discovery rate) correction. The transcripts with salivary annotation were then sorted into four groups: i) transcripts with a fold change lesser than 1.5, ii) transcripts with a fold change greater than 1.5, iii) transcripts with a fold change greater than 1.5 and p-value lesser than 0.05. The fourth group of transcripts contains those, which passed the FDR correction regardless their annotation.

### RT-qPCR gene expression analysis

Six independently pooled samples each containing 15 salivary gland pairs were prepared for each lineage. Salivary glands were dissected as above from 1- to 2-day-old sand fly females and stored in RNA*later* RNA Stabilization Reagent (Qiagen) at -80°C for a maximum of 4 hours. Total RNA was isolated using the High Pure RNA Tissue Kit (Roche, Basel, Switzerland) following the manufacturer’s protocol, but eluting RNA into 35 μl of PCR-clean water. The cDNA was then synthesized with a combination of anchored-oligo (dT)_18_ and random hexamer primers using the Transcriptor First Strand cDNA Synthesis Kit (Roche) according to the manufacturer's instructions.

The RT-qPCR (reverse transcription quantitative polymerase chain reaction) primers sequences are listed in S1 Table. The expression of all transcripts was confirmed by PCR using the EmeraldAmp® GT PCR Master Mix (TaKaRax Bio, Inc., Kusatsu City, Japan) (S1 Table) and cDNA from either the S-G or S-M sand flies. PCR products were sequenced directly. All RT-qPCR reactions were performed with the SYBR Green (Bio-Rad, Hercules, USA) using optimized conditions (S1 Table) on the iQ5 Multicolor Real − Time PCR Detection System (Bio-Rad) in the technical duplicates. Relative gene expression values were quantified according to the 2^-ΔΔCT^ method [45] using actin and G3PDH (glycerol-3-phosphate dehydrogenase) as the reference transcripts and all relative gene expression values were calibrated to mean of C_T_ values measured for each transcript from S-G lineage. The R software [46] was used for data evaluation and visualization.

### Phylogenetic analysis

Selected orthologues sequences were aligned by MAFFT (multiple alignment using fast fourier transform) with L-INS-I method [47]. The phylogenetically informative regions were selected using BMGE (block mapping and gathering with entropy) with BLOSUM30, and entropy threshold of 0.4 to 0.5 [48]. Maximum likelihood phylogenetic trees were built in the IQ-TREE software [49] using the ModelFinder [50] with corrected Akaike information criterion and an ultrafast bootstrap approximation with 1,000 replicates [51]. The trees were constructed using various protein substitution models: WAG (for D7-related and PpSP15-like proteins), WAG + I + G4 (for antigen 5-related proteins), WAG + F + I + G4 (for lufaxins and YRPs), WAG + F + G4 (for hyaluronidases), LG + F + R4 (for amylases), LG + F + I + G4 (for apyrases). Phylogenetic trees and the aligned sequences were edited and visualized in FigTree v1.4.3 (http://tree.bio.ed.ac.uk/) and Jalview 2 [52], respectively. Nodes supports in phylogenetic trees were indicated by the bootstrap values, while values higher or equal to 50% were displayed.

### Proteomic analysis

In order to perform the proteomic analysis, 15 salivary glands from 5- to 7-day-old sand fly females were dissected and stored in 15 μl of 100 mM TAE buffer (Tris Acetate EDTA) with 1% SDC (sodium deoxycholate) at 4°C. The dissection was performed between 5^th^ and 7^th^ day after emerging from pupae, as the full protein content in salivary glands is reached only after 4^th^ day [24]. Three salivary gland samples from each lineage were analyzed on mass spectrometry in the OMICS Proteomics laboratory at BIOCEV (Vestec, Czech Republic), each of them in three technical replicates. All the data were analyzed and quantified with the MaxQuant software (v1.5.3.8) [53] with FDR set to 1% for both proteins and peptides and specified minimum length set to seven amino acids. Quantification was performed with the label-free algorithms described previously [53]. The data analysis was performed using Perseus v1.5.2.4 software [54]. Subsequently, the final data were filtered according to their quality and LFQ (log 2-transformed normalized label-free quantification) intensity (threshold 20). The significance of the protein enrichment was statistically evaluated by Student’s T-test. The proteins with salivary annotation were divided into three groups according to difference of LFQ intensity (S-G vs. S-M samples) and Student’s T-test q-value: i) proteins with difference of LFQ intensity lesser than 0.6, ii) proteins with difference of LFQ intensity greater than 0.6, and iii) proteins with difference of LFQ intensity greater than 0.6 with Student’s T-test q-value lesser than 0.05.

For the proteome analysis by the SDS PAGE (sodium dodecyl sulfate–polyacrylamide gel electrophoresis), the salivary glands from 5- to 7-day-old *S*. *schwetzi* females, of both S-G and S-M lineages, were dissected into Tris buffer and stored (-20°C) until used. The samples, with an equivalent protein concentration (20 μg per well), were incubated (95°C, 5 min, sample buffer with 2-mercaptoethanol) and electrophoretically separated at 4°C using two SDS PAGE maxi gels on OmniPAGE Maxi Plus (Cleaver Scientific, Rugby, UK) with 10% and 15% acrylamide, respectively. Gels were stained for total proteins with Coomassie Briliant Blue R-250 (SERVA Electrophoresis GmbH, Heidelberg, Germany). Raw gels are displayed on S1 Fig.

### Sample preparation for measuring enzymatic activities

For the enzymatic activity assays, the newly emerged females of both lineages were separated (to ensure age standardization) and maintained at 26°C on 50% sucrose for 7 days. Dissected salivary glands were transferred to Eppendorf tubes containing either 0.02M TBS (tris-buffered saline), pH 7.6 with 0.005% Triton X-100 for apyrase, or 0.01M PBS, pH 7.2 for the hyaluronidase assays. Samples were stored at -80°C in batches of 10 salivary gland pairs per 20 μl of buffer until used. Salivary gland homogenates (SGHs) were obtained by the disruption of tissue with a plastic pestle after one freeze-thaw cycle in liquid nitrogen. The resulted homogenate was diluted in an appropriate assay buffer to a working concentration and analyzed immediately. The protein concentrations of the SGHs were measured by the Qubit Fluorometer (Thermo Fisher Scientific) following the manufacturer’s instructions.

### Apyrase assay

The apyrase activity was measured using a colorimetric microassay based on the Fiske and Subbarow method [55] with slight modifications [56] on the Infinite M 200 Fluorometer (Tecan, Männedorf, Switzerland) at 665 nm. The concentration of the Pi was calculated from a standard curve with potassium dihydrogen phosphate. One unit of enzyme activity was defined as the amount of enzyme that releases one micromole of orthophosphate per minute from the nucleotide substrate at the specified assay conditions.

### Hyaluronidase assays

The zymographic analysis of hyaluronidase activities were visualized by SDS PAGE electrophoresis in 10% polyacrylamide gels (0.75 mm thick) with copolymerized 0.002% HA (hyaluronic acid) as was described previously [14] with the modification in sample loading– 0.5 of a gland pair was loaded per lane. Raw gels are displayed on S1 Fig.

The hyaluronidase activities were quantified using a method of Frost and Stern [57] with previously described modifications [58]. Briefly: biotinylated HA (bHA) was covalently bound onto Covalink NH microtiter plates (NUNC) at the final concentration of 2 μg of bHA per well, coated for 45 min with 1% BSA in PBS and equilibrated with 100 μl of an appropriate assay buffer: 0.1M citrate-phosphate buffer (pH 4.0–7.0), and 0.1M sodium phosphate buffer (pH 7.0–8.0), all containing 0.1M NaCl and 0.1% Triton X-100. SGH samples were loaded in triplicates at a final concentration of 0.25 of a salivary gland pair per well, incubated for 45 min at 37°C and the reaction was stopped by 6M guanidine (200 μl/well). Avidin-peroxidase (Sigma-Aldrich) was used at a final concentration of 0.2μg/well for 30 min and the color reaction was developed in a substrate buffer with o-phenylenediamine for 15 min in the dark. The plates were read at 492 nm. Bovine testicular hyaluronidase (Sigma-Aldrich) serially diluted in the assay buffer (pH 4.5) served as a standard and wells without bHA or without SGH were used as negative controls. Raw data were evaluated by the Measurement Parameters Editor Magelan 6 (Tecan). The measured activity was expressed as relative turbidity reducing units (rTRU) per pair of salivary glands.

## Results

### Illumina sequencing and read assembly

**Fig 1:**
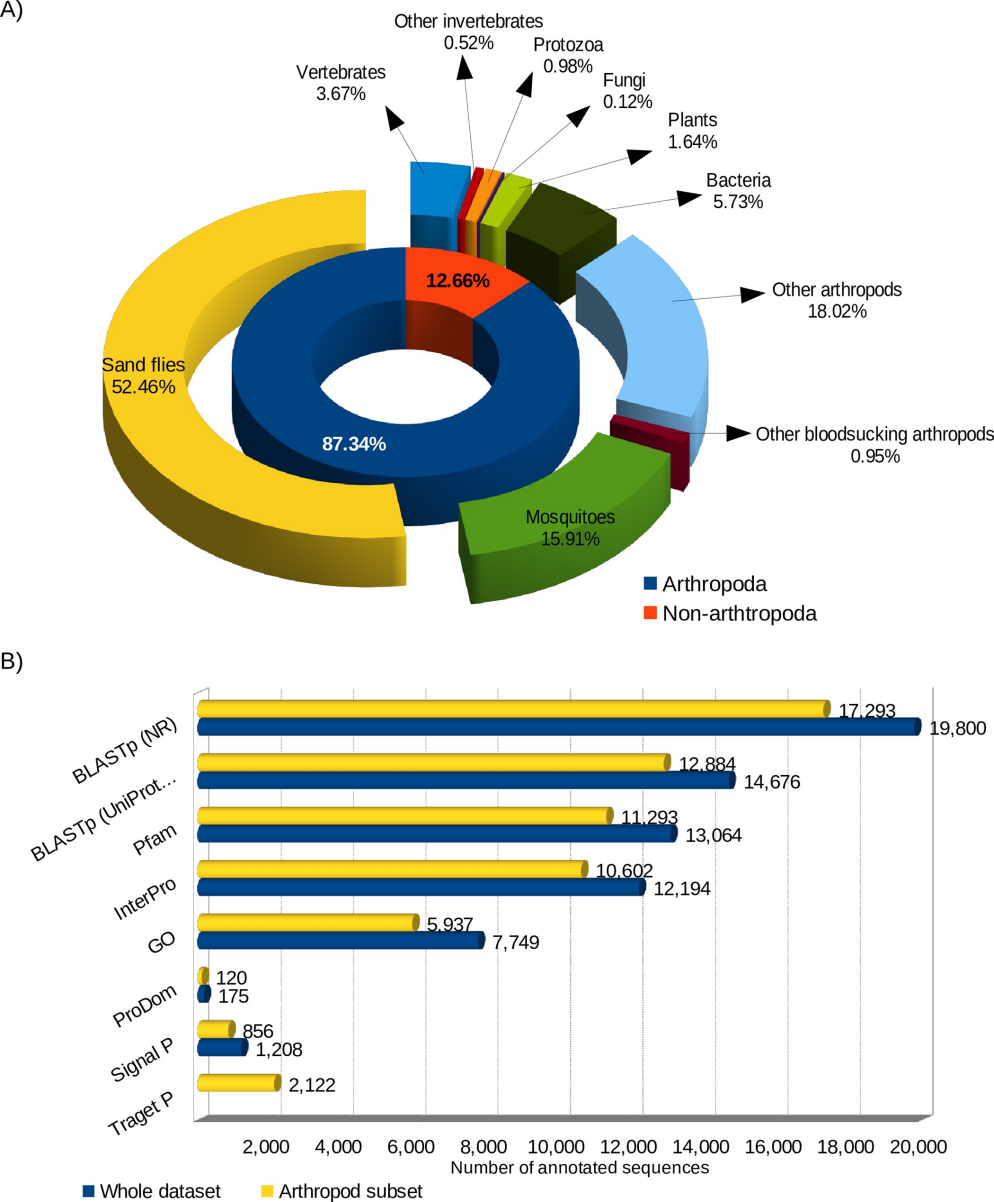
*S*. *schwetzi* salivary gland transcriptome annotation. (A) A chart with sequence annotation groups. Inner part represents distribution of sequences among arthropods (87% of all annotated sequences) and non-arthropods (13%). Outer part represents dividing sequences to 10 groups–sand flies (52.4%, yellow), mosquitoes (16%, green), other blood-sucking arthropods (1%, maroon), other arthropods (18%, azure), bacteria (5.7%, dark green), plants (1.6%, lime), fungi (0.1%, purple), protozoa (1%, orange), other invertebrates (0.5%, red), and vertebrates (3.7%, blue). (B) Results of sequences annotation using different sequence databases. Numbers at the end of each bar indicate number of sequences with hit obtained using each database. Sequences annotated from whole transcriptome sequence dataset are shown in blue and sequences annotated from arthropod sequences subset in yellow, TargetP search was performed only for arthropod sequences subset.

Total of 339,133,323 trimmed reads generated from six libraries of *S*. *schwetzi* salivary glands (S-M 1-3, S-G 1-3) were subsequently assembled into 88,676 contigs (N50 length 1,104 bp; median contig length 329 bp; average contig length 644 bp; total assembled bases 57,076,819). Out of these, 53,976,836 (16% of all reads) reads were matched back to assembled contigs in proper pairs and 61,570 contigs were obtained after clustering similar sequences (with similarity threshold 95%). These were translated into 26,253 protein sequences. Among them, 5,927 ORFs were complete (from Met to stop codon), 9,970 ORFs were partial and 10,356 were identified as internal sequences. All protein sequences were annotated using their closest homologues by searching the NCBInr with 19,800 matched sequences and UniProt/SwissProt database with 14,676 matched sequences. The BLASTp against NCBInr results divided the final dataset into 7 groups: bacteria (1,134 sequences), plants (325 sequences), fungi (24 sequences), protozoans (194 sequences), vertebrates (727 sequences), invertebrates other than arthropods (103 sequences), and arthropods (17,293 sequences) (Fig 1A). In addition, 13,064; 175; and 12,194 sequences were matched in the Pfam, ProDom, and InterPro databases, respectively (Fig 1B), and 7,749 sequences obtained GO terms (S2 Fig). Putative signal peptide cleavage sites were predicted for 1,208 sequences, from which 474 had complete ORF.

Focusing on arthropod sequences only, four main groups were identified: sand flies (10,389 sequences), mosquitoes (3,151 sequences), other bloodsucking arthropods (189 sequences), and other arthropods (3,568 sequences) (Fig 1A). From the arthropods subset, the 4,714; 6,389, and 6,181 ORFs were identified as complete, partial, and internal ORFs, respectively. Within these protein sequences, 12,884; 11,293; 10,602; and 120 hits were matched with UniProt/SwissProt, Pfam, InterPro, and ProDom annotations, respectively. Total of 5,937 hits with various function were annotated with GO (S2 Fig), and 856 and 2,122 proteins were predicted to possess signal or targeting peptides (Fig 1B).

### Identification of salivary protein families

In the salivary gland transcriptome of *S*. *schwetzi*, we found the full-length and/or partial sequences of main members of sand flies’ salivary protein families, namely lufaxin, antigen 5-related proteins, yellow-related proteins, small odorant-binding proteins (PpSP15-like proteins), large odorant-binding proteins (D7-related proteins), silk proteins (PpSP32-like proteins), and ParSP25-like proteins. Furthermore, we documented the expression of the following sand fly salivary enzymes: apyrase, hyaluronidase, endonuclease, amylase, phospholipase A2, adenosine deaminase, pyrophosphatase, 5’nucleotidase and ribonuclease, and proteins: 71kDa-like protein, C-type lectin, ParSP80-like, SP16-like and ParSP17-like proteins and peptide homologous to Lol38.8. These data for proteins with complete ORFs are summarized in Table 1; the partial sequences of the detected salivary proteins are listed in S2 Table along with their annotations.

**Table 1:** Main salivary protein families identified in the transcriptome of *S*. *schwetzi*.

Protein family	Protein name	Mw [kDa]	pI	Best BLASTp match to NCBInr database				GenBank protein accession number
				Species of the best match	Accession number	E-value	Seq. identity [%]	
**Antigen 5-related (Ag5r)**	SschwAg5r1	29.2	8.7	*P*. *argentipes*	ABA12137	9E-118	62	QHO60649
	SschwAg5r2	30.2	9.0	*P*. *duboscqi*	ABI20191	2E-106	57	QHO60650
**Lufaxin (Luf)**	SschwLuf1	31	8.3	*P*. *papatasi*	AGE83098	3E-36	35	QHO60656
	SschwLuf2	35.3	8.7	*L*. *olmeca*	ANW11471	2E-41	32	QHO60657
	SschwLuf3	35.3	8.4	*L*. *olmeca*	ANW11471	2E-41	32	QHO60658
	SschwLuf4	37.2	5.9	*L*. *ayacuchensis*	BAM69113	5E-55	45	QHO60659
	SschwLuf5	37.4	7.9	*P*. *ariasi*	AAX55751	1E-70	45	QHO60660
**Large odorant-binding proteins (D7-related)**	SschwD7_1	26.4	9.1	*P*. *orientalis*	AGT96467	3E-44	37	QHO60662
	SschwD7_2	28.3	8.5	*P*. *perniciosus*	ABA43058	9E-43	35	QHO60663
**Small odorant-binding proteins (PpSP15-like)**	SschwSP15_1	14.8	7.7	*L*. *neivai*	JAV08238	1E-22	38	QHO60683
	SschwSP15_2	31.7	9.3	*L*. *ayacuchensis*	BAM69139	7E-24	38	QHO60684
	SschwSP15_3	14.5	9.0	*P*. *ariasi*	AAX55748	2E-25	39	QHO60685
	SschwSP15_4	15.0	9.3	*P*. *ariasi*	AAX55748	2E-21	35	QHO60686
**Yellow-related proteins (YRPs)**	SschwYRP1	40.9	5.0	*P*. *orientalis*	AGT96428	6E-100	44	QHO60691
	SschwYRP2	39.6	6.0	*P*. *ariasi*	AAX56360	1E-90	41	QHO60692
	SschwYRP3	43.8	8.4	*P*. *argentipes*	ABA12136	3E-79	40	QHO60693
**Apyrase (Apy)**	SschwApy1	35.3	8.4	*L*. *neivai*	JAV08627	4E-137	60	QHO60713
	SschwApy2	35.5	8.7	*L*. *ayacuchensis*	BAM69108	2E-105	47	QHO60714
	SschwApy3	35.9	9.0	*P*. *orientalis*	AGT96431	1E-99	47	QHO60715
**Hyaluronidase (Hya)**	SschwHya1	38.9	8.5	*L*. *longipalpis*	AAD32195	2E-133	56	QHO60718
**5’-nucleotidase (s5nuc)**	Sschw5nuc1	59.9	8.9	*L*. *neivai*	JAV08429	0	57	QHO60721
**Adenosine deaminase (sADA)**	SschwADA1	57.6	5.1	*P*. *perniciosus*	ALL27025	0	69	QHO60732
**Amylase (sAmy)**	SschwAmy1	53.9	6.2	*P*. *papatasi*	AGE83100	0	74	QHO60734
	SschwAmy3	52.7	6.2	*M*. *domestica*	XP_005185446	0	66	QHO60735
	SschwAmy2	54.1	5.3	*P*. *papatasi*	AGE83100	0	59	QHO60736
**Endonuclease (sEnuc)**	SschwEnuc1	52.3	8.7	*L*. *longipalpis*	AAS16916	2E-16	38	QHO60737
**Phospholipase A2 (sPLA2)**	SschwPLA2_1	42.1	8.2	*L*. *neivai*	JAV08461	0	82	QHO60752
**Pyrophosphatase (sPP)**	SschwPP1	47.1	7.4	*P*. *perniciosus*	ALL27023	0	75	QHO60755
**71kDa-like**	Sschw71kDa1	70.9	5.4	*L*. *longipalpis*	AAS16911	0	85	QHO60759
**C-type lectin**	SschwCTL1	19.1	5.0	*L*. *ayacuchensis*	BAM69191	2E-12	33	QHO60772
	SschwCTL2	15.9	5.5	*L*. *neivai*	JAV08591	8E-25	33	QHO60773
**SP16-like**	SschwSP16	17.6	5.6	*P*. *orientalis*	AGT96450	9E-17	34	QHO60782
**ParSP60-like**	SschwSP60-like	15.2	3.9	*P*. *argentipes*	ABA12152	6E-6	38	QHO60783
**ParSP80-like**	SschwSP80	16.2	5.5	*L*. *longipalpis*	AAS16917	2E-99	82	QHO60784
**MBF2-like**	SschwMBF2	15.3	5.8	*P*. *duboscqi*	ABI20163	5E-58	63	QHO60785
**Secreted peptide 1**	SschwPeptide1	7.7	5.0	*L*. *neivai*	JAV08913	8E-27	52	QHO60786
**Secreted peptide 2**	SschwPeptide2	7.8	4.8	*L*. *neivai*	JAV08913	1E-22	45	QHO60787

Complete ORFs of *S*. *schwetzi* annotated as main salivary protein and enzyme families. Protein family name, *S*. *schwetzi* protein name, putative matured protein features (Mw–molecular weight, pI–isoelectric point), BLASTp match to NCBInr protein database and NCBI GenBank protein accession numbers are listed.

### Antigen 5-related proteins

The antigen 5-related proteins (Ag5r) are proteins found in various insect species. In the analyzed transcriptomes, we identified two complete and five partial sequences of Ag5r proteins. Only the complete sequences were used in further analyses. Data on the homologues of *S*. *schwetzi* Ag5r proteins (SschwAg5r) are presented in Table 1 and S2 Table.

Both full-length proteins, SschwAg5r1 and SschwAg5r2 (sequence identity 70.5%), are 290 aa (amino acid) long with predicted Mw (without signal peptides) of 29.2 kDa and 30.2 kDa, respectively. In the sequence of SschwAg5r1, there were no N-glycosylation but 11 putative O-glycosylated sites, while in the sequence of SschwAg5r2, 2 and 5 putative N-glycosylation and O-glycosylation sites were predicted, respectively. The sequence identity of SschwAg5r with other sand fly Ag5r was between 67.6 and 60.2%. All Ag5r proteins share a motif of 14 Cys residues CX_4_CX_9-13_CX_9-10_CX_61–63_CX_6_CX_5_CX_70–71_CX_17-18_CX_2_CX_15_CX_2_CX_4_CX_7_C [59]. This pattern was slightly modified in the sequences of SschwAg5r1 and SschwAg5r2 (S3 Fig).

**Fig 2:**
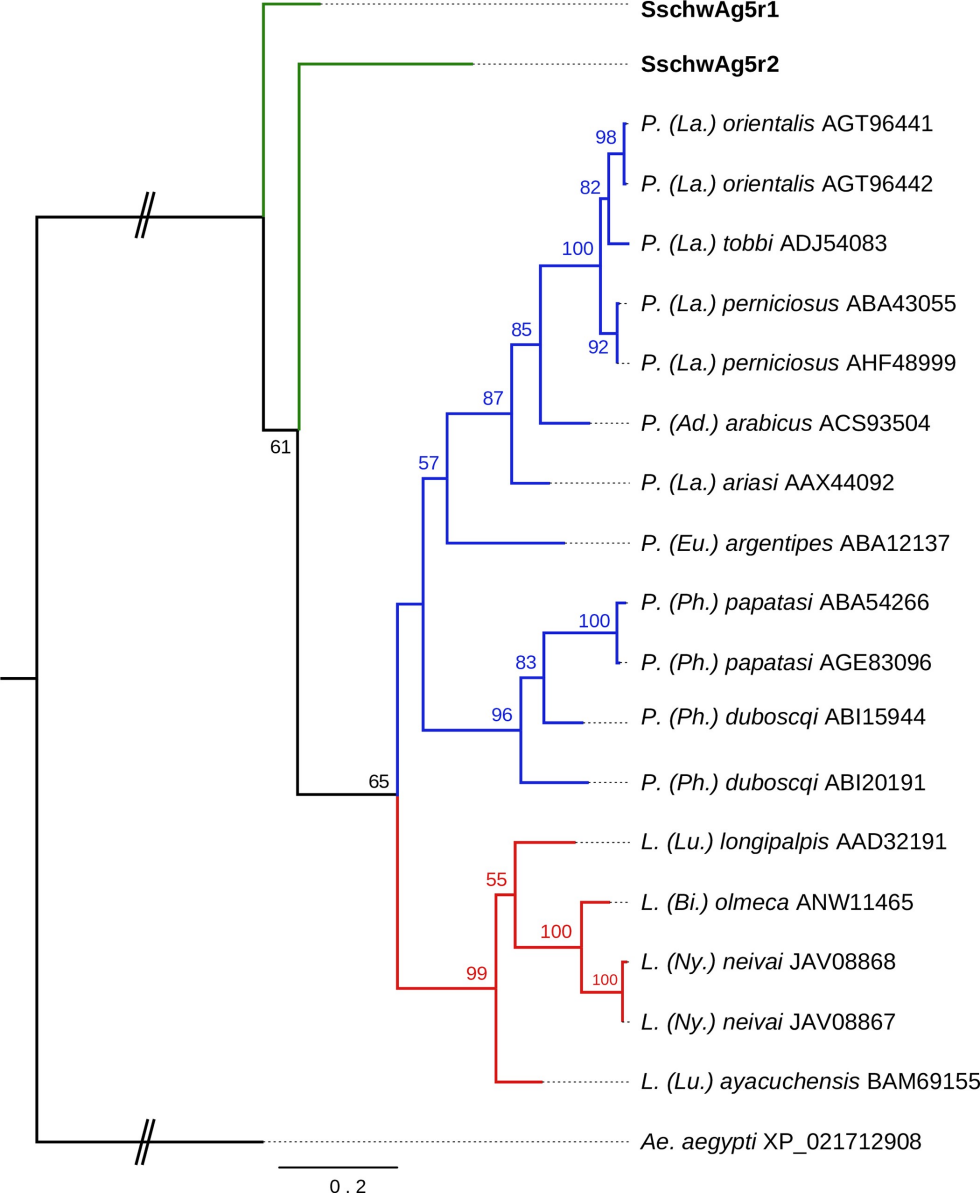
Phylogenetic comparison of antigen 5-related proteins. The phylogenetic tree with 20 sequences was based on MAFFT alignment with BMGE (threshold 0.4) containing 240 aa sites, 124 parsimony-informative sites, 49 singleton sites and 67 constant sites. The node values represent the percentage of bootstrap support for each branch (values equal or higher than 50% are shown). The position of sand flies of *Lutzomyia* genus is marked by red branches color, the sand flies of *Phlebotomus* genus are marked with blue branches color. The *S*. *schwetzi* proteins are marked by green branches color. For rooting the tree, the *Aedes aegypti* (XP_021712908) sequence was used (branch in black). The sand flies’ subgenera are marked by shortcut in parentheses: *Ad*. - *Adlerius*, *La*. - *Larroussius*, *Eu*. - *Euphlebotomus*, *Ph*. - *Phlebotomus*, *Pa*.–*Paraphlebotomus*, *Lu*. - *Lutzomyia*, *Bi*. - *Bichromomyia*, *Ny*. - *Nyssomyia*.

The phylogenetic analysis showed that SschwAg5r1 and SachwAg5r2 are sister to all sequences available from other sand fly species (Fig 2).

### Lufaxin

Lufaxin proteins are sand flies’ specific salivary anticoagulants (inhibitors of factor Xa). Five full-length sequences and one partial sequence of lufaxin homologues were identified in *S*. *schwetzi* transcriptome. The BLASTp hits and more annotations for each SschwLuf sequences are listed in Table 1 and S2 Table.

The amino acid sequences of SschwLuf are between 296 and 337 aa. The putative signal peptide cleavage site was predicted for all SschwLuf, except the SschwLuf5. The predicted molecular weight of SschwLuf varies between 31 kDa and 37.4 kDa and the putative O-glycosylation sites were detected for SschwLuf1 (5 sites), SschwLuf2 (5 sites), SschwLuf3 (6 sites), SschwLuf4 (1 site). The putative N-glycosylation sites were predicted only for SschwLuf4 (2 sites). Six cysteines typical for other sand fly lufaxins consensus sequence CX_21-29_CX_9_CX_22-35_CX_118-125_CX_6_C [60] were conserved across all SschwLuf sequences.

**Fig 3:**
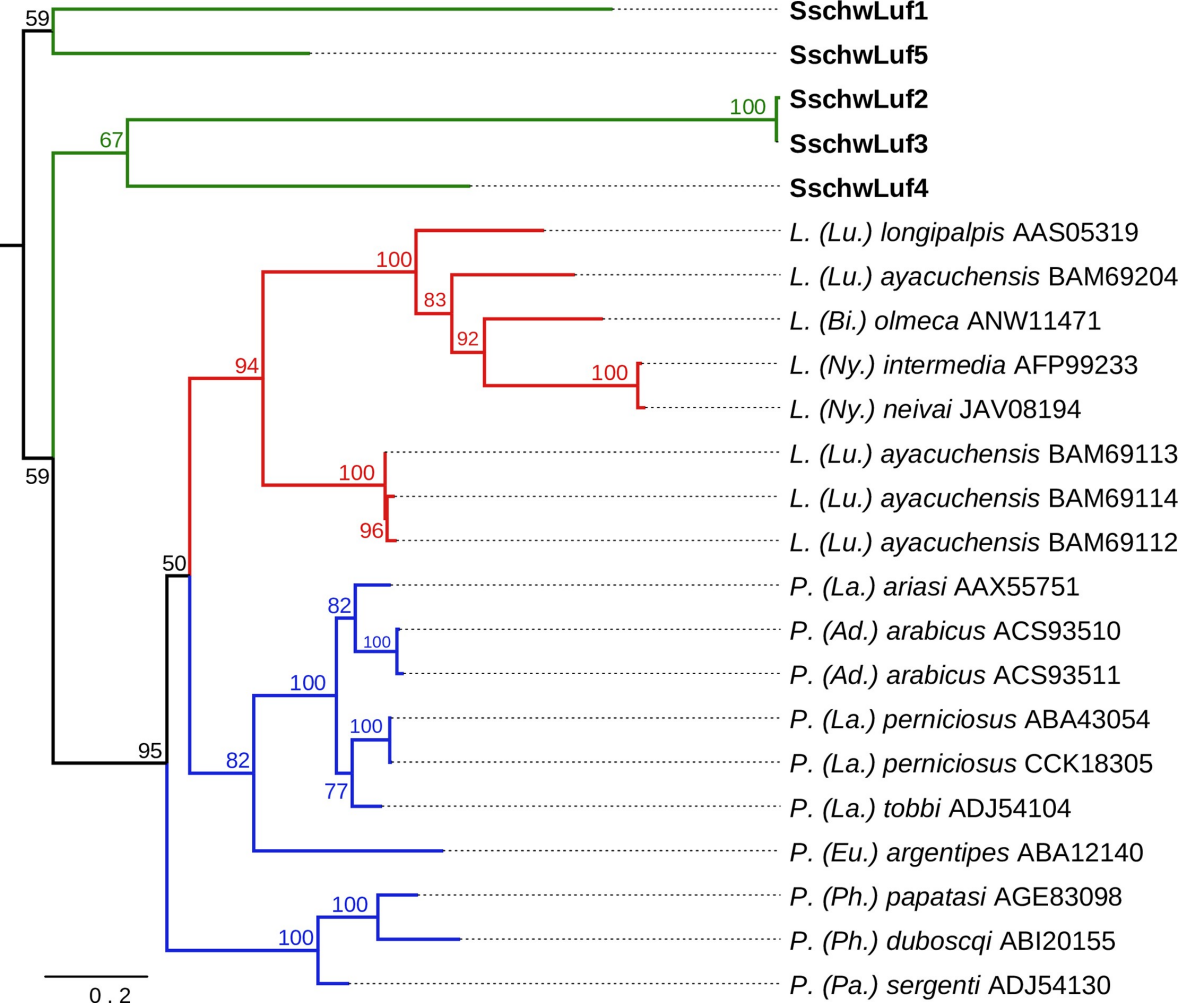
Phylogenetic comparison of lufaxin proteins. The phylogenetic tree with 23 sequences was based on MAFFT alignment with BMGE (threshold 0.5) containing 259 aa sites, 213 parsimony-informative sites, 18 singleton sites and 28 constant sites. The tree is unrooted. For more detail see Fig 2 legend.

The sequence identity between SschwLuf proteins ranged between 30% and 37.6%, except the SschwLuf2 and SschwLuf3, which shared 97.7% sequence identity. Sequence identity with other known sand fly lufaxins varied from 30.3% to 46.8%. The cysteine motifs and putative glycosylations are shown in the S4 Fig. The phylogenetic analysis clustered SschwLuf sequences as two separate groups out from both Old World (OW) and New World (NW) sand flies. Further SschwLuf1 and SschwLuf5 were clustering as one group and SschwLuf2, SschwLuf3 and SschwLuf4 were generating the other group (Fig 3).

### Odorant-binding proteins (OBPs)

#### Large odorant-binding proteins (D7-related)

Large odorant-binding proteins (D7-related) were represented in *S*. *schwetzi* transcriptome by two full-length transcripts, SschwD7_1 and SschwD7_2, and 19 partial transcripts. The further annotations of all SschwD7 sequences are listed in Tables 1 and S1.

Both SschwD7 protein sequences possessed putative signal peptide cleavage sites. The amino acid sequence lengths were 242 aa for SschwD7_1 and 263 aa for SschwD7_2 and the predicted Mw of matured proteins were 26.4 kDa and 28.3 kDa, respectively. Both SschwD7 proteins contained one putative N-glycosylation site and SschwD7_2 had one putative O-glycosylation site (S5 Fig). The other sand flies D7-related proteins contain 10 conserved Cys residues in the pattern CX_25-27_CX_3_CX_44-46_CX_49-50_CX_6-12_CX_3_CX_30-32_CX_9_CX_8_C [59]. The Cys distribution pattern in SschwD7 was slightly modified (S5 Fig).

**Fig 4:**
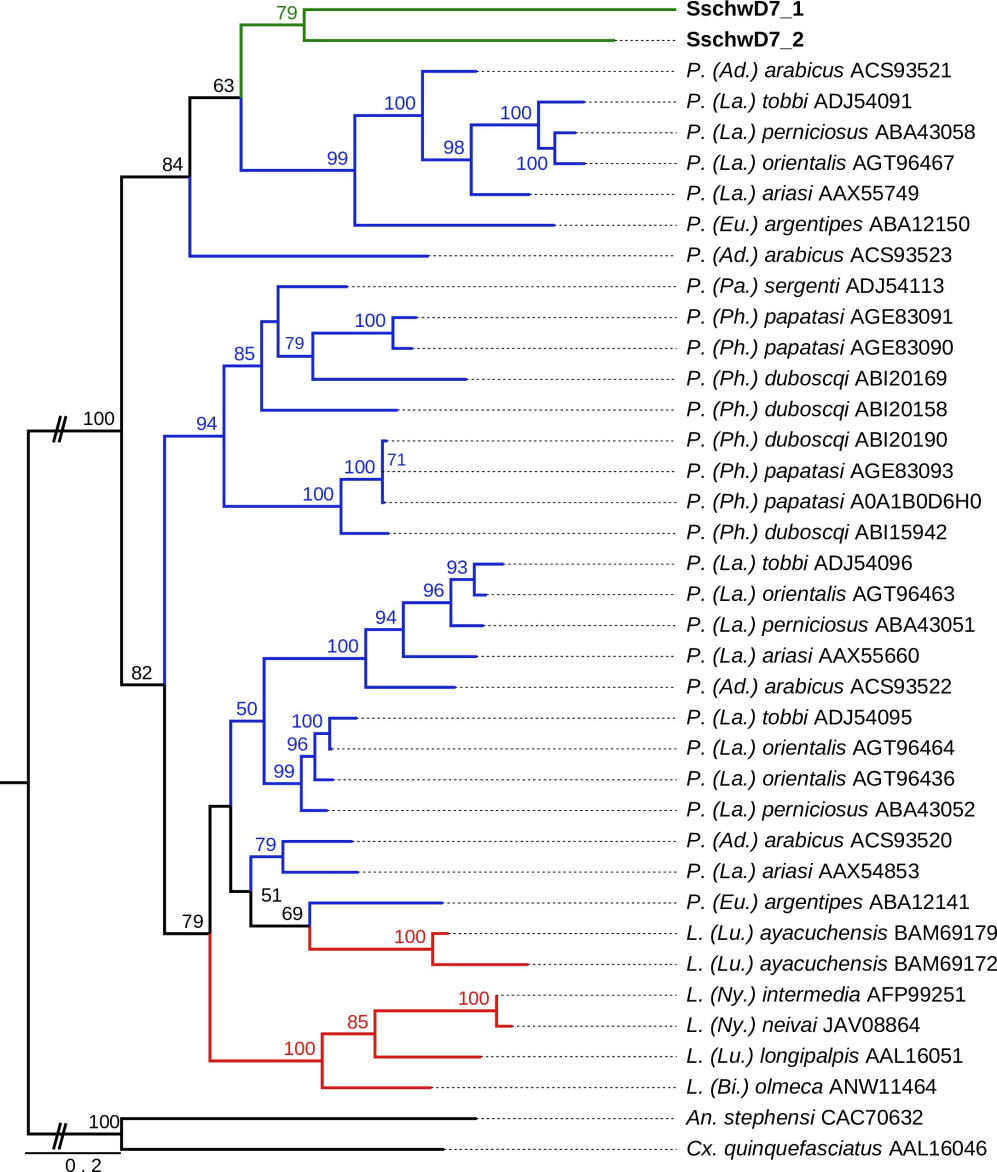
Phylogenetic comparison of large odorant-binding proteins (D7-related). The phylogenetic tree with 38 sequences was based on MAFFT alignment with BMGE (threshold 0.5) containing 213 aa sites, 185 parsimony-informative sites, 19 singleton sites and 9 constant sites. For rooting the tree, the *Anopheles stephensi* (CAC70632) and *Culex quinquefasciatus* (AAL16046) sequences were used (branches in black). For more detail see Fig 2 legend.

The sequence identity between the two SschwD7 was 30.2% and the identity with other sand fly D7-related proteins was between 24.8% and 37.9%. The SschwD7 proteins cluster with other *Phlebotomus* (*Adlerius*, *Larroussius*, and *Euphlebotomus*) proteins; however, the phylogenetic analysis did not give any significant support for closer relationship with these subgenera (Fig 4).

#### Small odorant-binding proteins (PpSP15-like)

Small odorant-binding proteins (PpSP15-like) were detected in *S*. *schwetzi* transcriptome and presented by four complete sequences (SschwSP15_1 –SschwSP15_4) and four partial sequences. The aa length of all sequences, putative Mw and pI for complete SschwSP15 sequences and other annotations are listed in Table 1 and S2 Table.

All full-length sequences contained putative signal peptide cleavage site. Twelve putative O-glycosylation sites were predicted for SschwSP15_2 and one site in the SschwSP15_3 sequence. No putative N- or C-glycosylation sites were detected.

The PpSP15-like proteins also contain 6 Cys residues in the CX_10_CX_3_CX_46_CX_15_CX_8_C pattern [59]. Changes in the SschwSP15_1, SschwSP15_2, SschwSP15_3, and SschwSP15_4 are depicted in the S6 Fig.

The sequence identity among SschwSP15 proteins was relatively high between SschwSP15_3 and SschwSP15_4 (87%) but relatively low between SschwSP15_1 and SschwSP15_2 (43.8%), SschwSP15_1 and SschwSP15_3 (43%) and SschwSP15_1 versus SschwSP15_4 (42.2%). The lowest sequence identity were predicted between SschwSP15_2 and SschwSP15_3 (32.8%) and SschwSP15_2 versus SschwSP15_4 (32.3%). In comparison with other sand fly PpSP15-like proteins the SschwSP15 proteins reached sequence identities between 23% and 40.7%.

**Fig 5:**
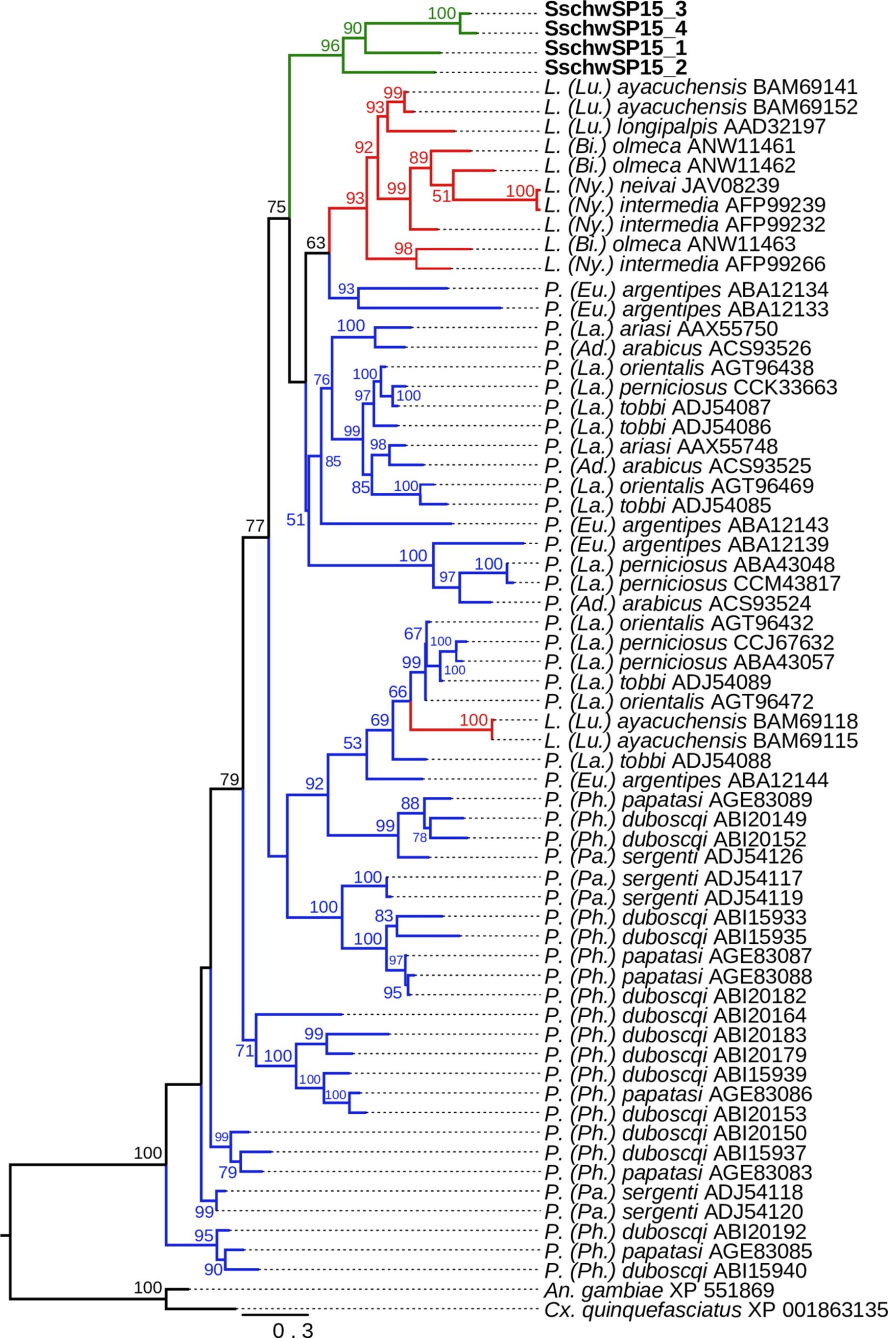
Phylogenetic comparison of small odorant-binding proteins (PpSP15-like). The phylogenetic tree with 67 sequences was based on MAFFT alignment with BMGE (threshold 0.5) containing 213 aa sites, 87 parsimony-informative sites, 1 singleton sites and 6 constant sites. For rooting the tree, the *Anopheles gambiae* (XP_551869) and *Culex quinquefasciatus* (XP_001863135) sequences were used (branches in black). For more detail see Fig 2 legend.

Phylogenetic analysis of four SschwSP15 proteins and other sand fly PpSP15-like proteins revealed a high diversity among this protein family. *S*. *schwetzi* PpSP15-like proteins constitute a sister clade to *Lutzomyia* and part of *Phlebotomus* sequences (Fig 5).

### Yellow-related proteins (YRPs)

Three full-length transcripts and 19 partial transcripts were identified as homologous to other known sand fly YRPs. The description of SschwYRP sequences and their annotation are shown in Table 1 and S2 Table.

The protein sequence length was 387 aa for SschwYRP1, 370 aa for SschwYRP2 and 404 aa SschwYRP3. The predicted Mw of secreted SschwYRP1 was 40.9 kDa and 3 putative N-glycosylation sites were predicted. Mw of matured SschwYRP2 was lower (39.6 kDa) than other YRPs homologues, and no glycosylation site was presented in its sequence. Third protein, SschwYRP3, possessed 2 putative O-glycosylation sites and predicted Mw of 43.8 kDa (secreted variant). All sand fly YRPs sequences share similar Cys motif (CX_67-72_CX_122-123_CX_71-76_C modified according to [60]). Interestingly this sequence design was modified for all three SschwYRPs–SschwYRP1: CX_64_CX_120_CX_69_C; SschwYRP2: CX_57_CX_114_CX_73_C; SschwYRP3: CX_66_CX_138_CX_73_C (S7 Fig). The MRJP (Major royal jelly protein) domain, specific for insect YRPs, was present in all SschwYRPs. Further, the amino acid residues, responsible for binding biogenic amines, were highly conserved only for SschwYRP1. The amine-binding residues in other two SschwYRPs were more variable as it is visible in alignment in S7 Fig.

The sequence identity between SschwYRP1 and SschwYRP2 and between SschwYRP1 and SschwYRP3 was 39.4% and 41.4%, respectively, while the identity between SschwYRP2 and SschwYRP3 was 42.9%. Comparing with the other sand fly YRPs, SschwYRPs shared between 32.7% and 47.1% of the sequence.

**Fig 6:**
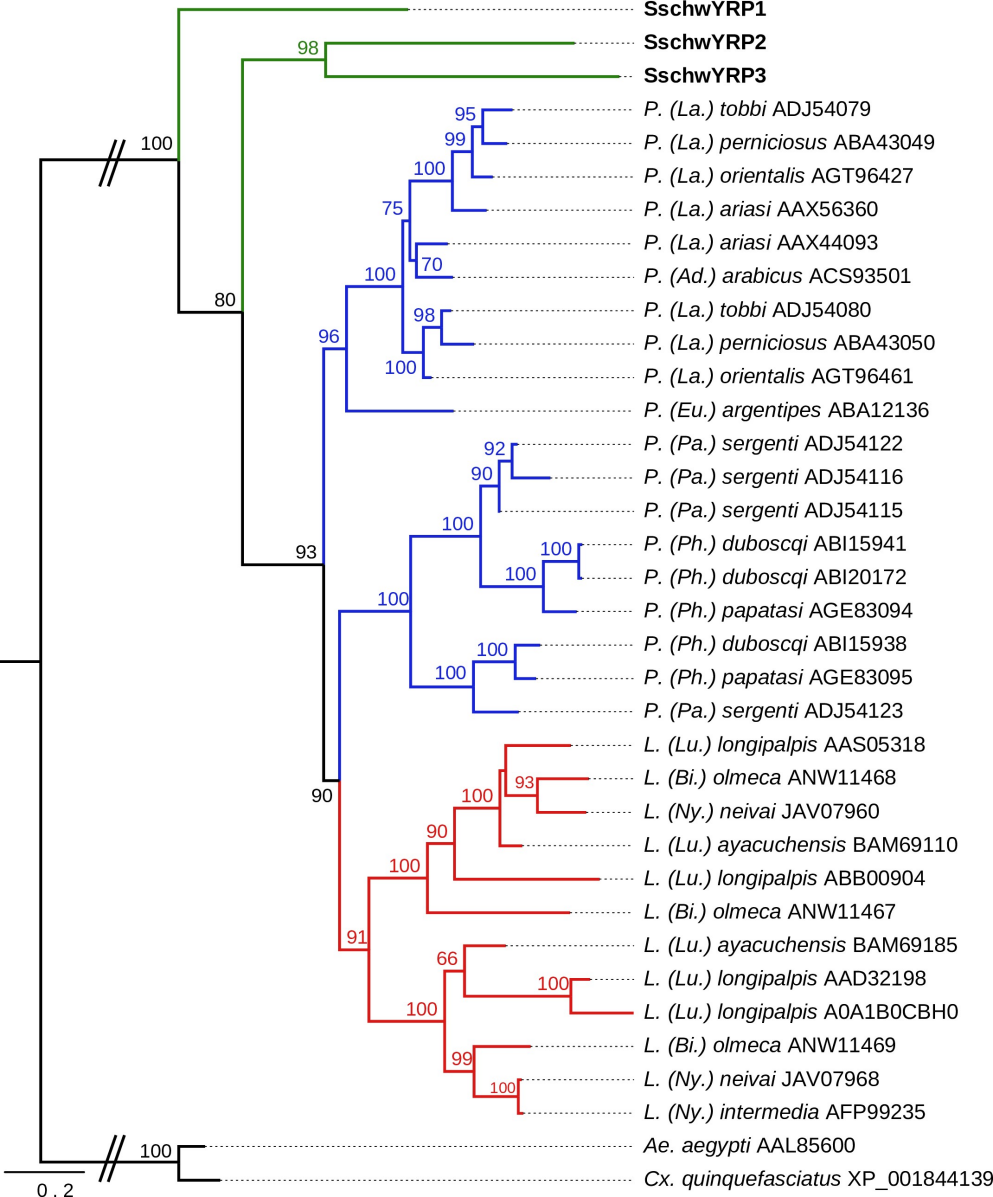
Phylogenetic comparison of yellow-related proteins (YRPs). The phylogenetic tree with 36 sequences was based on MAFFT alignment with BMGE (threshold 0.5) containing 359 aa sites, 312 parsimony-informative sites, 18 singleton sites and 29 constant sites. For rooting the tree, the *Aedes aegypti* (AAL85600) and *Culex quinquefasciatus* (XP_001844139) sequences were used (branches in black). For more detail see Fig 2 legend.

Phylogenetic analysis of YRPs revealed SschwYRPs as the paraphyletic basal group to other sand fly YRPs. Within the SschwYRPs, SschwYRP2 and SschwYRP3 generated distinct group to SschwYRP1 (Fig 6).

### Apyrase

Salivary apyrase was represented in the transcriptome by 3 full-length and 2 partial transcripts (Table 1, S2 Table).

The complete sequences were composed of 329 aa (SschwApy1), 333 aa (SschwApy2) and 339 aa (SschwApy3). One putative N-glycosylation site and one O-glycosylation site was predicted for SschwApy1 and SschwApy3, respectively. In the complete sequences of SschwApy the apyrase domain was detected with highly conserved Ca^2+^ binding sites. Nevertheless active site of enzyme was highly conserved only for SschwApy1 (S8 Fig). The sequence identity was 42.9% between SschwApy1 and SschwApy2, 39.8% between SschwApy1 and SschwApy3 and 47.9% between SschwApy2 and SschwApy3. The comparison of SschwApy sequences with other sand fly salivary apyrases revealed sequence identities between 38.9% and 50.8%.

**Fig 7:**
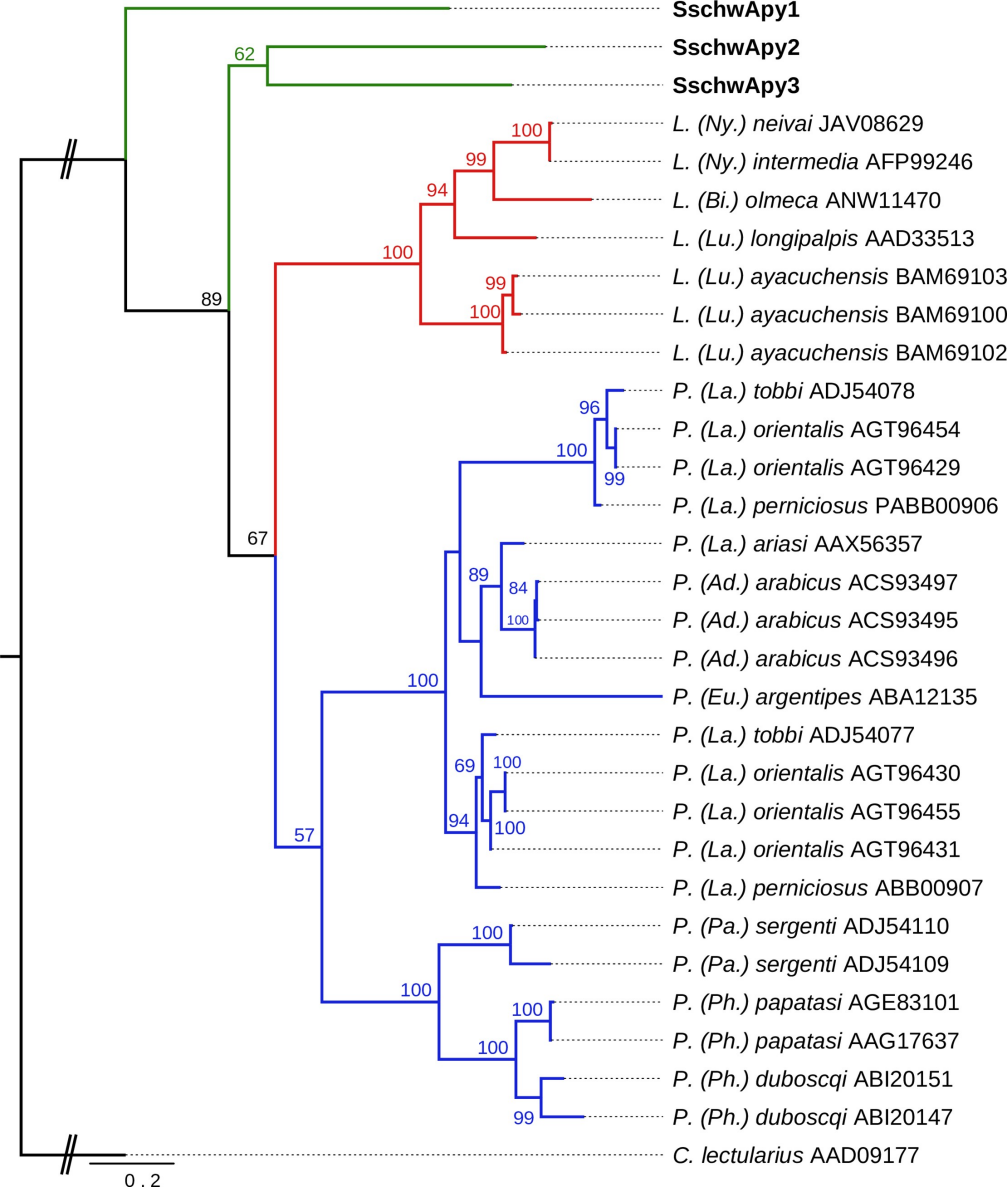
Phylogenetic comparison of salivary apyrase. The phylogenetic tree with 31 sequences was based on MAFFT alignment with BMGE (threshold 0.5) containing 303 aa sites, 236 parsimony-informative sites, 33 singleton sites and 34 constant sites. For rooting the tree, the *Cimex lectularius* (AAD09177) sequence was used (branch in black). For more detail see Fig 2 legend.

Based on the phylogenetic analysis, visualized on Fig 7, three analyzed SschwApy sequences form a sister clade to all available sequences from *Phlebotomus* and *Lutzomyia* sand fly species. Furthermore the SschwApy2 and SschwApy3 were clustering as one group separated from SschwApy1.

### Hyaluronidase

One full-length transcript of hyaluronidase, belonging to glycoside hydrolase family 56 –bee venom hyaluronidase, was detected in *S*. *schwetzi* transcriptome. The sequence contained 360 aa with the predicted Mw of 38.9 kDa for matured protein. Another two partial sequences of salivary hyaluronidase were identified (Table 1 and S2 Table).

In the complete sequence of SschwHya1 one putative O-glycosylation site was predicted, as well as 7 amino acid residues were putatively N-glycosylated. Further, the active sites of the enzyme were highly conserved in SschwHya1 (S9 Fig). The SschwHya1 shared sequence identity with other sand fly salivary hyaluronidases between 52.4% and 59.6%.

**Fig 8:**
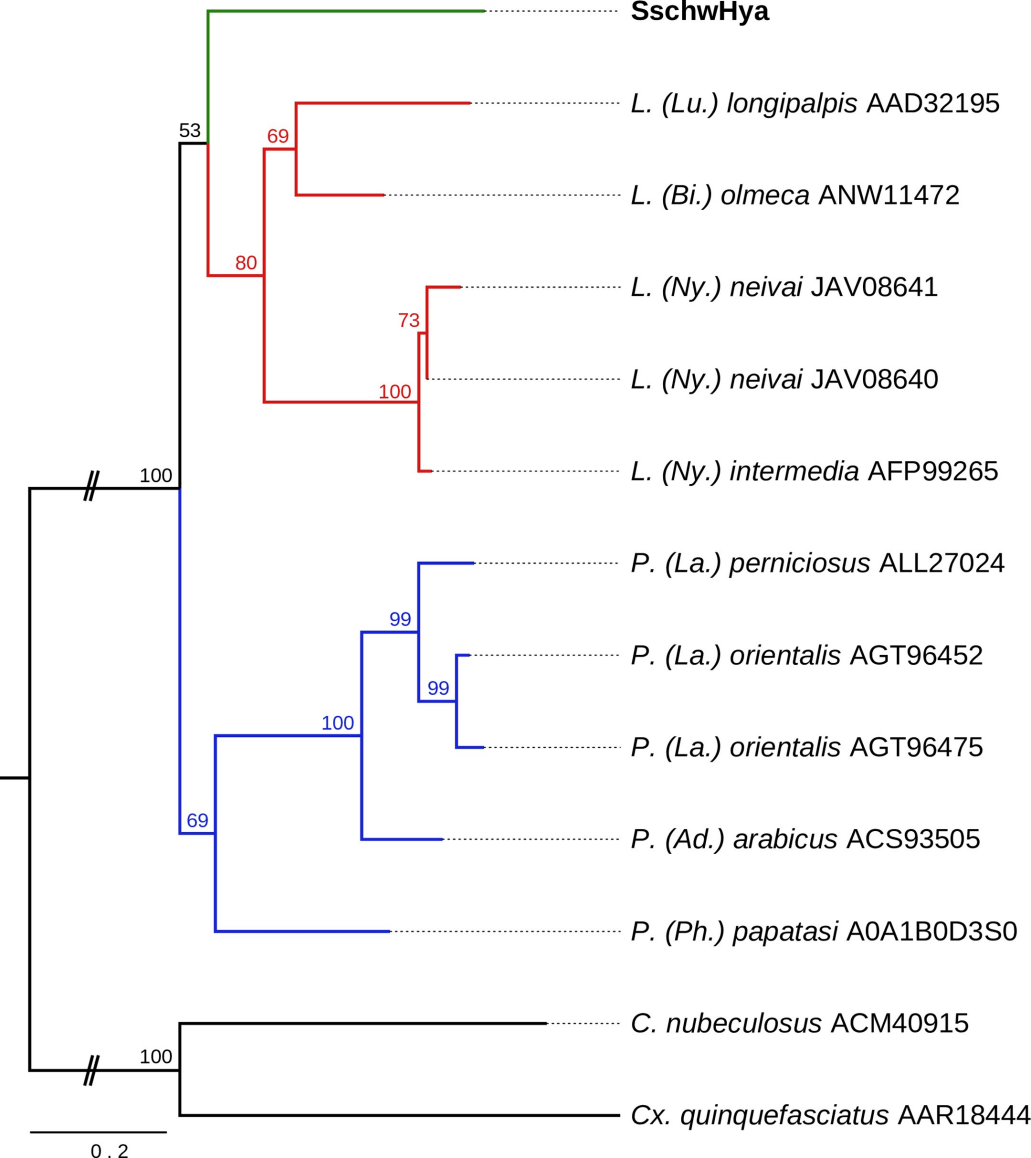
Phylogenetic comparison of hyaluronidase. The phylogenetic tree with 13 sequences was based on MAFFT alignment with BMGE (threshold 0.4) containing 270 aa sites, 154 parsimony-informative sites, 56 singleton sites and 61 constant sites. For rooting the tree, the *Culex quinquefasciatus* (AAR18444) and *Culicoides nubeculosus* (ACM40915) sequences were used (branches in black). For more detail see Fig 2 legend.

Phylogenetic analysis of other sand fly salivary hyaluronidases together with SschwHya1 showed that *S*. *schwetzi* hyaluronidase clustered as the sister group to hyaluronidase from the genus *Lutzomyia* (Fig 8).

### Other salivary enzymes

Complete and partial sequences were detected also for 5’-nucleotidase (s5nuc), adenosine deaminase (sADA), amylase (sAmy), endonuclease (sEnuc), pyrophosphatase (sPP) and phospholipase A2 (sPLA2). All detected full-length transcripts contained putative signal peptide cleavage site and the further annotations of each sequence are listed in Tables 1 and S2.

One full-length transcript of 5’-nucleotidase and 10 partial sequences of this enzyme were identified in the transcriptome. The complete sequence (560 aa residues) contained the putative signal peptide cleavage site. The putative Mw of matured protein of Sschw5nuc1 was 59.9 kDa and one positive N-glycosylation site (Asn102) was found in the sequence. The sequence consisted of two main domains i) calcineurin-like phosphoesterase ApaH type with conserved active sites and metal binding sites and ii) C-terminal 5’-nucleotidase domain. Both glycosylation and active and metal binding sites were depicted on the alignment in S10 Fig. The sequence identity of Sschw5nuc1 with other sand fly s5nuc was between 57.3% and 57.5%.

The adenosine deaminase was identified in one full-length transcript. The 515 aa long sequence contained 5 putative O-glycosylation sites (highlighted in S11 Fig) and the matured protein had Mw 57.6 kDa. Sequence identity of SschwADA1 compared with other known sand fly sADA was between 65.8% and 69.4% in the MAFFT alignment. The SschwADA1 contained specific adenosine/AMP deaminase domain belonging to metal-dependent hydrolases family. The amino acid residues forming active site were highly conserved through the SschwADA1 sequence (S11 Fig).

Three full-length transcripts of amylase were identified with protein sequence length 499 aa (SschwAmy1), 494 aa (SschwAmy2) and 509 aa (SschwAmy3), respectively. The predicted Mw of matured proteins were 53.9 kDa for SschwAmy1, 52.7 kDa for SschwAmy2 and 54.1 kDa for SschwAmy3. Both SschwAmy1 and SschwAmy3 had putative N-glycosylation sites (SschwAmy1 –Asn149, Asn416; SschwAmy3 –Asn159), while the sequence of SschwAmy2 possessed 6 putative O-glycosylation amino acid residues. According to MAFFT alignment the sequence identity between SschwAmy1 and SschwAmy2 was 59.2%, while between SschwAmy1 and SschwAmy3 was 67.7%. The sequence identity between SschwAmy2 and SschwAmy3 was lower (53.2%). In comparison with other sand fly amylases the SschwAmy reached sequence identities between 51.1% and 75.8%. All sequences of SschwAmy possessed glycoside hydrolase catalytic domain (family 13) and alpha-amylase C-terminal domain as other members of glycoside hydrolase superfamily. The active sites were conserved though the alignment as well as the Ca^2+^ binding sites. All the sequences annotations mentioned above are displayed on alignment in S12 Fig.

**Fig 9:**
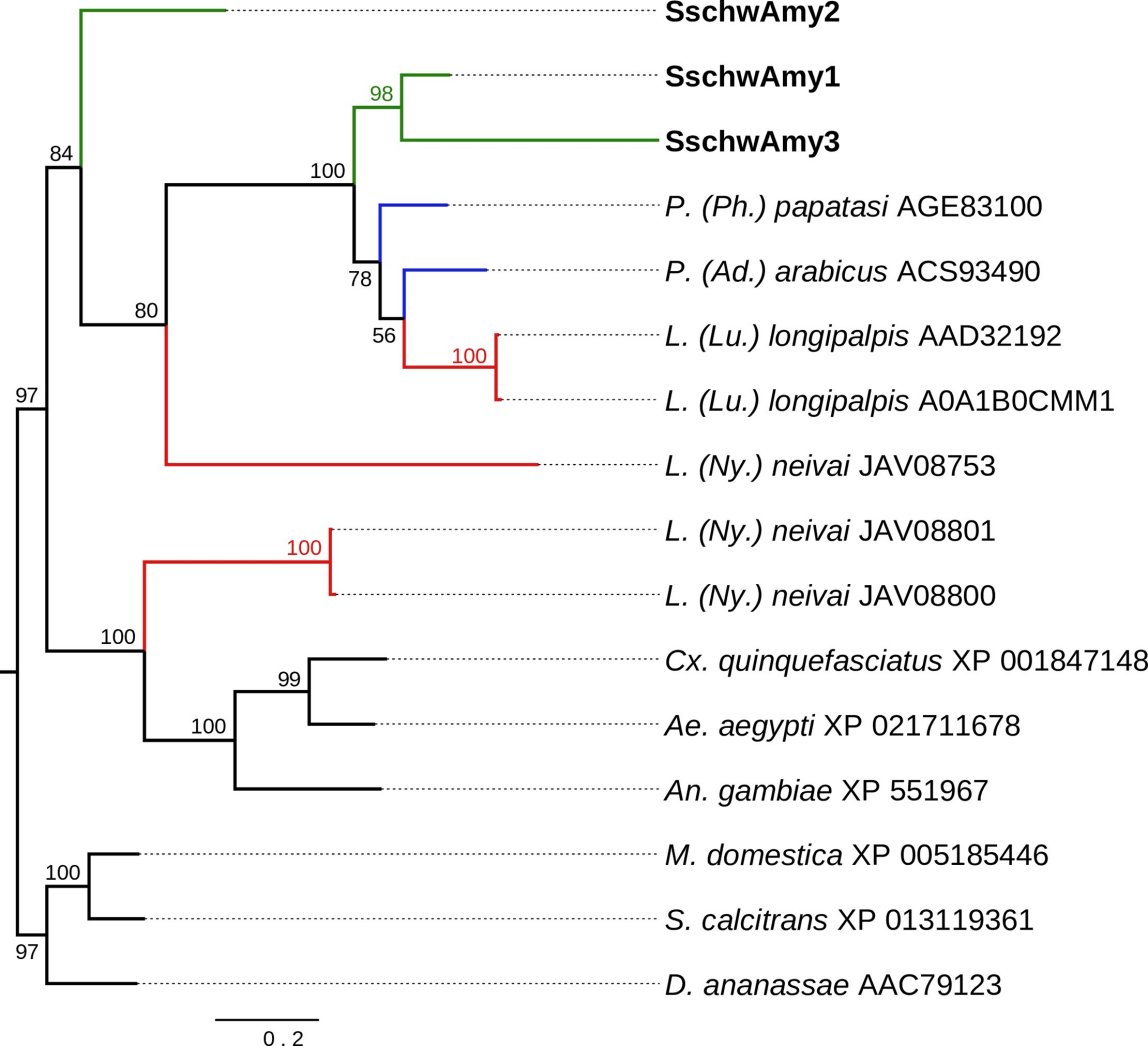
Phylogenetic comparison of salivary amylase. The phylogenetic tree with 16 sequences was based on MAFFT alignment with BMGE (threshold 0.5) containing 475 aa sites, 295 parsimony-informative sites, 51 singleton sites and 129 constant sites. By the black color are marked the additional sequences of sAmy from mosquito (*Culex quinquefasciatus* XP_001847148, *Aedes aegypti* XP_021711678, *Anopheles gambiae* XP_551967), drosophila (*Drosphila ananassae* AAC79123) and Muscidae (*Musca domestica* XP_005185446, *Stomoxys calcitrans* XP_013119361) species. Tree is unrooted. For more detail see Fig 2 legend.

The phylogenetic analysis of putative salivary amylase, depicted on Fig 9, does not correspond with the taxonomy position of analyzed sand fly species. Three SschwAmy proteins do not form a monophyletic group and constitute a sister clades to other known sand fly sAmy from *P*. *papatasi*, *P*. *arabicus* and *L*. *longipalpis* and/or to the putative alpha amylase from *L*. *neivai* (JAV0853).

Another salivary enzyme, endonuclease, was found in one full-length sequence with 468 aa and 14 partial sequences. The predicted Mw of secreted protein was 52.3 kDa and 10 putative O-glycosylation sites were predicted for SschwEnuc1 (S13 Fig). Comparing SschwEnuc1 with other sand fly sEnucs, the SschwEnuc1 shared with them from 32.4% to 39.2% of the sequence identity, with low level of sequence conservation. The exception was the DNA/RNA non-specific endonuclease domain which was detected in the SschwEnuc sequence, and is specific for other sEnucs. Even though the domain is truncated from C-terminus, the amino acid residues forming active site and Mg^2+^ binding site were conserved, as is visible on alignment in S13 Fig.

The phospholipase A2 with sequence of 383 aa was found in the *S*. *schwetzi* transcriptome. The secreted protein had putative Mw 42.1 kDa and 4 putative O-glycosylation sites and two N-glycosylation sites, which were highlighted in S14 Fig, were predicted for the sequence. The sequence identity with other sand fly sPLA2 was between 30.5% and 31.2%, especially the central part of the alignment was unconserved but on the C-terminus the SschwPLA2_1 sequence possessed conserved phospholipase A2 domain, specific for insects (Phospholipase_A2_2), with catalytic sites and metal binding sites (S14 Fig).

The complete protein sequence (442 aa) of pyrophosphatase (sPP) was identified. The putative Mw of 47.1 kDa and the putative 5 O-glycosylation and 4 N-glycosylation sites were predicted for the sequence. Comparing SschwPP1 with other sand fly sPP sequences by MAFFT alignment showed a sequence identity between 60.6% and 71.9%. SschwPP1 sequence contained typical phosphodiesterase domain with the active site, as well as the Zn binding sites, which were highly conserved. The sequence characterizations mentioned above are highlighted on the alignment in S15 Fig.

### Other salivary proteins

Other protein families rarely found in previously published sand fly salivary gland transcriptomes include 71kDa-like protein, protein with C-type lectin domain, ParSP80-like and SP16-like.

The 71 kDa-like protein family, so far detected only in NW sand fly species, and is annotated as angiotensin-converting enzyme. In *S*. *schwetzi* transcriptome one complete sequence and 12 partial sequences were detected (listed in Table 1 and S2 Table).

The complete sequence, Sschw71kDa1, possessed putative signal peptide cleavage site and had 626 aa, which resulted in Mw 70.9 kDa. The putative glycosylation sites displayed in S16 Fig, include one O-glycosylated residue, two N-glycosylated residues and one C-glycosylation residue. The domain typical for angiotensin-converting enzyme, which belonging to glu-zincin sub-group of metalloproteases, was detected in the sequence and the active site together with Zn binding site were highly conserved through the sequence (S16 Fig). According to MAFFT alignment Sschw71kDa shared identity with other *Lutzomyia* spp. homologues between 85.1% and 76.1%.

Two complete sequences of protein containing C-type lectin domain were identified (Tables 1 and S2 Table), both having a putative signal peptide cleavage site. The SschwCTL1 had 180 aa, its predicted molecular weight was 19.1 kDa with one putative N-glycosylation site (Asn36). The second protein, SschwCTL2, was 154 aa long and the molecular weight of its secreted form was 15.9 kDa with no putative glycosylation sites.

A single member of the SP16-like protein was detected. The complete sequence of SschwSP16 had 192 aa and a putative signal peptide cleavage site was identified in the sequence (Table 1 and S2 Table). The molecular weight of secreted protein was 17.6 kDa with no putative glycosylation site. In SschwSP16 no specific domains were detected. The sequence identity with other SP16-like proteins is quite low, between 29% and 38.8%.

One full-length transcript of a ParSP80-like protein was identified (Table 1, S2 Table). The SschwSP80 protein has 170 aa residues and putative signal peptide cleavage site followed by TRAP-delta (translocon-associated protein delta subunit) domain with no specific known function. The predicted molecular mass was 16.2 kDa and no putative glycosylation site was recognized. The SschwSP80 sequence identity with other sand fly homologues was detected between 82.4% and 74.6% according to MAFFT alignment.

Search through the transcriptomic data revealed two secreted proteins and two peptides which were homologous to infrequent sand flies’ putative salivary proteins (Table 1, S2 Table). The sequence of SschwSP60-like was annotated as homologous to *P*. *argentipes* SP60 and it contained 162 amino acid residues including putative signal peptide cleavage site. The molecular mass of mature protein was predicted as 15.2 kDa, but this protein sequence showed high glycosylation– 19 amino acid residues as positive for O-glycosylation and two residues for N-glycosylation. No specific domain or pattern was predicted for SschwSP60-like protein. The second putatively secreted protein showed homology with *P*. *duboscqi* 14.5 kDa salivary protein. The specific domain–transcription activator MBF2 (multiprotein bridging factor 2)–was detected in the sequence of SschwMBF2. This protein contained a putative signal peptide and its matured version was predicted to have 15.3 kDa molecular weight. Serine 88 was positive for putative O-glycosylation. Furthermore, two putatively secreted peptides, homologous to one *L*. *neivai* salivary secreted peptide, were identified in the transcriptomic data. The sequences of SschwPeptide1 and SschwPeptide2 were 89 aa long, contained putative signal peptide cleavage sites and had low molecular mass (7.7 kDa and 7.8 kDa). No specific domains or patterns were presented in the sequence.

Some of the salivary protein families were detected in *S*. *schwetzi* transcriptome only as partial sequences. The partial transcripts are listed with basic annotation in the S2 Table which includes the members of PpSP32-like (silk proteins), ParSP25-like, ParSP17-like, salivary secreted ribonuclease (sRNase) and a conserved secreted peptide homologous to *L*. *ayacuchensis* Lol38.8.

### Expression differences between sand flies fed on mice and geckos

Genes differentially expressed on the level of RNA between S-M and S-G lineages were identified and BLASTp annotated. From the whole annotated arthropod RNA-seq dataset, 150 transcripts of the salivary protein families were identified (S3 Table). The majority of the most abundant transcripts in both lineages (60 out of 70) were members of salivary protein families: YRPs, OBPs, lufaxins, Ag5rs, ParSP17-like and a PpSP32-like proteins, apyrase, hyaluronidase, endonuclease, protein with CTL domain and transcript homologous to Lol38.8.

First we analyzed only transcripts annotated as arthropod genes. The analysis step-by-step is displayed on S17 Fig. In the S-G lineage, 40 transcripts of the salivary protein families were up-regulated (EDGE fold change ≥ 1.5) (S17A, I Fig). The statistical evaluation of EDGE fold change (p-value < 0.05) of the up-regulated S-G transcripts did not revealed any salivary transcript (S17A, II Fig).

In the S-M lineage, the group of up-regulated transcripts (EDGE fold change at least 1.5) included 20 transcripts homologous to salivary proteins (S17B, I Fig). The statistical evaluation of EDGE fold change revealed two salivary sequences namely: SschwSP15_5 and SschwLuf3 (S17B, II Fig).

Later, we expanded the dataset and analyzed all differentially expressed transcripts (EDGE fold change ≥ 1.5, FDR corrected p-value < 0.05). This dataset contained eight differentially expressed transcripts (marked as DET1-8, Table 2). Only three of these sequences (DET2, DET6 and DET7) were annotated as arthropod sequences, while DET2 corresponded with protein SschwSP15_5. Other sequences were manually annotated using the online BLASTx algorithm, but the homology of DETs with known sequences was low (Table 2). While any of salivary transcripts was not up-regulated more than 30 times (either in the S-G or the S-M lineage), some of the DET transcripts were up-regulated for more than 1,000 times. According to RNA-seq analysis, seven transcripts (DET1-6, DET8) were up-regulated in the S-M lineage and one (DET7) in the S-G lineage.

**Table 2:** Differentially expressed transcripts (DETs) annotation.

	GenBank Accession number	BLAST match to NR database					Nucleotide seq. length	Lineage up-regulation	EDGE test: Fold change	S-M lineage expression value (RPKM)	S-G lineage expression value (RPKM)
		Species of the BLAST match	Accession number	Annotation	E-value	Seq. identity [%]					
**DET1**	MN605409	*Rhagoletis zephyra*	XP_017480351	**uncharacterized protein**	3.3	50	218	S-M	1206	1120.53	0.88
DET2*	MN605297	*Phlebotomus orientalis*	AGT96472	SP15-like salivary protein	0.001	34	253	S-M	576	145.52	0.16
**DET3**	MN605411	*Acropora digitifera*	XP_015766698	uncharacterized protein	0.29	28	605	S-M	752	107.05	0.02
**DET4**	MN605412	No hits	-	-	-	-	239	S-M	393	56.01	0.03
**DET5**	MN605413	*Pseudomonas fulva*	AEF21400	**hypothetical protein Psefu_1424**	6.9	36	229	S-M	236	105.62	0.36
**DET6**	MN605414	*Lutzomyia longipalpis*	AAS17937	16.4 kDa salivary protein	0.15	32	265	S-M	64	98.29	1.53
**DET7**	MN605415	*Aedes aegypti*	XP_01655722	general odorant-binding protein 56a	4E-21	32	535	S-G	137	0.20	47.80
**DET8**	MN605416	*Lingula anatina*	XP_023930744	**macrophage mannose receptor 1-like**	0.18	39	317	S-M	474	108.65	0.12
**Reference transcript 1**	MN605417	*Lutzomyia neivai*	JAV13762	putative actin, partial	7E-220	100	1,131	non		136.6	158.5
**Reference transcript 2**	MN605410	*Lutzomyia neivai*	JAV12252	putative glycerol-3-phosphate dehydrogenase/dihydroxyacetone 3-phosphate reductase	2E-100	98	1397	non		7.6	7,0

Annotation of differentially expressed transcripts from RNA-seq and reference transcripts, which were used for RT-qPCR. Table columns indicates: Name of transcript, NCBI GenBank nucleotide accession number, BLAST match to NCBInr database, nucleotide length of transcript, lineage in which was the transcript up-regulated, fold change difference in expression between lineages, average expression from three replicates for both S-M and S-G lineage.

*DET2 is SschwSP15_5

**Fig 10:**
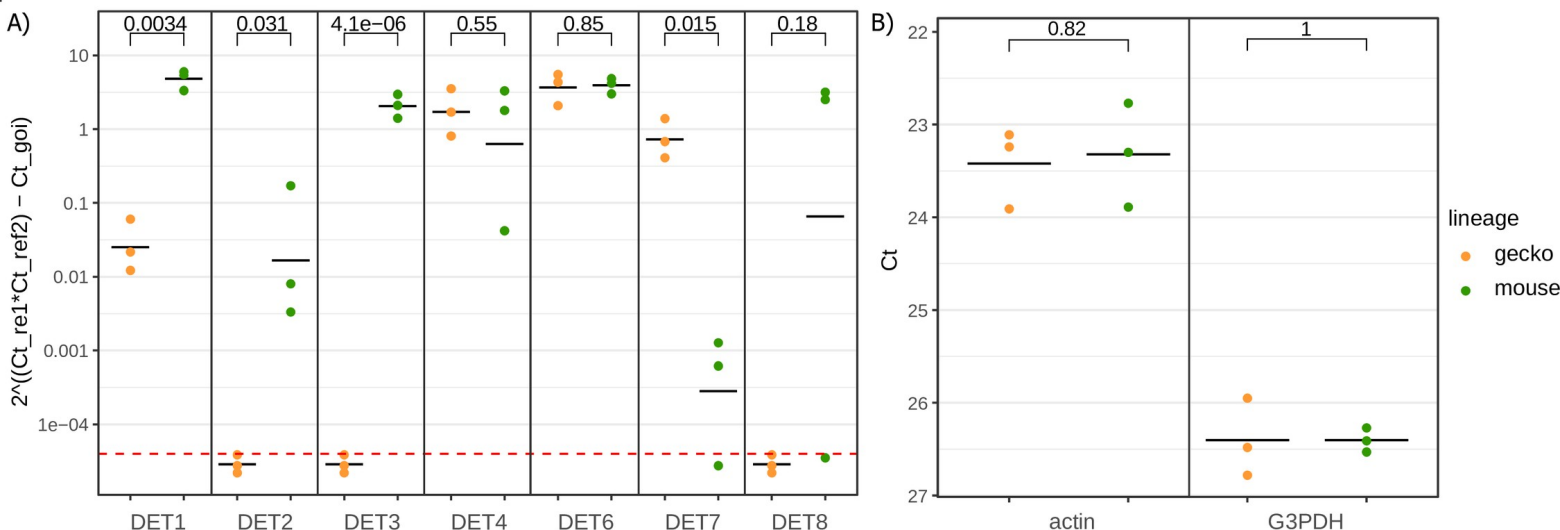
Differential gene expression in *S*. *schwetzi* lineages feeding on mice (S-M) and geckos (S-G). Differential expression of seven transcripts was evaluated by RT-qPCR method. (A) The obtained C_T_ values were relatively quantified according to the 2^-ΔΔCT^ method [45] using actin and G3PDH as the reference transcripts. The relative gene expression values of all samples were calibrated to mean of C_T_ values measured for each transcript from S-G lineage. Each column of chart contains relative expression of one differential expressed transcript (DET1–8) for both S-G (orange points) and S-M (green points) cDNA, measured in three independent replicates. The mean of relatively quantified values is depicted by horizontal line in black color. The expression values below the red line were under the detection RT-qPCR limit (C_T_ = 40). The expression difference between S-G and S-M were compared by t-test and p-values are shown above the square bracket. (B) The C_T_ values for the reference transcripts (actin and G3PDH) for both S-G (orange points) and S-M (green points) cDNA, measured in three independent replicates.

Subsequently we confirmed RNA-seq results accuracy by RT-qPCR. Seven DETs were chosen for RT-qPCR tests (DET5 was excluded because it was annotated as a prokaryotic sequence). The RT-qPCR results confirmed significant up-regulations of three transcripts (DET1, DET2, and DET3) in the S-M lineage and up-regulation of DET7 in the S-G lineage (Fig 10). There was no significant difference detected in the expression of DET4, DET6 and DET8.

### Salivary gland proteome

Mass spectrometry data analysis of the whole salivary glands identified 1,153 proteins. The quality-filtering of these data resulted in the final dataset of 631 proteins, selected for subsequent analysis. Out of these, 85 proteins were annotated as salivary (S18 Fig). The group of the top 54 enriched proteins (with intensity 1 × 10^9^), contained members of the main salivary protein families, namely YRPs, OBPs, Ag5r, ParSP17, PpSP32, lufaxin, as well as members of typical salivary enzymes i.e. apyrase, endonuclease and adenosine deaminase. The proteomic data for salivary proteins including the LFQ intensities and their comparison between the two lineages are show in S4 Table.

**Fig 11:**
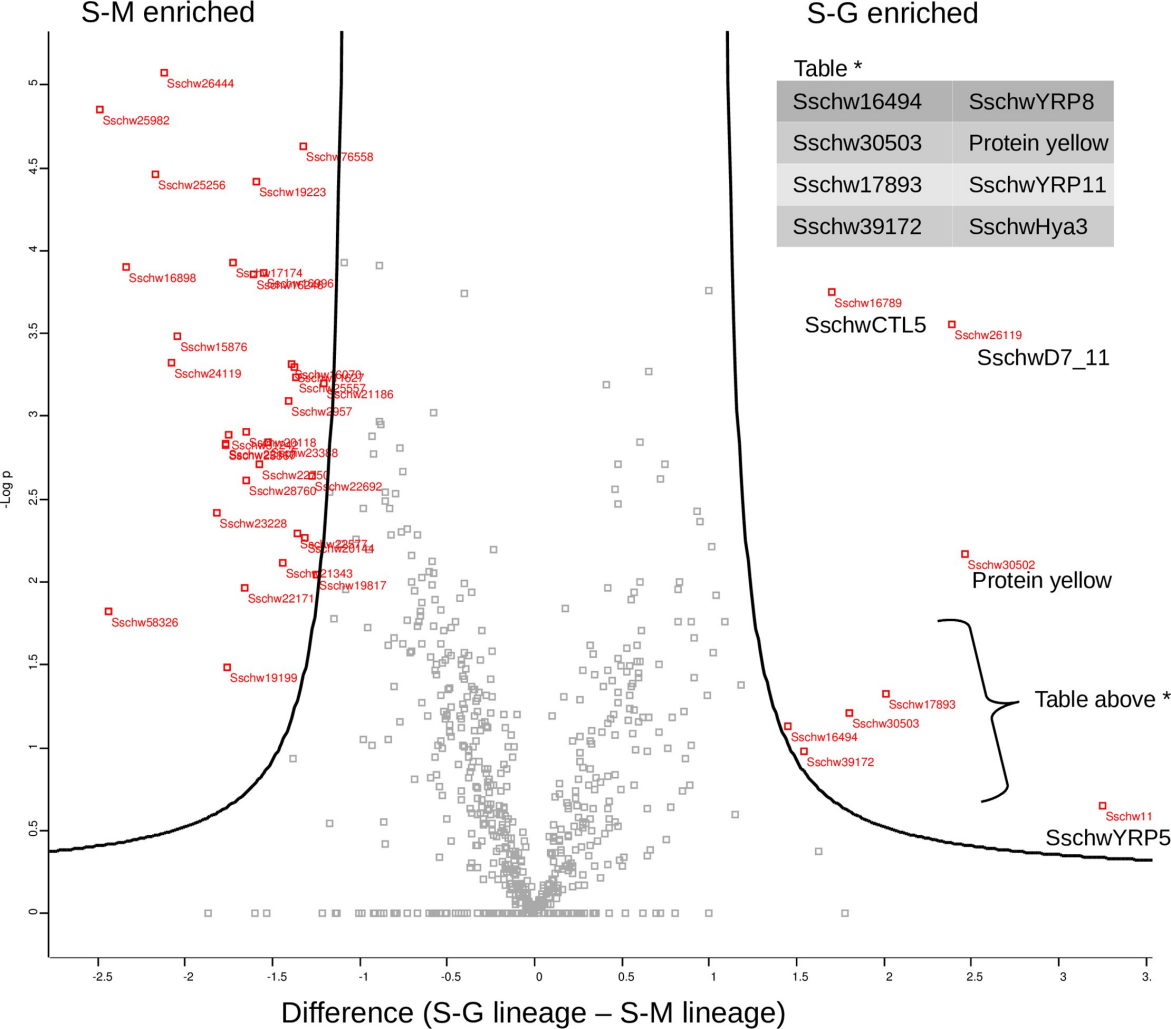
Volcano plot with protein enrichment from mass spectrometry analysis of *S*. *schwetzi* lineages feeding on mice (S-M) and geckos (S-G). Differences in protein enrichment between S-M and S-G lineage proteome with Student’s t-test evaluation. In S-M lineage proteome there are 35 proteins significantly enriched. In S-G lineage proteome are 8 proteins significantly enriched (including six salivary proteins and two non-salivary putative proteins yellow-d).

The proteome analysis revealed 586 proteins shared by both lineages. Moreover, 43 proteins were detected in the S-M sialome exclusively, including one salivary SschwYRP20 protein. Two proteins were found only in the sialome of SG lineage–one salivary protein containing a CTL domain (SschwCTL4) and one with a C-terminal tandem repeat domain in type 4 procollagen (S18A Fig). When comparing the protein enrichment (LFQ intensities) between lineages, 359 and 227 proteins were found to be enriched in the S-M and S-G sialomes, respectively. Importantly, 10 of the 359 enriched proteins in the S-M sialome and 66 out of 227 enriched proteins in the S-G sialome were annotated as salivary. At a cut off 0.6 for the LFQ intensity, 113 proteins in the S-M sialome were found to be enriched at least 1.5 ×; none of them, however, were homologous to the salivary proteins. In contrast, 58 proteins in the S-G sialome were shown to be enriched at last 1.5 ×, of which 36 were identified as salivary. Finally, statistical evaluation of both sialomes revealed 35 proteins to be significantly enriched in the S-M sialome–all annotated as housekeeping proteins (Fig 11), compared to 8 significantly enriched proteins in the S-G sialome, of which six were annotated as salivary (Fig 11). The distributions of enriched proteins within the lineages and the analysis step-by-step are highlighted in S18B Fig. The putative actin and G3PDH (transcripts which were used as reference transcript for RT-qPCR) were used as the normalization control between the lineages. The LFQ intensities of both reference proteins were very similar for S-G (29.5 and 24.7 respectively) and SM (29.2 and 25.4 respectively) lineages.

**Fig 12:**
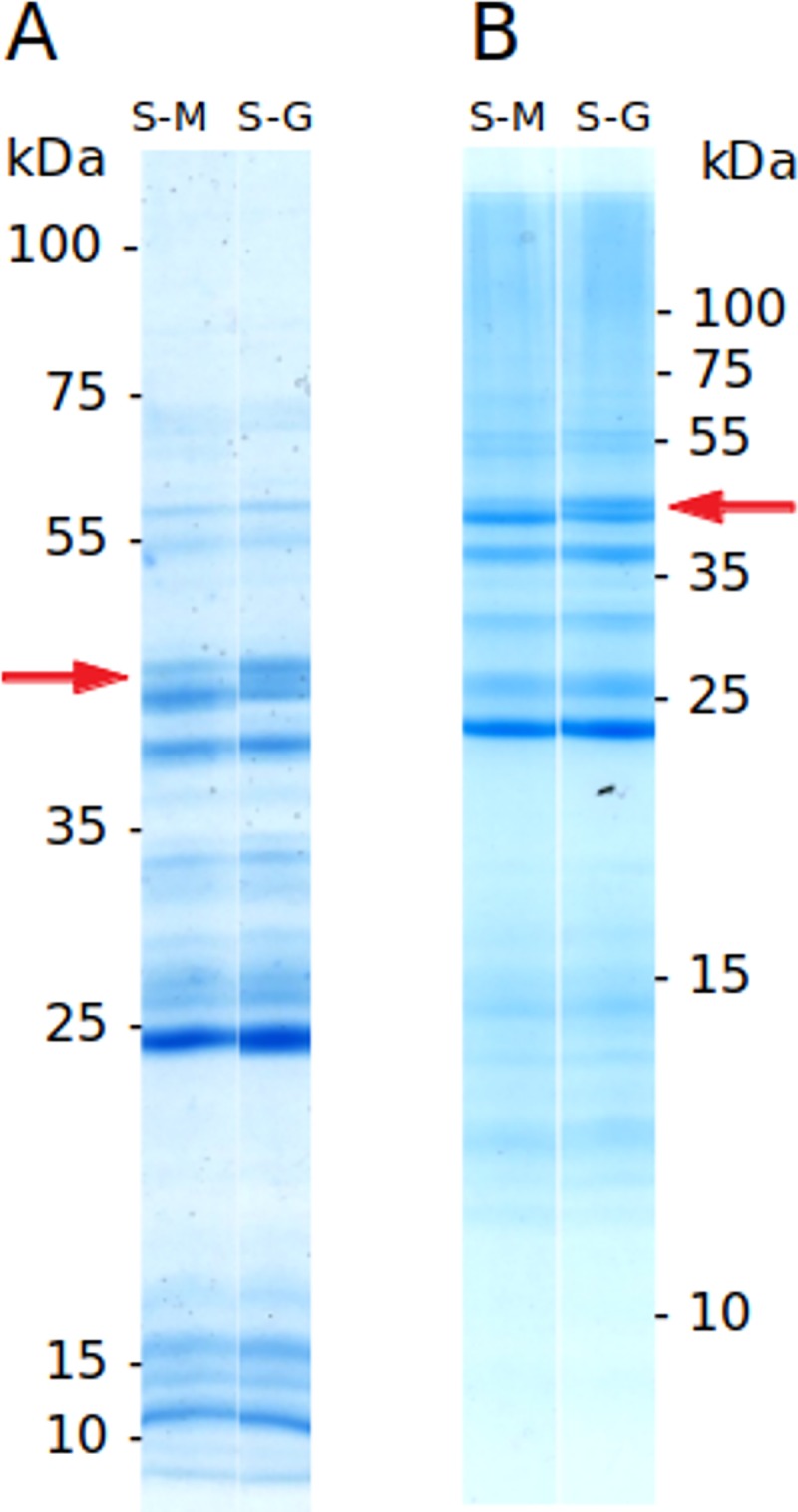
Salivary glands proteins from two *S*. *schwetzi* lineages maintained on different blood-meal sources geckos (S-G) and mice (S-M): SDS PAGE electrophoresis under reducing conditions. (A) separation on 10% polyacrylamide gel, (B) separation on 15% polyacrylamide gel. Molecular weights in kilodaltons are indicated and red arrow marks the band 40–45 kDa.

The electrophoretic separation of both SM and S-G salivary gland proteins revealed very similar profiles. The only visible difference was a 40-45 kDa band (red arrow on Fig 12), enriched in the S-G sample.

### Apyrase assay

The apyrase activities were similar in the SGHs from both *S*. *schwetzi* lineages. The maximum of substrate hydrolysis at pH 8.5 was identical for both lineages and both substrates (S19 Fig). Mean ATPase and ADPase activities determined per a pair of salivary glands as well as per milligram of total proteins are summarized in Table 3.

**Table 3:** Salivary apyrase and hyaluronidase in two *S*. *schwetzi* lineages maintained on different blood-meal sources, geckos (S-G) and mice (S-M).

		S-G	S-M
Total protein in μg/gland pair*		0.44 ± 0.06	0.42 ± 0.06
Mean specific apyrase activity at 37°C, pH 8.5*			
mUnits/pair of glands	ATPase	28.40 ± 4.62	29.50 ± 4.20
	ADPase	31.61 ± 4.84	34.26 ± 4.81
Units/mg of total protein	ATPase	64,55 ± 10.5	70.23 ± 10.0
	ADPase	71.84 ± 11.0	81.57 ± 11.5
ATPase/ADPase ratio		0.9	0.86
Mean specific hyaluronidase activity at 37°C, pH 5.0*			
rTRU/pair of glands		0.3713 ± 0.07	0.5338 ± 0.1
rTRU/mg of total protein		0.844 ± 0.16	1.213 ± 0.24

* Results represent the mean ± SD of ten independent measurements

### Hyaluronidase activity

**Fig 13:**
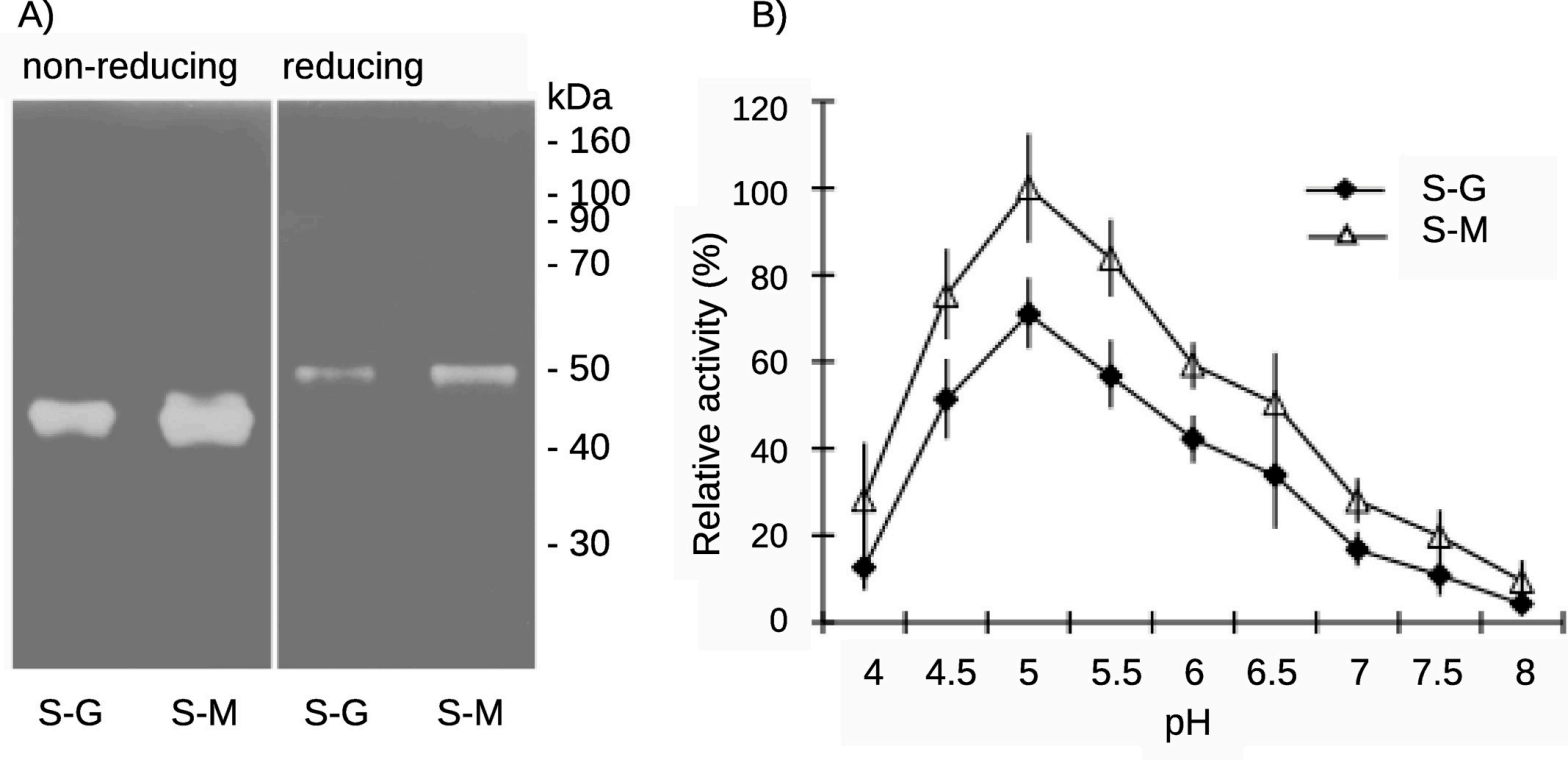
Visualization and comparison of the hyaluronidase activities in two *S*. *schwetzi* lineages maintained on different blood-meal sources, geckos (S-G) and mice (S-M). (A) Visualization and comparison by SDS PAGE zymography. SDS PAGE gel contained 10% polyacrylamide gel with incorporated 0.002% HA. SGHs of two *S*. *schwetzi* lineages maintained on different blood meal sources geckos (S-G) and mice (S-M) were tested under non-reducing and reducing conditions. (B) Comparison of the pH dependence of salivary hyaluronidase activity from S-G and S-M lineages. Results represent the mean ± SD of five independent measurements. The difference in relative activities for different buffer systems at pH 7 was lower than 1%.

The analysis by SDS PAGE zymography on hyaluronan substrate gels revealed pronounced hyaluronidase activities in saliva of both lineages. Both enzymes were visualized as monomers, with Mw about 43–44 kDa in non-reduced environment, and remained active under reducing conditions, with estimated Mw at 50 kDa. However, the degradation of HA substrate was substantially higher in SGHs from the lineage maintained on mice (Fig 13A).

The assay on microtitration plates showed maximum of HA hydrolysis at pH 5.0 in both lineages studied however, significant differences were detected in measured enzyme activities. At the optimal pH, hyaluronidase activity of the S-M lineage was about 30% higher than the activity of the S-G lineage (Table 3). Moreover, elevated hyaluronidase activity in the S-M lineage was detectable within a broad pH range, from 4.0 to 7.5 (Fig 13B).

## Discussion

This is the first study describing salivary components of phlebotomine sand fly of the genus *Sergentomyia*. Previously, sand fly salivary gland transcriptomes have been published for 13 sand fly species (reviewed in [11]), all made by sequencing phage cDNA libraries; that technology allowed identification of the low numbers of high quality transcripts and secreted full-length proteins (from 535 to 1,765 transcripts and from 15 to 64 full-length proteins per transcriptome) [13,59]. The only currently available *L*. (*Ny*.) *neivai* sialome and mialome sequences done on Illumina platform have not been published yet. Our BLASTp annotation showed that 51.5% (10,193 sequences) of *S*. *schwetzi* were homologous to *L*. (*Ny*.) *neivai* proteins.

Main salivary protein families detected in all sand fly species studied so far include yellow-related proteins, D7-related proteins, PpSP15-like proteins, lufaxin proteins, salivary apyrase, and PpSP32-like proteins [59]. Here, in *S*. *schwetzi*, we demonstrated the presence of all these protein families. In addition, we found several proteins previously considered as specific for the genus *Phlebotomus* among sand flies [60]: particularly salivary pyrophosphatase, phospholipase A2, ParSP25-like proteins and SP16-like proteins. Interestingly, we also found a salivary ribonuclease (partial sequence, QHO60797), which was previously detected only in transcriptomes of mosquitoes salivary glands.

Salivary proteins or peptides previously identified exclusively in transcriptomes of NW sand flies include vasodilatory peptide maxadilan [61], salivary 5′-nucleotidase, an enzyme responsible for cleaving AMP to adenosine [62], SALO anti-complement proteins [63], the RGD-containing peptides with an anti-platelet aggregation function [64] and several protein families with an unknown biological function, like the C-type lectins, 71 kDa salivary protein, spider-toxin-like and ML domain proteins [59,62,65–67]. Even though the *S*. *schwetzi* is a sand fly species from OW, we identified in its salivary gland transcriptome salivary 5’-nucleotidase, 71 kDa salivary protein and the protein containing C-type lectin domain.

Three sequences found homologous to protein with C-type lectin domain (SschwCTL4, SschwCTL5 and SschwCTL6) which had high expression values in transcriptome (S3 Table), were detected also in the proteome (S4 Table). Usually, the proteins with C-type lectin domain are immunity-related molecules associated with an activation of complement system (reviewed in [68]). On the other hand, the snake venom C-type lectins showed anti-coagulation (by binding FX and FIX) and anti-thrombotic (by inhibition of collagen-induced platelet activation) functions [69]. However, the function of sand fly proteins with C-type lectin domain remains unknown.

The phylogenetic and sequence analysis showed that most of the *S*. *schwetzi* salivary protein sequences are more divergent, compared to the *Lutzomyia* and *Phlebotomus* proteins. However, the results of phylogenetic comparison of *S*. *schwetzi* salivary protein families (antigen 5-related proteins, lufaxins, YRPs, and apyrases) were in accordance with the established sand fly phylogeny [70,71]. The *S*. *schwetzi* OBPs showed high sequence divergence, which is together with high gene duplication rates, typical for the sequences belonging to D7-related and PpSP15-like families [60]. On the other hand, *S*. *schwetzi* salivary enzymes identified in the transcriptome were more conserved, likely due to their enzymatic function. The exception in this trend was found in the *S*. *schwetzi* endonuclease and phospholipase A2 sequences, which were longer than other sand flies’ homologues. Other *S*. *schwetzi* proteins usually clustered as basal group to other sand fly proteins, and some of the *S*. *schwetzi* proteins created paraphyletic groups. This phylogenetic trees structure can be caused by selective pressure of different feeding preferences of *Sergentomyia* sand flies, which preferably bite reptiles in contrast to *Lutzomyia* and *Phlebotomus* sand flies, which feed mostly on mammals. Despite all *Sergentomyia* species studied prefer reptiles, species-specific differences were described [72]. In Ethiopia, *S*. *schwetzi* feed not only on reptiles but also on mammals, Gebresilassie *et al*. 2015 found engorged females on cow, donkey and goat [73], while Yared and colleagues detected human and canine blood in *S*. *schwetzi* [74].

In *S*. *schwetzi* transcriptome we identified one homologue of salivary hyaluronidase and three homologues of apyrase. The activities of both these enzymes and their pH optima were already described in *S*. *schwetzi* saliva by Volfova and Volf (2018) [14]. The sequence of SschwApy1 was conserved in all active sites, but the function of SschwApy2 and SschwApy3 can be questioned due to their amino acids replacements in the penultimate and last position of the active site sequence. Comparison of the salivary apyrase activities between S-G and S-M lineages did not show any significant differences. On the other hand, interesting differences were found in hyaluronidase activities using two methods (SDS PAGE zymography, microtitration plates assay). In both tests, the HA cleavage activity was significantly higher in S-M lineage, the quantitative assay on microtitration plates repeatedly showed about 30% difference in the hyaluronidase activity. The adaptation of sand flies to feed on mammals is associated with the length of their mouthparts and the depth of skin penetration due to the relatively thick and stiff epidermis of mammalian skin [75]. However, various species with a rather short labrum e.g. *P*. *argentipes* and some *Sergentomyia* species are able to obtain successfully blood from the mammal hosts [76]. Increasing the tissue permeability of mammalian skin, HA degrading enzymes might represent an efficient tool for sand flies originally adapted to non-mammalian hosts to get access to blood.

Yellow-related proteins (YRPs) of *Lutzomyia* and *Phlebotomus* genera are known to have amine-binding function [77,78]. The amine-binding affinity of YRPs depends on the conservation of sequence regions, especially on eleven amino acid residues, which are responsible for creating amine binding pocket and that are conserved across the *Phlebotomus* and *Lutzomyia* species [79]. However, the SschwYRPs sequences forming the amine binding pocket are more variable (S6 Fig), which may result in inability to bind biogenic amines. Thus, SschwYRPs functions would be interesting topic for future studies.

Besides the description of *S*. *schwetzi* salivary proteins, we focused also on the comparative transcriptomic and proteomic analysis of salivary glands from sand fly females from two lineages maintained on different vertebrate hosts, mice and geckos, animals with different haemostasis. For example, all vertebrates possess C3 component and factor B, which is necessary for creation of C3b-B proconvertase. Reptile complement shares several features with the mammalian one, like activation by both alternative and classical pathway (reviewed in [80]). On the other hand, all reptiles lack genes coding for FXI, which active form (FXIa) is responsible for activation of coagulation factors leading to transformation of prothrombin to thrombin (reviewed in [81]).

The RNA-seq analysis of *S*. *schwetzi* salivary gland transcriptome revealed two salivary transcripts, one belonging to lufaxin salivary protein families (SschwLuf3) and another to PpSP15-like salivary protein family (SschwSP15_5), which were significantly up-regulated in S-M lineage (S3 Table, S17 Fig). Enrichment of SschwLuf3 was confirmed by proteomic analysis but biological function of the molecule is questionable as its expression is relatively low in both lineages (S3 Table). The SschwSP15_5 transcript up-regulation in S-M lineage might be connected to variability of coagulation pathway between geckos and mice, but its function remains suspicious as its expression was very low (S3 Table).

In order to compare saliva composition between S-G and S-M lineages, we also did SDS-PAGE gels and mass spectrometry of salivary glands. The single visible difference between electrophoretograms of S-M and S-G lineages was found in the Mw between 40 and 45 kDa: the protein band was more pronounced in S-G sample (Fig 12). In this Mw range several sand fly salivary proteins including hyaluronidase, phospholipase A2, pyrophosphatase and YRPs. The best candidate is probably one of three SschwYRPs (SschwYRP5 with highest LFQ intensity value) as their enrichment in S-G lineage was confirmed by mass spectrometry analysis, see below (Fig 11).

The mass spectrometry analysis revealed six salivary proteins significantly enriched in S-G sialome (S18 Fig, S4 Table). Three of enriched proteins were homologous to sand flies’ YRPs, but all of them were detected only as the partial sequences (SschwYRP5, SschwYRP8 and SschwYRP11). Last three enriched proteins were partial sequences homologous to sand fly hyaluronidase (SschwHya3), D7-related protein (SschwD7_11) and protein with C-type lectin domain (SschwCTL5) (Fig 11).

As we mentioned above, two different methods revealed higher hyaluronidase activity in the saliva of S-M lineage but the comparison by RNA-seq analysis did not reveal any significant differences in expression of the hyaluronidase transcripts. Therefore, there is a possibility of later expression of SschwHya1 than in first two days after adult emergence. However, the salivary proteins expression dependence on sand fly age was showed only for *P*. *papatasi* SP44 protein (YRP), while the expression of this protein was higher for younger and lower for older sand flies which were fed on sugar [82]. In contrast, all three homologues of hyaluronidase (one complete and two partial proteins) were more abundant in proteome of S-G lineage, where SschwHya3 was significantly enriched. This contrasting result can be due to coexistence of different homologues of the enzyme, the one enriched in S-G lineage has no or low activity and the second one is fully active. Thus, it is possible that the protein SschwHya3 which is significantly enriched in S-G lineage, has less or no activity, and the active homologues of enzyme could be SschwHya1 or SscheHya2. Similar trend was recently revealed in salivary yellow-related proteins, where one homologue has high amine-binding activity and the other has very low activity [78]. Furthermore, the difference in hyaluronidase activity can be explained by different glycosylation of SschwHya homologues, but this we were not able to compare, because two of three SschwHya are only partial sequences. This crucial role of glycosylation for hyaluronidase activity was revealed for *L*. *longipalpis* hyaluronidase [83].

The SschwD7_11 was significantly enriched in S-G proteome (Fig 11) and its transcript was highly up-regulated in S-G transcriptome as well. The function of D7-related proteins, known also as large OBPs, was firstly proved for their mosquito homologues. The *An*. *stephensi* D7-related protein inhibits the *intrinsic* coagulation pathway by binding FXII and high molecular weight kininogen (HK), which are together with prekallikrein at the beginning of *intrinsic* coagulation pathway [84]. Other homologues of D7-related proteins, from *Ae*. *aegypti* and *An*. *gambiae*, are able to bind biogenic amines and eicosanoids [85–87]. Recently, the sand flies’ D7-related were identified as binders of cysteinyl leukotrienes and thromboxane A2 [88]. Despite these new findings, we are not able to connect the SschwD7_11 up-regulation to its specific function on reptile haemostasis or coagulation.

In conclusion, our study significantly expands the knowledge on salivary proteins of neglected sand fly genus *Sergentomyia*. Thanks to phylogenetic and sequence analysis we found, that salivary proteins of *S*. *schwetzi* are more diverse from *Phlebotomus* and *Lutzomyia* homologues which can be due to adaptation to preferable vertebrate host–reptiles. To support this theory, we compared two *S*. *schwetzi* lineages adapted to different hosts (gecko and mice). This comparison revealed significantly higher hyaluronidase activity, which can be caused by different properties of mice’s skin comparing to geckos’. Further we showed an up-regulated expression of transcripts for PpSP15-like protein and lufaxin in mice lineage, which might be due to different haemostasis of these two animals. Last but not least, the transcriptomic analysis also demonstrated unique salivary secreted ribonuclease, the enzyme previously found only in mosquitoes.

## Supporting information

S1 Fig Raw gels image for Figs 12 and 13. (PDF)

S2 Fig Functional gene ontology (GO) classification of the *S*. *schwetzi* salivary gland transcriptome. The percentage and distribution of top-level GO-terms are portrayed in the three categories: cellular component, molecular function and biological process. Transcripts annotated from whole transcriptome sequences dataset are indicated by blue bar (7,749 sequences) and transcripts annotated from arthropod sequences subset are in yellow (5,937 sequences).
(PDF)

S3 Fig Multiple sequence alignment of sand flies’ antigen 5-related proteins. Multiple sequence alignment of *S*. *schwetzi* antigen 5-related proteins with chosen sand flies’ antigen 5-related proteins. Name of sequence include sand fly species shortcut (P.tob–*P*. *tobbi*, P.ser–*P*. *sergenti*, P.per–*P*. *perniciosus*, P.ori–*P*. *orientalis*, P.ari–*P*. *ariasi*, P.ara–*P*. *arabicus*, P.pap–*P*. *papatasi*, P.dub–*P*. *duboscqi*, P.arg–*P*. *argentipes*, L.lon–*L*. *longipalpis*, L.aya–*L*. *ayacuchensis*, L.olm–*L*. *olmeca*, L.nei–*L*. *neivai*) and GenBank accession number. Sequence conservation is depicted by shading of purple color. Conserved cysteines residues are highlighted in green, putative glycosylation sites in SschwAg5r sequence are highlighted in blue. Lines below the alignment indicate conserved cysteines residues by “$”, glycosylation by “N” for N-glycosylation and by “O” for O-glycosylation and consensus sequence. Alignment was made by MAFFT with L-INS-i method and visualized in Jalview.
(PDF)

S4 Fig Multiple sequence alignment of sand flies’ lufaxin proteins. Multiple sequence alignment of *S*. *schwetzi* lufaxin proteins with chosen sand flies’ lufaxin proteins. Name of sequence include sand fly species shortcut (P.tob–*P*. *tobbi*, P.ser–*P*. *sergenti*, P.per–*P*. *perniciosus*, P.ari–*P*. *ariasi*, P.ara–*P*. *arabicus*, P.pap–*P*. *papatasi*, P.dub–*P*. *duboscqi*, P.arg–*P*. *argentipes*, L.lon–*L*. *longipalpis*, L.int–*L*. *intermedia*, L.aya–*L*. *ayacuchensis*, L.olm–*L*. *olmeca*, L.nei–*L*. *neivai*) and GenBank accession number. Sequence conservation is depicted by shading of purple color. Conserved cysteines residues are highlighted in green, putative glycosylation sites in SschwLuf sequences are highlighted in blue. Lines below the alignment indicate conserved cysteines residues by “$”, glycosylation by “N” for N-glycosylation and by “O” for O-glycosylation and consensus sequence. Alignment was made by MAFFT with L-INS-i method and visualized in Jalview.
(PDF)

S5 Fig Multiple sequence alignment of sand flies’ D7-related proteins. Multiple sequence alignment of *S*. *schwetzi* D7-related proteins with chosen sand flies’ D7-related proteins. Name of sequence include sand fly species shortcut (P.tob–*P*. *tobbi*, P.ser–*P*. *sergenti*, P.per–*P*. *perniciosus*, P.ori–*P*. *orientalis*, P.ari–*P*. *ariasi*, P.ara–*P*. *arabicus*, P.pap–*P*. *papatasi*, P.dub–*P*. *duboscqi*, P.arg–*P*. *argentipes*, L.lon–*L*. *longipalpis*, L.int–*L*. *intermedia*, L.aya–*L*. *ayacuchensis*, L.olm–*L*. *olmeca*, L.nei–*L*. *neivai*) and GenBank accession number. Sequence conservation is depicted by shading of purple color. Conserved cysteines residues are highlighted in green, putative glycosylation sites in SschwD7 sequences are highlighted in blue. Lines below the alignment indicate conserved cysteines residues by “$”, glycosylation by “N” for N-glycosylation and by “O” for O-glycosylation and consensus sequence. Alignment was made by MAFFT with L-INS-i method and visualized in Jalview.
(PDF)

S6 Fig Multiple sequence alignment of *S*. *schwetzi* PpSP15-like proteins. Multiple sequence alignment of *S*. *schwetzi* PpSP15-like proteins with two chosen *P*. *papatasi* SP15-like proteins. Name of sequence include sand fly species shortcut (P.pap–*P*. *papatasi*) and GenBank accession number. Sequence conservation is depicted by shading of purple color. Conserved cysteines residues are highlighted in green, putative glycosylation sites in SschwSP15 sequences are highlighted in blue. Lines below the alignment indicate conserved cysteines residues by “$” and for SschwSP15_2 the duplication of cysteines motive by “^”, glycosylation by “O” for O-glycosylation and consensus sequence. Alignment was made by MAFFT with L-INS-i method and visualized in Jalview.
(PDF)

S7 Fig Multiple sequence alignment of sand flies’ yellow-related proteins. Multiple sequence alignment of *S*. *schwetzi* YRPs with chosen sand flies’ YRPs. Name of sequence include sand fly species shortcut (P.tob–*P*. *tobbi*, P.ser–*P*. *sergenti*, P.per–*P*. *perniciosus*, P.ori–*P*. *orientalis*, P.ari–*P*. *ariasi*, P.ara–*P*. *arabicus*, P.pap–*P*. *papatasi*, P.dub–*P*. *duboscqi*, P.arg–*P*. *argentipes*, L.lon–*L*. *longipalpis*, L.int–*L*. *intermedia*, L.aya–*L*. *ayacuchensis*, L.olm–*L*. *olmeca*, L.nei–*L*. *neivai*) and GenBank accession number. Sequence conservation is depicted by shading of purple color. Conserved cysteines residues are highlighted in green, putative glycosylation sites in SschwYRPs sequences are highlighted in blue, putative amine binding residues are highlighted in orange. Lines below the alignment indicate amine binding site by “A”, conserved cysteines residues by “$”, glycosylation by “N” for N-glycosylation and by “O” for O-glycosylation and consensus sequence. Alignment was made by MAFFT with LINS-i method and visualized in Jalview.
(PDF)

S8 Fig Multiple sequence alignment of sand flies’ apyrases. Multiple sequence alignment of *S*. *schwetzi* apyrases with chosen sand flies’ apyrases. Name of sequence include sand fly species shortcut (P.tob–*P*. *tobbi*, P.ser–*P*. *sergenti*, P.per–*P*. *perniciosus*, P.ori–*P*. *orientalis*, P.ari–*P*. *ariasi*, P.ara–*P*. *arabicus*, P.pap–*P*. *papatasi*, P.dub–*P*. *duboscqi*, P.arg–*P*. *argentipes*, L.lon–*L*. *longipalpis*, L.int–*L*. *intermedia*, L.aya–*L*. *ayacuchensis*, L.olm–*L*. *olmeca*, L.nei–*L*. *neivai*) and GenBank accession number. Sequence conservation is depicted by shading of purple color. Active sites of enzyme are highlighted in orange, putative glycosylation sites in SschwApys sequence are highlighted in blue. Lines below the alignment indicate active site of enzyme by “A”, metal binding site by “&”, substrate binding site by “B”, glycosylation by “N” for N-glycosylation and “O” for O-glycosylation and consensus sequence. Alignment was made by MAFFT with L-INS-i method and visualized in Jalview.
(PDF)

S9 Fig Multiple sequence alignment of sand flies’ hyaluronidases. Multiple sequence alignment of *S*. *schwetzi* hyaluronidase with sand flies’ hyaluronidases. Name of sequence include sand fly species shortcut (P.tob–*P*. *tobbi*, P.per–*P*. *perniciosus*, P.ori–*P*. *orientalis*, P.ara–*P*. *arabicus*, L.lon–*L*. *longipalpis*, L.int–*L*. *intermedia*, L.olm–*L*. *olmeca*, L.nei–*L*. *neivai*) and GenBank accession number. Sequence conservation is depicted by shading of purple color. Active sites of enzyme are highlighted in orange, putative glycosylation sites in SschwHya1 sequence are highlighted in blue. Lines below the alignment indicate active site of enzyme by “A”, glycosylation by “N” for N-glycosylation and “O” for O-glycosylation and consensus sequence. Alignment was made by MAFFT with L-INS-i method and visualized in Jalview.
(PDF)

S10 Fig Multiple sequence alignment of sand flies’ 5’-nucleotidases. Multiple sequence alignment of *S*. *schwetzi* 5’-nucleotidase with other sand flies’ 5’-nucleotidases. Name of sequence include sand fly species shortcut (L.lon–*L*. *longipalpis*, L.nei–*L*. *neivai*) and GenBank accession number. Sequence conservation is depicted by shading of purple color. Active sites of enzyme are highlighted in orange, putative glycosylation sites in Sschw5nuc1 sequence are highlighted in blue. Lines below the alignment indicate active site of enzyme by “A”, metal binding site by “&”, glycosylation by “N” for N-glycosylation and consensus sequence. Alignment was made by MAFFT with L-INS-i method and visualized in Jalview.
(PDF)

S11 Fig Multiple sequence alignment of sand flies’ adenosine deaminases. Multiple sequence alignment of *S*. *schwetzi* and other sand flies’ adenosine deaminases. Name of sequence include sand fly species shortcut (P.per–*P*. *perniciosus*, P.dub–*P*. *duboscqi*, L.lon–*L*. *longipalpis*) and GenBank accession number. Sequence conservation is depicted by shading of purple color. Active sites of enzyme are highlighted in orange, putative glycosylation sites in SschwADA1 sequence are highlighted in blue. Lines below the alignment indicate active site of enzyme by “A”, glycosylation by “O” for O-glycosylation and consensus sequence. Alignment was made by MAFFT with L-INS-i method and visualized in Jalview.
(PDF)

S12 Fig Multiple sequence alignment of sand flies’ amylases. Multiple sequence alignment of *S*. *schwetzi* amylases and other sand flies’ amylases. Name of sequence include sand fly species shortcut (P.ara–*P*. *arabicus*, P.pap–*P*. *papatasi*, L.lon–*L*. *longipalpis*, L.nei–*L*. *neivai*) and GenBank accession number or UniProtKB accession number. Sequence conservation is depicted by shading of purple color. Active sites of enzyme are highlighted in orange, putative glycosylation sites in SschwAmy sequences are highlighted in blue. Lines below the alignment indicate active site of enzyme by “A”, metal binding site by “&”, glycosylation by “N” for N-glycosylation and “O” for O-glycosylation and consensus sequence. For easier visualization two parts of sequence *L*. *longipalpis* (A0A1B0CMM1) were hidden (highlighted by blue vertical lines with arrows, number of hidden aa is displayed below the alignment). Alignment was made by MAFFT with L-INS-i method and visualized in Jalview.
(PDF)

S13 Fig Multiple sequence alignment of sand flies’ endonucleases. Multiple sequence alignment of *S*. *schwetzi* and other sand flies endonucleases. Name of sequence include sand fly species shortcut (P.per–*P*. *perniciosus*, P.ori–*P*. *orientalis*, P.ari–*P*. *ariasi*, P.ara–*P*. *arabicus*, P.arg–*P*. *argentipes*, L.lon–*L*. *longipalpis*, L.int–*L*. *intermedia*, L.olm–*L*. *olmeca*, L.nei–*L*. *neivai*) and GenBank accession number or UniProtKB accession number. Sequence conservation is depicted by shading of purple color. Active sites of enzyme are highlighted in orange, putative glycosylation sites in SschwEnuc1 sequence are highlighted in blue. Lines below the alignment indicate active site of enzyme by “A”, metal binding site by “&”, glycosylation by “O” for O-glycosylation and consensus sequence. Alignment was made by MAFFT with L-INS-i method and visualized in Jalview.
(PDF)

S14 Fig Multiple sequence alignment of sand flies’ phospholipases A2. Multiple sequence alignment of *S*. *schwetzi* phospholipase A2 with other sand flies’ phospholipases A2. Name of sequence include sand fly species shortcut (P.per–*P*. *perniciosus*, P.ori–*P*. *orientalis*, P.ari–*P*. *ariasi*, P.ara–*P*. *arabicus*) and GenBank accession number. Sequence conservation is depicted by shading of purple color. Active sites of enzyme are highlighted in orange, putative glycosylation sites in SschwPLA2_1 sequence are highlighted in blue. Lines below the alignment indicate active site of enzyme by “A”, metal binding site by “&”, glycosylation by “N” for N-glycosylation and “O” for O-glycosylation and consensus sequence. Alignment was made by MAFFT with L-INS-i method and visualized in Jalview.
(PDF)

S15 Fig Multiple sequence alignment of sand flies’ pyrophosphatases. Multiple sequence alignment of *S*. *schwetzi* pyrophosphatase with other sand flies pyrophosphatases. Name of sequence include sand fly species shortcut (P.per–*P*. *perniciosus*, P.ori–*P*. *orientalis*, P.ara–*P*. *arabicus*, P.dub–*P*. *duboscqi*, P.arg–*P*. *argentipes*) and GenBank accession number. Sequence conservation is depicted by shading of purple color. Active sites of enzyme are highlighted in orange, putative glycosylation sites in SschwPP1 sequence are highlighted in blue. Lines below the alignment indicate active site of enzyme by “A”, metal binding site by “&”, glycosylation by “N” for N-glycosylation and “O” for O-glycosylation and consensus sequence. Alignment was made by MAFFT with L-INS-i method and visualized in Jalview.
(PDF)

S16 Fig Multiple sequence alignment of sand flies’ 71 kDa-like proteins. Multiple sequence alignment of *S*. *schwetzi* 71 kDa-like protein with other sand flies 71 kDa-like proteins. Name of sequence include sand fly species shortcut (L.lon–*L*. *longipalpis*, L.aya–*L*. *ayacuchensis*, L.olm–*L*. *olmeca*) and GenBank accession number or UniProtKB accession number. Sequence conservation is depicted by shading of purple color. Active sites of enzyme are highlighted in orange, putative glycosylation sites in Sschw71kDa1 sequence are highlighted in blue. Lines below the alignment indicate active site of enzyme by “A”, metal binding site by “&”, glycosylation by “N” for N-glycosylation, “O” for O-glycosylation, “C” for C-glycosylation and consensus sequence. Alignment was made by MAFFT with L-INS-i method and visualized in Jalview.
(PDF)

S17 Fig RNA-seq transcriptome analysis of *S*. *schwetzi* salivary glands. (PDF)

S18 Fig Proteome analysis of *S*. *schwetzi* salivary glands. (PDF)

S19 Fig Comparison of ATPase and ADPase activities and their pH optima in two *S*. *schwetzi* lineages. Comparison of ATPase and ADPase activities and their pH optima in two *S*. *schwetzi* lineages maintained on different blood-meal sources, geckos (S-G) and mice (S-M). Results represent the mean of five independent measurements.
(PDF)

S1 Table PCR and RT-qPCR primer sequences and reaction conditions. (XLSX)

S2 Table The annotation of *S*. *schwetzi* salivary proteins. (XLSX)

S3 Table Differential gene expression analysis (RNA-seq) of *S*. *schwetzi* transcripts. (XLSX)

S4 Table *S*. *schwetzi* salivary gland proteome analysis. (XLSX)
