# Supplementary material for: Sergentomyia schwetzi: Salivary gland transcriptome, proteome and enzymatic activities in two lineages adapted to different blood sources
Source: PLoS One. 2020 Mar 24;15(3):e0230537. doi: 10.1371/journal.pone.0230537 (PMC7092997; doi:10.1371/journal.pone.0230537)
Supplement: S1 Fig — (PDF) [file pone.0230537.s001.pdf]

Raw SDS-PAGE gels visualized on figure 12

A) protein separation on 10% PAGE gel

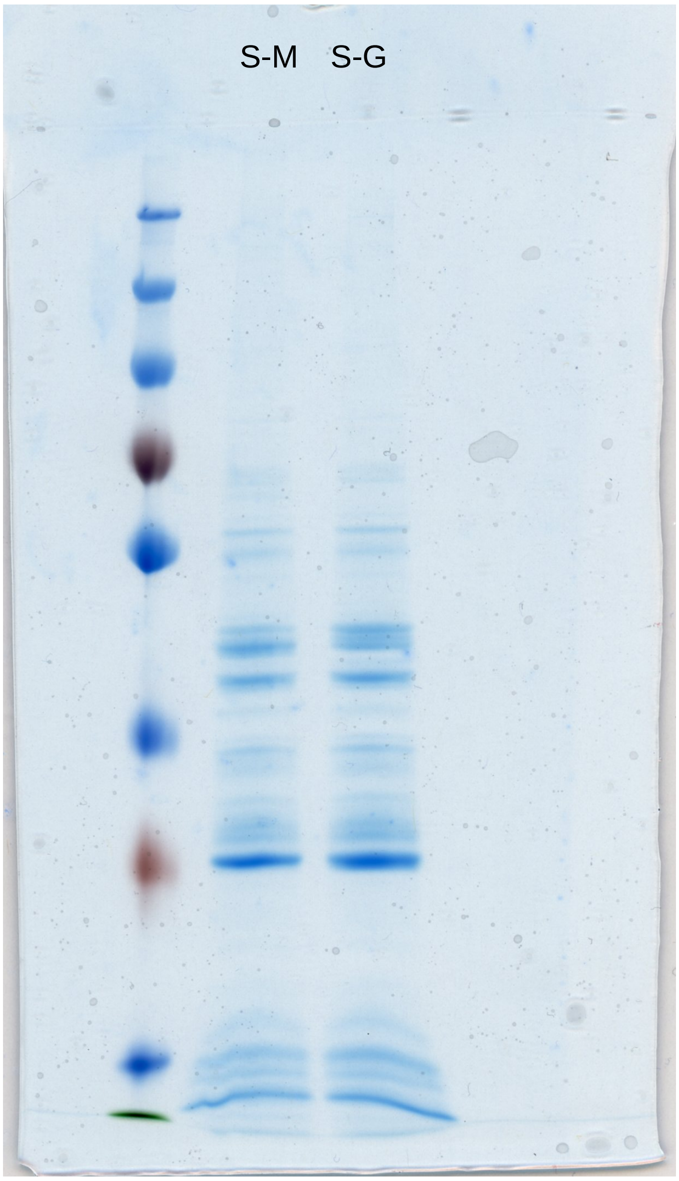

B) protein separation on 15% PAGE gel

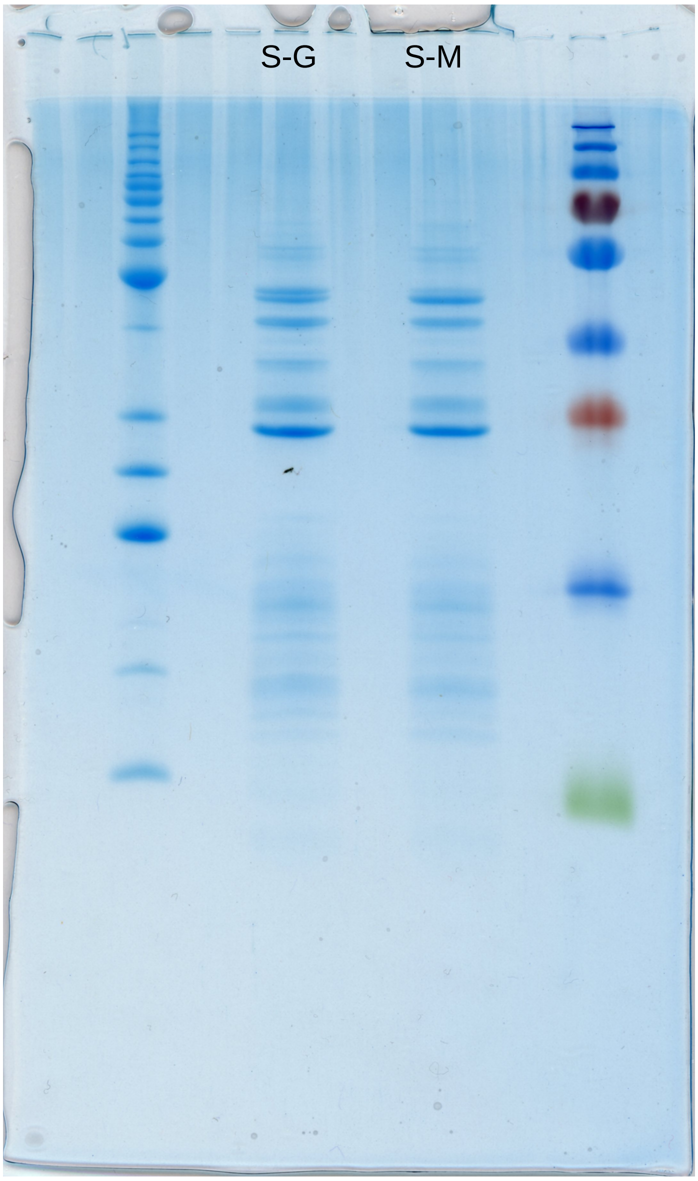

Raw SDS-PAGE zymography gels visualized on figure 13

A) Salivary gland homogenate tested on SDS PAGE zymography gel containing 10% polyacrylamide with 0.002% HA, tested under non-reducing conditions

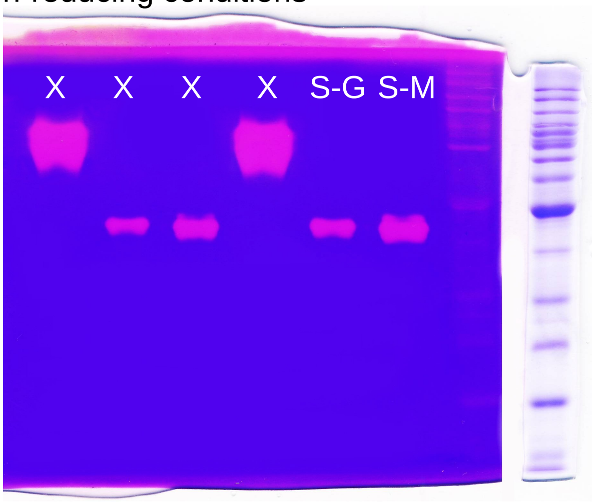

B) Salivary gland homogenate tested on SDS PAGE zymography gel containing 10% polyacrylamide with 0.002% HA, tested under reducing conditions

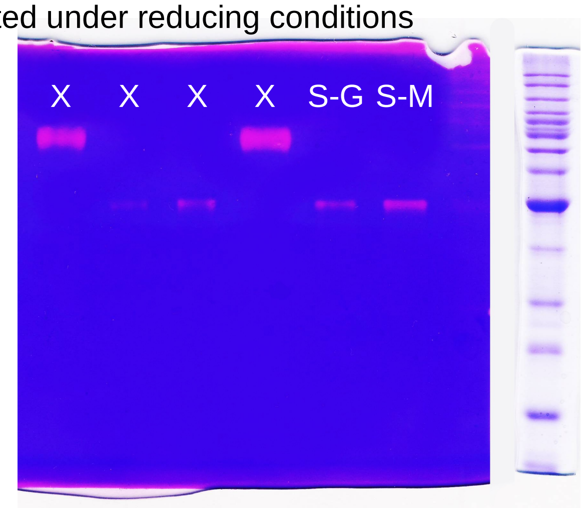

First three lines on both gels: concentration 0.5 salivary gland per line; second three lines: concentration 1 salivary gland per line, which was used for publication; samples order: positive control *Phlebotomus papatasi*, *Sergentomyia schwetzi* gecko lineage, *S. schwetzi* mouse lineage
