# Supplementary material for: Sergentomyia schwetzi: Salivary gland transcriptome, proteome and enzymatic activities in two lineages adapted to different blood sources
Source: PLoS One. 2020 Mar 24;15(3):e0230537. doi: 10.1371/journal.pone.0230537 (PMC7092997; doi:10.1371/journal.pone.0230537)
Supplement: S2 Fig — The percentage and distribution of top-level GO-terms are portrayed in the three categories: cellular component, molecular function and biological process. Transcripts annotated from whole transcriptome sequences dataset are indicated by blue bar (7,749 sequences) and transcripts annotated from arthropod sequences subset are in yellow (5,937 sequences). (PDF) [file pone.0230537.s002.pdf]

S2 Fig. Functional gene ontology (GO) classification of the *S. schwetzi* salivary gland transcriptome

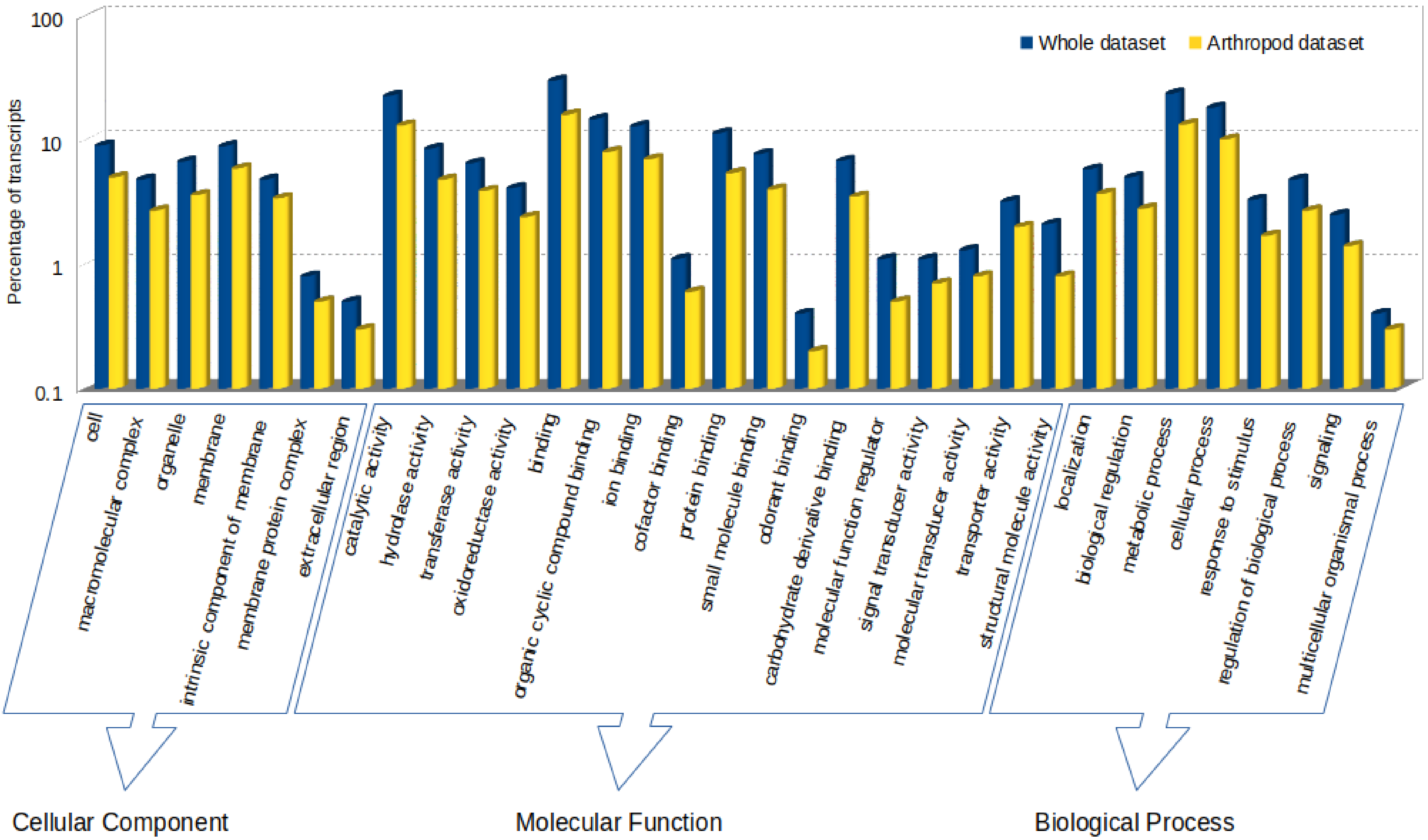

Functional gene ontology (GO) classification of the *S. schwetzi* salivary gland transcriptome. The percentage and distribution of top-level GO-terms are portrayed in the three categories: cellular component, molecular function and biological process. Transcripts annotated from whole transcriptome sequences dataset are indicated by blue bar (7,749 sequences) and transcripts annotated from arthropod sequences subset are in yellow (5,937 sequences).
