# Supplementary material for: Sergentomyia schwetzi: Salivary gland transcriptome, proteome and enzymatic activities in two lineages adapted to different blood sources
Source: PLoS One. 2020 Mar 24;15(3):e0230537. doi: 10.1371/journal.pone.0230537 (PMC7092997; doi:10.1371/journal.pone.0230537)
Supplement: S5 Fig — Multiple sequence alignment of S. schwetzi D7-related proteins with chosen sand flies’ D7-related proteins. Name of sequence include sand fly species shortcut (P.tob–P. tobbi, P.ser–P. sergenti, P.per–P. perniciosus, P.ori–P. orientalis, P.ari–P. ariasi, P.ara–P. arabicus, P.pap–P. papatasi, P.dub–P. duboscqi, P.arg–P. argentipes, L.lon–L. longipalpis, L.int–L. intermedia, L.aya–L. ayacuchensis, L.olm–L. olmeca, L.nei–L. neivai) and GenBank accession number. Sequence conservation is depicted by shading of purple color. Conserved cysteines residues are highlighted in green, putative glycosylation sites in SschwD7 sequences are highlighted in blue. Lines below the alignment indicate conserved cysteines residues by “$”, glycosylation by “N” for N-glycosylation and by “O” for O-glycosylation and consensus sequence. Alignment was made by MAFFT with L-INS-i method and visualized in Jalview. (PDF) [file pone.0230537.s005.pdf]

S5 Fig. Multiple sequence alignment of sand flies' D7-related proteins

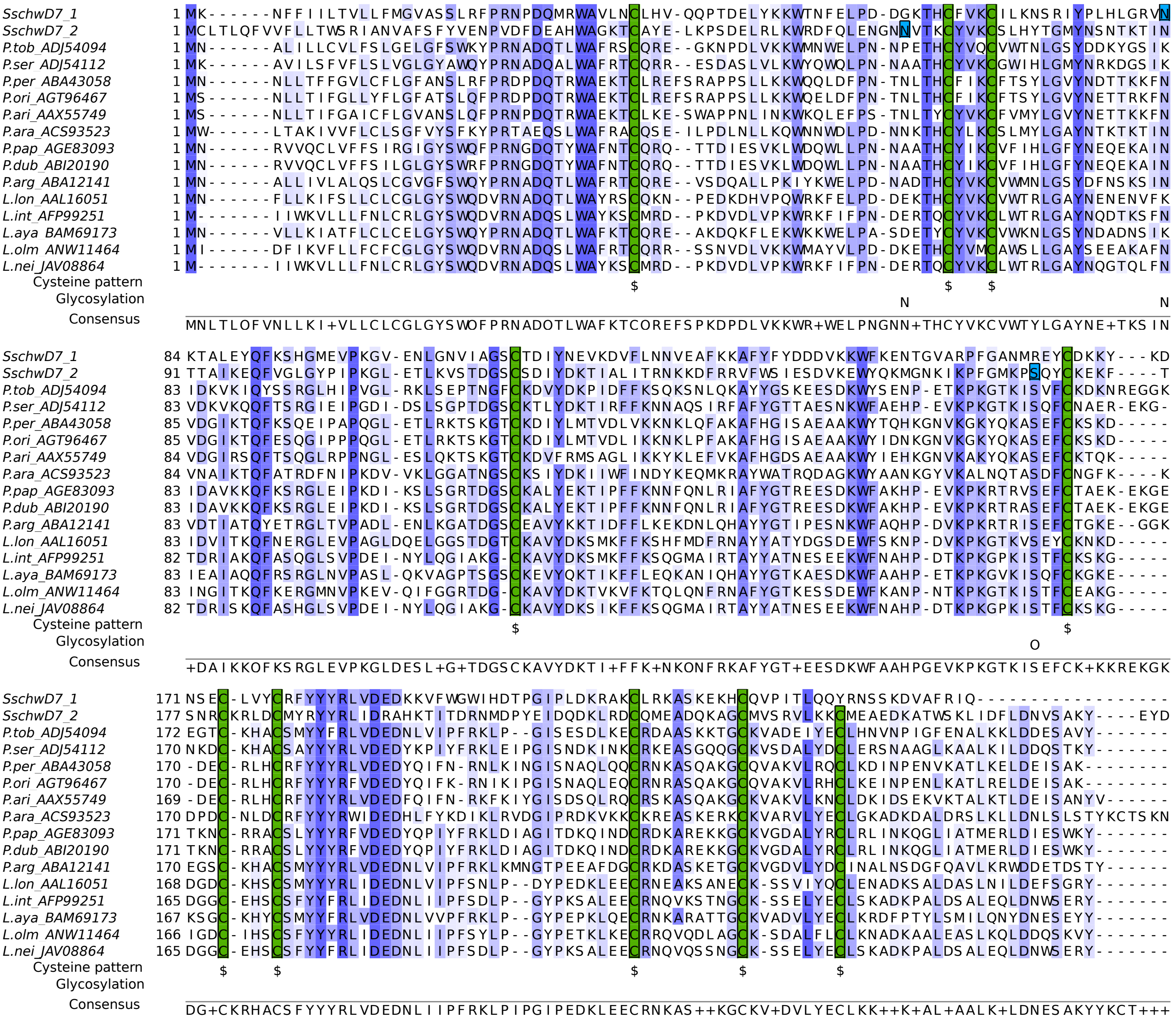

Multiple sequence alignment of *S. schwetzi* D7-related proteins with chosen sand flies' D7-related proteins. Name of sequence include sand fly species shortcut (P.tob – *P. tobbi*, P.ser – *P. sergenti*, P.per – *P. perniciosus*, P.ori – *P. orientalis*, P.ari – *P. ariasi*, P.ara – *P. arabicus*, P.pap – *P. papatasi*, P.dub – *P. duboscqi*, P.arg – *P. argentipes*, L.lon – *L. longipalpis*, L.int – *L. intermedia*, L.aya – *L. ayacuchensis*, L.olm – *L. olmeca*, L.nei – *L. neivai*) and GenBank accession number. Sequence conservation is depicted by shading of purple color. Conserved cysteines residues are highlighted in green, putative glycosylation sites in SschwD7 sequences are highlighted in blue. Lines below the alignment indicates conserved cysteines residues by "$", glycosylation by "N" for N-glycosylation and by "O" for O-glycosylation and consensus sequence. Alignment was made by MAFFT with L-INS-i method and visualized in Jalview.
