# Supplementary material for: Sergentomyia schwetzi: Salivary gland transcriptome, proteome and enzymatic activities in two lineages adapted to different blood sources
Source: PLoS One. 2020 Mar 24;15(3):e0230537. doi: 10.1371/journal.pone.0230537 (PMC7092997; doi:10.1371/journal.pone.0230537)
Supplement: S7 Fig — Multiple sequence alignment of S. schwetzi YRPs with chosen sand flies’ YRPs. Name of sequence include sand fly species shortcut (P.tob–P. tobbi, P.ser–P. sergenti, P.per–P. perniciosus, P.ori–P. orientalis, P.ari–P. ariasi, P.ara–P. arabicus, P.pap–P. papatasi, P.dub–P. duboscqi, P.arg–P. argentipes, L.lon–L. longipalpis, L.int–L. intermedia, L.aya–L. ayacuchensis, L.olm–L. olmeca, L.nei–L. neivai) and GenBank accession number. Sequence conservation is depicted by shading of purple color. Conserved cysteines residues are highlighted in green, putative glycosylation sites in SschwYRPs sequences are highlighted in blue, putative amine binding residues are highlighted in orange. Lines below the alignment indicate amine binding site by “A”, conserved cysteines residues by “$”, glycosylation by “N” for N-glycosylation and by “O” for O-glycosylation and consensus sequence. Alignment was made by MAFFT with LINS-i method and visualized in Jalview. (PDF) [file pone.0230537.s007.pdf]

S7 Fig. Multiple sequence alignment of sand flies' yellow-related proteins

```
SschwYRP1        1 M-NFFLWLFVCFSCILQEFNEASA--IQDRVKIV-YNWTVVEYAGKPEADYQSASIVPMGFAHYVDDKKVFIAIPRKEPG--ILETLTEIDDDMK-----N 90
SschwYRP2        1 M-KCLLCFVTILS-------VQQV--FAENVNDALFSWRNLLHE--NNGNSEAGASIPTAVAHYPQEKKLFIAIPRMFKSDDVQHTLTEVEVKNDG----N 85
SschwYRP3        1 M-KFLVWFPIFLI--FQE--FQSISSNGETVQIL-HHW--------IFGDSIIKQNIPDSIAHDVKNRKLFIAIPRVFES--NTMTLAEINDGS------A 79
P.tob_ADJ54079   1 M-KIFLCLIAVIS-------LQGV--LAYHIERE-YAWKNIIYEGIDPASYNVKNGIATGFAHDAASKKIFITVPRLNP---VPFTLTELDISKHP----G 83
P.ser_ADJ54123   1 M-KLILSVIALIS-------FQLV--FCDDVERA-FAWKNIIYENVKGTYKPEDIIPTGVTHDAKSKKLYFGIPRLHPN--IPYTLAELDTTKYNRSE-V 87
P.per_ABA43049   1 M-KIFLCLIAVVS-------LQGV--LAYDIERE-YAWKNISFEGIDPASYSVKNSIVTGFAHDADSKKIFITIPRLNP---VPITLTELDTTKHP----E 83
P.ori_AGT96427   1 M-KIFLCLIVVVS-------LQGV--LAYHIERE-YAWKNIIYEGINPASYNIENSIPTAFAHDATSKKIFITVPRINP---VPITLTEFDTTKHP----E 83
P.ari_AAX56360   1 M-KIFLCLFAAVS-------IQGA--LASQIERE-YAWKNIIYEGIDQGSYNIENSIPTAFAHDAASKKIFITIPRINQ---VPITLTEFDSIKYP----G 83
P.ara_ACS93501   1 M-KIFLCLFAVVS-------FHGA--FAYHVERE-YAWKNITYEGIDQASYNIENTIPTAFAHDAISKKIFITIPRRYPQ--VPITLTELDTSKHP----E 84
P.dub_ABI15941   1 M-KLILTVLAFLS-------LQVA--LSDDVGRL-YEWSKIDIVGVRPSVYDSSNIIPTGVAYDADSKMLFFGLPRKYSK--VPITVAQLSTRSYNSAE-R 87
P.pap_AGE83094   1 M-KLILCVLAFLS-------LQVA--LSDDVGRA-YEWSEIKLVGVRPNAYDSGNIVPTGVAYDAASKMLFFGIPRIYSR--VPITFAQLSTRSYNSAE-I 87
P.arg_ABA12136   1 M-KWFLFLLSTIF-------VQGI--LGYHVERE-YAWRNVTFEGVNPSSYNVLHSIPTGFAYDAETQKLFVAVPRRYPQ--VPHTLTEIERKKHP----E 84
L.lon_AAS05318   1 M-KVFFSIFTLVL-------FQGT--LGAD-TQG-YKWKQLLYNNVTPGSYNPDNMISTAFAYDAEGEKLFLAVPRKLPR--VPYTLAEVDTKNSLGVKGK 87
L.int_AFP99235   1 M-WCNLGLLVVIL-------FHGI--HGAYVEIG-YSWSNITFEGLDLKSYKPRHNVPTAFAYDATGHKKIFITIPRRLSG--VPYTVAELDTVKHPGYPID 88
L.aya_BAM69110   1 M-KLFLLVFTGIL-------FQGI--LGVEITQG-YKWRQILYGDVTPGTYNPDTNIPTAFAHDADGHKLFLTIPRKFPK--IPYTLAEVDTEKNPGVSGK 88
L.olm_ANW11467   1 MIMFCLIICTLLL-------LQGI--FGADVSKG-YMWNHISLEDIDKGAYDPSHILPTAFAHDANDHTMYLAIPRKVSD--IPYTLAEFDTTKNPGVEGN 89
L.nei_JAV07960   1 M-RILLVVFLSVF------YFKGI--LGVEIKEG-YKWKQILYEDVKPGTYNPDANIPTAFAHDPESHKLFLAFPRKFPY--IPYTIGEVDTKEHPGILGK 89
Amine binding site                                                             A
Cysteine pattern
Glycosylation                                  N                                                            N
Consensus          MIKIFLCLFAVVSCI+OEF+FOGISSLGYDVERELYAWKNI+YEGVDPGSYNPEN+IPTAFAHDADSKKLFI+IPRK+P+DDVP+TL+ELDT+KHPGVEGE
```

```
SschwYRP1       91 KSPRIKPFSG-KPTVPLVSIYQPVIDDCKRLWVLDAGSAEYTGH-DAP-TAQRPAII-AFDL-TNAEYKEIIRHEFT---SSEPLGFGGFAVDVVDPK--- 180
SschwYRP2       86 KSPKIKAFSG-NVKDGLRSVYQPVIDECKRLWIVD--------------DEKPTILTGYDL-TQKNYPEIARYSFPGDSTNADQSYGQFAVDVINPQ--- 166
SschwYRP3       80 RSANFIPFSG-KPTKPFISVYQVVVDECDRLWVLDAGSA-YKGR----YEKRNAAII-AFDLKTKKDYPEISRYEIPKEYVKEPHYFAKMAVDVVNPT--- 170
P.tob_ADJ54079  84 GSPPLSKFPG---GDKLISVYQPVIDECRRLWIVDVGQVEYKDD-EQKFPKQNAAII-AYDL-TKANYPEIDRYEIPSNVAGDSLGFGGFTVDVTNPK--- 175
P.ser_ADJ54123  88 RSPPLSKFNS-QSKEKFTSIYQPVIDDCRRLWVLDVGKVDYHKK-DNEYPTKNPEII-AFDL-NQPGNPEVHRYELTGDVAQTPLGFGGFAVDVINPK-C- 182
P.per_ABA43049  84 GSPPLSKFPG---SDKLISVYQPVIDECRRLWIVDAGQVEYKGD-EQKIPKKNAAII-AYDL-TKDNYPEIDRYEIPNNVAGNPLGFGGFAVDVTNPK--- 175
P.ori_AGT96427  84 GSPSLSKFPG---SDKIISVYQPVIDECRRLWIVDAGQVEYKED-EKKFKQNAAII-AYDL-TKDNYPEIDRYEIPSIVAGNPLGFGGFAVDVTNPK--- 175
P.ari_AAX56360  84 GSPPLSKFPG---SDNIISVYQPVIDECRRLWIVDAGQVEYKGD-EQKYPKKNPAII-AYDL-TKDNYPEIDRYEIPINIAGNPLGFGGFTVDVTNPK--- 175
P.ara_ACS93501  85 RSPPLSKFPG---SDDLINVYQPVIDECRRLWIVDAGEVEYKGD-EQKYPKRSAAII-AYDL-TKNNYPEIGRYEIPRKISGNPLGFGGFTIDVTNPT--- 176
P.dub_ABI15941  88 RDPPLDKFSG-KSKKPLTSVYQPVIDDCRRLWVLDVGIADVGQVEYKED-EKKFKQNAAII-AYDL-TKPDYPEIHRYELTGDAAKTPLGYGGFAVDVVNPKKCG 184
P.pap_AGE83094  88 PNPPLDKFSG-KSKQPLTSVYQPVIDDCRRLWVLDVGIVENEAE-RKTYPIKKPSLI-AFDL-TKSNYPEIHRYELTGEAGKNPLGYGGFAVDVVNPKRCS 184
P.arg_ABA12136  85 RSPPLSKFSG-KSSKDLISIYQPVIDECRRLWVVDVGMVDYKEG-QPKYRKQNPAII-AFDL-TKENYPEVDRYELPAEVVKNPLSFGCFAVDVINPK--- 178
L.lon_AAS05318  88 HSPLLNKFSGHKTGKELTSIYQPVIDDCRRLWVVDIGSVEYRSRGAKDYPSHRPAIV-AYDL-KQPNYPEVVRYYFPTRLVEKPTYFGGFAVDVANPK--- 183
L.int_AFP99235  89 RAPELDKFSG-KSSKDFVSAYQPVIDECRRLWILDVGQVEYSGDNSQKYSKQKPALI-VYDL-NKSNYPEVGRYELSDNVATSPTTFGGYAVDVTNNK--- 183
L.aya_BAM69110  89 RSPLLNRFSGHKSGKELTSVYQPVIDDCRRLWVVDVGSVEYRSRSAKDYPSHRPAIV-AYDL-KQPNYPEVVRYHFPVRLVEKPTYFGGFAVDVVNPT--- 184
L.olm_ANW11467  90 QEPLVHRFSGHKTGKELTSIYQPVIDECRRMWIVDVGVVEYTED-PKVHPIRNPSIV-AYDL-KTPGRPEVVRYDFPDISAEKPSFFGGFTVDVVNPS--- 184
L.nei_JAV07960  90 RAPLINRFSGHKSGKELTSVYQPVVDDCRRLWVVDVGSVEYRSRGAKAFPSHRPAIV-AYDL-KKPNFPEIIRYHFPVRLVEKPTFFGGFAVDVVS----- 183
Amine binding site                    AA
Cysteine pattern                             $
Glycosylation
Consensus          RSPPLSKFSGHKSSK+L+SVYOPVIDECRRLWVVDVG+VEYKGDGEKKYPK+NPAIITAYDLKTKPNYPEIDRYE+PGNVAGNPLGFGGFAVDVVNPK+C+
```

```
SschwYRP1      181 -NCNEVFVYMTNFKENSLYIYDHTKENSWTINLKSFQPDKETV-LEN-----FTYEVGIFGIALGKRNAQLNRSAYYISGSSTVFWSIETNLLRVKAPRNV 274
SschwYRP2      167 NGCDNTFVVIPSYSDNSLYVYDHKNKDSWTVTHSSFQPQKSTK---------STDDGIAAIALGERSGDGHRPAYYIISSSTKLWKVNTKDLKQKKAD-- 255
SschwYRP3      171 EGCDKTFIYIANHLECAMIVYNHKDKTSRRITHRSFQPKRQTE-ITR---ASLKYTSGLYSITLGQRDANGDRIAYYIPGSGTELFAINTKKIKNKEA-- 264
P.tob_ADJ54079 176 EGCAKTFVYIANFVDNALIVYDQEKKDSWKISHGSFKPEHDSI-LIH-DGAQHILKLGIFGITLGDLDAEGNRPAYFLGGSSTKLFKVNTKDLKKKNGQ-- 272
P.ser_ADJ54123 183 TKTDETYVYITNFIDNTLIVYDMKNKDAWKFKDDSFKPDLKNTAFTH-HGKEHPYTTGIFGIALGDRDKNGHRSAYYLAGSSTKLYNISTASLKEKDTH-- 280
P.per_ABA43049 176 EGCGKTFVYIANFEDNTLIVYDQEKKDSWKISHDSFKPEHESI-LTH-NGAQHILKLGIFGITLGDLDAEGNRQAYYLGGSSTKLFRVNTKDLKKKAGQ-- 272
P.ori_AGT96427 176 GGCGKTFIYIANFNDNTLIVYDQEKKDSWKISHGTFKPEHESI-LTH-NGAQHILKLGIFGITLGDLDAEGNRPAYYLGGSSTKLFRVNTKDLKKKDGQ-- 272
P.ari_AAX56360 176 EGCGKTFIYITNFEDNTLIVYDQEKKDSWKISHGSFKPEHESI-LIH-NGVDHILKLGIFGITLGDRDSEGNRPAYYLGGSSTKLFEVNTKALKKKEGE-- 272
P.ara_ACS93501 177 EGCGKTFIYITNFEENTLIVYDQEKKDSWKISHDSFKPEHDSV-LTH-NGKEHILKVGIFGITLGDRDPEGNRPAYYLGGSSTKLFEISTEYLKKKGAK-- 273
P.dub_ABI15941 185 KNDEKTYVYIANFVENSLIVYDKKSDAWVLKDDSFKPEGVST-YTH-NGKEHELKTGIFGIALGDRNKEGNRPAYYLAGSSTKLYRLDTKLLKKKGSK-- 281
P.pap_AGE83094 185 DKNEKTYIYIANFDENSLIVYDKKKGEAWSLKDDSFKPEGVTT-FTL-NGKEHKFKAGIFGIALGDRNKEGNRPAYYLAGSSTKLYRLDTKLLKKKGSK-- 281
P.arg_ABA12136 179 GGCSETFLYITNYEENTIVVYDKNKASWKVSHDSFKPEKDVNIVLD-GGKKYSYKVGIFGITLGDREATGNRMAYYLAGSSTKLYKVSTGALKKKGAR-- 276
L.lon_AAS05318 184 GDCSETFVYITNFLRGALFIYDHKKQDSWNVTHPTFKAERPTK-FDY-GGKEYEFKAGIFGITLGDRDSEGNRPAYYLAGSAIKVYSVNTKELKQKGGK-- 280
L.int_AFP99235 184 GDCSQTFVYITNFGENSLVVFDQKTKNSWKFTDKSFEPDKEST-FSHFNNQSDKYKVGLFGITLGDRDNAGNRPAYYIAGSSTKVYSVNTKQLKTKGTT-- 281
L.aya_BAM69110 185 GDCSETYVYITNFLSNALFIYDHKNQQSWNVTDATFKAERPST-FDH-QGKQYTYKAGLFGITLGDRDKDGNRPAYYLAGSSIKVYSVNTKELKQKNKK-- 281
L.olm_ANW11467 185 GDCSNTFVYITNFDTNALIIYDQKNNQFWKVSDPSFAPDVKST-FKH-AGKEYKYEFGLFAITLGDRDNEGNRPAYYLAGSSHKVYSVNTNQLKQKGAT-- 281
L.nei_JAV07960 184 KDCSDTFVYVTNFHRGTLFVYDHKNGQSWNVTHPTFRPERPTT-FNH-NDKEYTYNAGIFGITLGDRDSNGNRPAYYLAGSSIKVYSVNTKELKARGTT-- 280
Amine binding site                    A                                                      A
Cysteine pattern      $
Glycosylation                                          O           O      N
Consensus          GGCSKTFVYITNFEENTLIVYDOKKKDSWKISHDSFKPEHEST+FTHFNGKEHKYK+GIFGITLGDRDAEGNRPAYYLAGSSTKLYSVNTKDLKKKG+KNV
```

```
SschwYRP1      275 YTPKKIGDR-------------------GDDTDAIALAYDPDTEVIFFAEVKKRRITCWKTST-PFNTDNMGVVYES---DNLKFVTDISVDSAGDLWFLS 352
SschwYRP2      256 VNPETIGDR-------------------GRKTQGITMTYDPKTKVLFIGQLNNNGIFCWNTEK-KFE-DSKDTILVNPRDKESVNIIDTSVDNDGTLWVLS 335
SschwYRP3      265 FVPTRIGDRELPEDSPLRNKNLWALSYSTGLNDAITMAYDPLTKVLFVGEPNNRRIACWKTEG-EFTDENKDAIYSN--NDIYLFKDMSVDAHGTLWSLV 361
P.tob_ADJ54079 273 IEPIPLGDR-------------------GSNSEALALAYDPKTKVIFFIEYNSKRISCWNTQN-SLNHENIDVIYDN---PDFIFGTDISVDSESKLWFFS 350
P.ser_ADJ54123 281 LKPTLLGER-------------------GFKTEAIALAYDPKTKVIFFVESNSRQVSCWNTQM-ELKPENTGVIYSN---AYFVFGTDIMVDTDGILWFMA 358
P.per_ABA43049 273 IEFTPLGDR-------------------GSHSEALALAYDPKTKVIFFIEYNSKRISCWNTQK-SLNPDNIDVIYHS---PDFIFGTDISMDSESKLWFFS 350
P.ori_AGT96427 273 IEPTPLGDR-------------------GSHSEALALAYDPKTKVIFFTEYNSKRISCWNTQK-SLNHDSIDAIYDG---PDFIFGTDISVDSDSKLWFFS 350
P.ari_AAX56360 273 IEPITLGDR-------------------GPHSEALALAYDPKTKVIFFTEYNSKKISCWNIKK-PLIHDNMDKIYAS---PEFIFGTDISVDSESKLWFFS 350
P.ara_ACS93501 274 FDPVRLGDR-------------------GPKTEAIALAYDPKTKVIFFAEADTRQVSCWNTQK-PLNHKNTDVIYAS---AKFIYGTDISIDSESQLWFLS 351
P.dub_ABI15941 282 LVPKLIGDR-------------------GYKTEAIALAYDPETKVLFFAETDSRQVSCWNIKK-ELKPENVGVIYSS---AKLNFATDMMVDSKGFLWFMS 359
P.pap_AGE83094 282 LEPKLIGDR-------------------GFKTEAIALAYDPETKVLFFAEADSRQVSCWNIKH-DLKPENVGVIYAN---PNFNFGTDIMVDSKGFLWFLS 359
P.arg_ABA12136 277 FDPIRIGDR-------------------GPYTEAITLVYDPKTKVIFFAESITRQVSCWNTQT-PLDSNHTDVIYSD---ARFLFGTDISVDSNSTLWVMA 354
L.lon_AAS05318 281 LNPELLGNR-------------------GKYNDAIALAYDPKTKVIFFAEANTKQVSCWNTQKMPLRMKNTDVVYTS---SRFVFGTDISVDSKGGLWFMS 359
L.int_AFP99235 282 LTPKLHGDR-------------------GKHTDAIALAYDSVHKKVIFFAESDTRQVSCWHVDM-ELKPENTDVIYSY---ARFIFGTDISIDSENYLWFLS 359
L.aya_BAM69110 282 LNPELLGNR-------------------GKLNDAIALAYDPKTKVIFFAESNTRQVSCWNTQKMPLRMQHTDVIYSN---TRFVFGTDISVDSKGTLWFMS 360
L.olm_ANW11467 282 LSPVLLGER-------------------GEHSDAIALVYDSNTKVIFFAESNTGKITCWNSQKKPLKPENTHAIFNN---EDFIFMTDISVDSKGTLWFMA 360
L.nei_JAV07960 281 LNPQLLGNR-------------------GKLSDAIALAYDPKTKVIFFAEANTRQVSCWNTQKMPLRMANTDVIYTS---RHFIFGTDISVDSNSTLWFMS 359
Amine binding site                                          A                                                  A  AA
Cysteine pattern                                                               $
Glycosylation
Consensus          LEP+LLGDRELPEDSPLRNKNLWALSYSGK+TEAIALAYDPKTKVIFFAESN+RQVSCWNTOKMPL+PENTDVIYS+PRD+DFIFGTDISVDS+GTLWFMS
```

```
SschwYRP1      353 NADPPTGTKN------PDDDILLLKV-KASDAIQGTK-CENKA--------- 387
SschwYRP2      336 YLHFAEDK----PAAEDTPFFYISKV-TTSKAIAGTR-CQK----------- 370
SschwYRP3      362 YVLEPQEWYK--LSPKVIPRIQIWKA-DTSIAIGGTK-CDAGESMYY----- 404
P.tob_ADJ54079 351 NGHPPIENV---QLTHDKPHFRLISM-DTEKSIHGTK-CEVKS--------- 388
P.ser_ADJ54123 359 NGHPPIDEP---KLEFHKRQIRLMEV-PTHRAIRLQP-CEMKKNKKV----- 400
P.per_ABA43049 351 NGHPPIENV---QLTFDKPHFRLISM-DTKKSIHGTK-CEVKP--------- 388
P.ori_AGT96427 351 NGHPPIENL---QLTYDKPHIRLISM-DTEKSIHGTK-CEVK---------- 387
P.ari_AAX56360 351 NGHPPIENL---QLSSDKPHIHLISV-DTEKAIRGTK-CEVKA--------- 388
P.ara_ACS93501 352 NGHPPVENL---KLSFDKPHIRLMRV-DTEKSIRRTR-CEVKPIKKP----- 393
P.dub_ABI15941 360 NGQPPFDE----KMKYEDPHIRLMKV-KTKKAIKGEKRCQ----------G 395
P.pap_AGE83094 360 NGQPPIDE----KMAYDVPQIRLMKV-KTKRAIKGEK-CQ----------G 394
P.arg_ABA12136 355 NGHPPVDDPDIVNNEFYKPQIRLLYV-DTRKSIRRTR-CDVNGNKP----- 398
L.lon_AAS05318 360 NGFPPIRKSE--KFKYDFPRYRLMRIMDTQEAIAGTA-CDMNA-------- 399
L.int_AFP99235 360 NGYPPINDAE--KLKFDNRKIRLMRV-ATERVVKYAK-CNPNYKAPPSIPV 406
L.aya_BAM69110 361 NGFPPVKNSQ--KFKFDYPRYRLLNV-DTQAAIAGTN-CEIKPR------- 400
L.olm_ANW11467 361 NGMPPIDNSH--NFQYEKPRFRVMQV-DTKTAIAGTN-CE----------- 396
L.nei_JAV07960 360 NGFPPIRNAH--KFKYEDPRYRLFKV-DTRAAIAGTN-CEIKS-------- 398
Amine binding site A A
Cysteine pattern                                       $
Glycosylation
Consensus          NGHPPI+N++IVKLK+DKPHIRLMKVMDTEKAIAGTKRCEVK+NK+PSIPG
```

Multiple sequence alignment of *S. schwetzi* YRPs with chosen sand flies' YRPs. Name of sequence include sand fly species shortcut (P.tob – *P. tobbi*, P.ser – *P. sergenti*, P.per – *P. perniciosus*, P.ori – *P. orientalis*, P.ari – *P. ariasi*, P.ara – *P. arabicus*, P.pap – *P. papatasi*, P.dub – *P. duboscqi*, P.arg – *P. argentipes*, L.lon – *L. longipalpis*, L.int – *L. intermedia*, L.aya – *L. ayacuchensis*, L.olm – *L. olmeca*, L.nei – *L. neivai*) and GenBank accession number. Sequence conservation is depicted by shading of purple color. Conserved cysteines residues are highlighted in green, putative glycosylation sites in SschwYRPs sequences are highlighted in blue, putative amine binding residues are highlighted in orange. Lines below the alignment indicates amine binding site by "A", conserved cysteines residues by "$", glycosylation by "N" for N-glycosylation and by "O" for O-glycosylation and consensus sequence. Alignment was made by MAFFT with L-INS-i method and visualized in Jalview.
