# Supplementary material for: Sergentomyia schwetzi: Salivary gland transcriptome, proteome and enzymatic activities in two lineages adapted to different blood sources
Source: PLoS One. 2020 Mar 24;15(3):e0230537. doi: 10.1371/journal.pone.0230537 (PMC7092997; doi:10.1371/journal.pone.0230537)
Supplement: S9 Fig — Multiple sequence alignment of S. schwetzi hyaluronidase with sand flies’ hyaluronidases. Name of sequence include sand fly species shortcut (P.tob–P. tobbi, P.per–P. perniciosus, P.ori–P. orientalis, P.ara–P. arabicus, L.lon–L. longipalpis, L.int–L. intermedia, L.olm–L. olmeca, L.nei–L. neivai) and GenBank accession number. Sequence conservation is depicted by shading of purple color. Active sites of enzyme are highlighted in orange, putative glycosylation sites in SschwHya1 sequence are highlighted in blue. Lines below the alignment indicate active site of enzyme by “A”, glycosylation by “N” for N-glycosylation and “O” for O-glycosylation and consensus sequence. Alignment was made by MAFFT with L-INS-i method and visualized in Jalview. (PDF) [file pone.0230537.s009.pdf]

S9 Fig. Multiple sequence alignment of sand flies' hyaluronidases

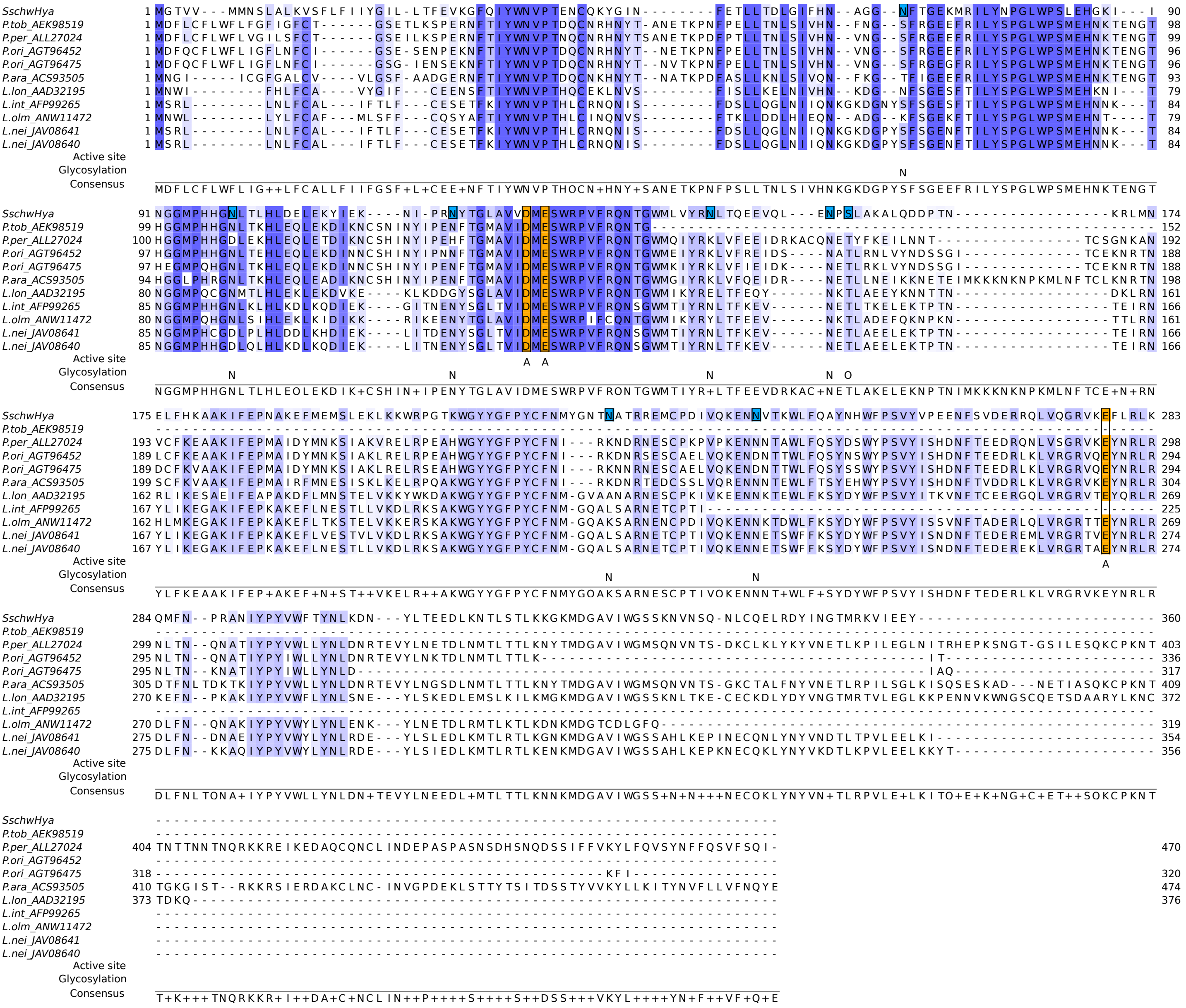

Multiple sequence alignment of *S. schwetzi* hyaluronidase with sand flies' hyaluronidases. Name of sequence include sand fly species shortcut (P.tob – *P. tobbi*, P.per – *P. perniciosus*, P.ori – *P. orientalis*, P.ara – *P. arabicus*, L.lon – *L. longipalpis*, L.int – *L. intermedia*, L.olm – *L. olmeca*, L.nei – *L. neivai*) and GenBank accession number. Sequence conservation is depicted by shading of purple color. Active sites of enzyme are highlighted in orange, putative glycosylation sites in SschwHya sequence are highlighted in blue. Lines below the alignment indicates active site of enzyme by "A", glycosylation by "N" for N-glycosylation and "O" for O-glycosylation and consensus sequence. Alignment was made by MAFFT with L-INS-i method and visualized in Jalview.
