# Supplementary material for: Sergentomyia schwetzi: Salivary gland transcriptome, proteome and enzymatic activities in two lineages adapted to different blood sources
Source: PLoS One. 2020 Mar 24;15(3):e0230537. doi: 10.1371/journal.pone.0230537 (PMC7092997; doi:10.1371/journal.pone.0230537)
Supplement: S10 Fig — Multiple sequence alignment of S. schwetzi 5’-nucleotidase with other sand flies’ 5’-nucleotidases. Name of sequence include sand fly species shortcut (L.lon–L. longipalpis, L.nei–L. neivai) and GenBank accession number. Sequence conservation is depicted by shading of purple color. Active sites of enzyme are highlighted in orange, putative glycosylation sites in Sschw5nuc1 sequence are highlighted in blue. Lines below the alignment indicate active site of enzyme by “A”, metal binding site by “&”, glycosylation by “N” for N-glycosylation and consensus sequence. Alignment was made by MAFFT with L-INS-i method and visualized in Jalview. (PDF) [file pone.0230537.s010.pdf]

S10 Fig. Multiple sequence alignment of sand flies’ 5’-nucleotidases

```
Sschw5nuc1            1 -MFFVKIFLFLLLAELGYL---AKSQRGNIEIVLLHTNDMHARFVETNRRGNVCHEKDKMAKQCYGGFSRISSKIKQFRKGN-NDVLFLNAGDTF 90
L.lon_AAD32190        1 MLFFLNFFVLVFSIELALLTASAAAEDGSYEIIILHTNDMHARFDQTNAGSNKCQEKDKIASKCYGGFARVSTMVKKFREENGSSVLFLNAGDTY 95
L.nei_JAV08428        1 -MFLTKFLVVLSTVGLAHL---APFDDGNYEIIILHNNDMHARFDQTNAQSNTCRQQEELDSKCYGGFARVSTIVNKFRKEN-DNVLFLNAGDTY 90
Active site                                                   A A                                                   A
Metal binding site                                            & &                                                   &
Glycosylation
Consensus               MMFF+KFFV+L+++ELA+LTASA+++DGNYEIIILHTNDMHARFDOTNA+SN+C+EKDK+ASKCYGGFARVST+VKKFRKENG++VLFLNAGDTY

Sschw5nuc1           91 QGTPAYTIFKHNITSEMMNLLKFDGISLGNHEFDDDIRGLAPYLKDLKVPVLAANIDASGEPSLT---NLKPSIIVTKNHLQIGIIGYLSTDTKF 182
L.lon_AAD32190       96 TGTPWFTLYKETIATEMMNILRPDAASLGNHEFDKGVEGLVPFLNGVTFPILTANLDTSQEPTMTNAKNLKRSMIFTVSGHRVGVIGYLTPDTKF 190
L.nei_JAV08428       91 TGTPWFTLYKDTISSEMMNILHPDAISLGNHEFDNGIEGLVPFLNKVTFPVLAANLDLSQEPTMASATSLKPSMVFTVAGHKIGIIGYLTPDTKF 185
Active site                                          AA  A
Metal binding site                                   &
Glycosylation                      N
Consensus               TGTPWFTLYK+TI+SEMMNIL+PDAISLGNHEFD+GIEGLVPFLN+VTFPVLAANLD+SOEPTMT+A+NLKPSMIFTV+GH+IGIIGYLTPDTKF

Sschw5nuc1          183 LSKPNKLKYTSEVSAINEEAKNL-RARGANVIIAVGHAGLTKDIEIAKNCPEVDLIVGGHSHTFLYTGTPPDIDTPKDNYPVVVTQPSGKKVPIV 276
L.lon_AAD32190      191 LSDVGKVNFIPEVEAINTEAQRLKKEENAEIIIVVGHSGLIKDREIAEKCPLVDIIVGGHSHTFLYTGSQPDREVPVDVYPVVVTQSSGKKVPIV 285
L.nei_JAV08428      186 LSAANKIEYIPEITAINAEAQKL-RSDNVDVIIALGHSGLEKDREIAAMCPEVDIVIGGHSHTFLYTADQPDREVPVDVYPVVVKQSSGKEVPVV 279
Active site             A                                   A                      A A
Metal binding site                                          &                      & &
Glycosylation
Consensus               LS++NK++YIPEV+AIN+EAO+LKR++NA+VIIAVGHSGL+KDREIA++CPEVDIIVGGHSHTFLYTG+OPDREVPVDVYPVVVTOSSGKKVPIV

Sschw5nuc1          277 QAYAYTKYLGYLKLKFNSTGSLLSWKGQPILLDHTVKQDPVILKVLKKKYLPAIDKYGKRVVGVSRVFLNGDYRTCRFQECNMGNLLTDAFLYGH 371
L.lon_AAD32190      286 QAYCFTKYLGYFKVTINGKGNVVGWTGQPILLNNNIPQDQEVLTAL-EKYRERVENYGNRVIGVSRVILNGGHTECRFHECNMGNLITDAFVYAN 379
L.nei_JAV08428      280 QAYAYTKYLGYLKIMVNSTGSIVDWDGQPILLNASIPQDSLVLAAL-QKYRAGVEEYGSRVVGVSRVILDGDSTTCRFRECNMGNLIADAFVHAN 373
Active site
Metal binding site
Glycosylation
Consensus               OAYAYTKYLGYLK+++NSTGS+V+W+GOPILLN++IPOD++VL+ALK+KYR++VE+YG+RVVGVSRVILNGD+TTCRF+ECNMGNLITDAFVYAN

Sschw5nuc1          372 VASNQDTNISKVWTDASIVLYQAGGIRAAIDVNITGGSITRHELDNVLPFSGDLSVSPVPGSTLRKALEHAVHRYTLKQGYGEFLQVSGLQVVYD 466
L.lon_AAD32190      380 VISTPMS--TNAWTDASVVLYQSGGIRAPIDPRTAAGSITRLELDNVLPFGNALYVVKVPGNVLRKALEHSVHRYSNTSGWGEFPQVSGLKIRFN 472
L.nei_JAV08428      374 AITTPLS--ETAWTDASVALYQSGGIRAPIDPRAAGGNITRLELDNVMPFGNTMYVVEVPGSVLREALEHSVHRYSKTTGWGEFLQVAGLQVVFD 466
Active site
Metal binding site
Glycosylation
Consensus               VISTP+SNI++AWTDASVVLYOSGGIRAPIDPR+AGGSITRLELDNVLPFGN+LYVV+VPGSVLRKALEHSVHRYS+T+GWGEFLOVSGLOVVFD

Sschw5nuc1          467 LNQDSGSRVKSVKVKCNECSVPRFEELDENRIYNVIINKFIKDGGDGFEMFKDLEVLKILPDIDIEMVDVYIGRLSPIYPAVEGRIIVKGKPKE- 560
L.lon_AAD32190      473 VNEEIGKRVKSVKVLCSNCSQPEYQPLRNKKTYNVIMDSFMKDGGDGYSMFKPLKIIKTLPLGDIETVEAYIEKMGPIFPAVEGRITVLGGLQK- 566
L.nei_JAV08428      467 FDKAIGERVESVNVLCSSCSQPKYSPLNNQQNYNVIMTNFLKDGGDGYDMFLPLKIIKTLPETDIQIVDTYIERMSPIFPAVEWRIIIKGDPPTT 561
Active site
Metal binding site
Glycosylation
Consensus               +N++IG+RVKSVKVLCS+CSOP+Y+PL+N+++YNVIM++F+KDGGDGY+MFKPLKIIKTLP++DIE+VD+YIERMSPIFPAVEGRIIVKG+P++T

Sschw5nuc1              ----------------------------------------
L.lon_AAD32190      567 ----------------------------------SDEDWH 572
L.nei_JAV08428      562 SPPTTKPPGDGGGSASSFALSGVLFITMLVIHVTSKIGSS 601
Active site
Metal binding site
Glycosylation
Consensus               SPPTTKPPGDGGGSASSFALSGVLFITMLVIHVTS+++++
```

Multiple sequence alignment of *S. schwetzi* 5’-nucleotidase with other sand flies’ 5’-nucleotidases. Name of sequence include sand fly species shortcut (L.lon – *L. longipalpis*, L.nei – *L. neivai*) and GenBank accession number. Sequence conservation is depicted by shading of purple color. Active sites of enzyme are highlighted in orange, putative glycosylation sites in Sschw5nuc sequence are highlighted in blue. Lines below the alignment indicates active site of enzyme by “A”, metal binding site by “&”, glycosylation by “N” for N-glycosylation and consensus sequence. Alignment was made by MAFFT with L-INS-i method and visualized in Jalview.
