# Supplementary material for: Sergentomyia schwetzi: Salivary gland transcriptome, proteome and enzymatic activities in two lineages adapted to different blood sources
Source: PLoS One. 2020 Mar 24;15(3):e0230537. doi: 10.1371/journal.pone.0230537 (PMC7092997; doi:10.1371/journal.pone.0230537)
Supplement: S11 Fig — Multiple sequence alignment of S. schwetzi and other sand flies’ adenosine deaminases. Name of sequence include sand fly species shortcut (P.per–P. perniciosus, P.dub–P. duboscqi, L.lon–L. longipalpis) and GenBank accession number. Sequence conservation is depicted by shading of purple color. Active sites of enzyme are highlighted in orange, putative glycosylation sites in SschwADA1 sequence are highlighted in blue. Lines below the alignment indicate active site of enzyme by “A”, glycosylation by “O” for O-glycosylation and consensus sequence. Alignment was made by MAFFT with L-INS-i method and visualized in Jalview. (PDF) [file pone.0230537.s011.pdf]

S11 Fig. Multiple sequence alignment of sand flies’ adenosine deaminases

```
SschwADA1         1 MFIKGIFLLLATSAVCHGVNLVDSFKALRLEREYAQYMRKRANFINDEEKQSLGSDLTLNDKEKIVNERLMELKKAEIDQGLSDPASFIP 90
P.per_ALL27025    1 MLLKVVVWLLATSAV-----LAFDWTEFRMERKYKDYLQKRSYYLAEEEDRSVGSDIELTAKEQIVNERLMALKRKELAEGLENPAGFIP 85
P.dub_ABI20162    1 MFPRLIVWLLAASAVH----AVLDISNIKPKRDYENFLQKYAEYADDEVDRSVGSDITLSLKEKFVNQYLMDLKTEELKAGLKNPSQFIP 86
L.lon_AAF78901    1 MFSQLVVWLLATSTVC----LAWD-NSWIMDMKYERYSQRRSYYLAEEEDRSVGSDIELTAKEQVVNERLMELKMTELKNGLQDPAGFIP 85
Active site
Glycosylation
Consensus           MF+KL+VWLLATSAVCHGVNL++D++++RMERKYE+YLOKR+YYLA+EEDRSVGSDI+LTAKE+IVNERLMELK++ELK+GL++PAGFIP

SschwADA1        91 WNHFFSVLDRINSSELFHIIQKMPKGGILHAHDTALCSTDYVVSLTYRDNLWQCEDTTTGALQFKFSREAPPSTDSCSWTLVKTEREAQG 180
P.per_ALL27025   86 WNHMFDVLNRINSSEIFDILRRMPKGGILHAHDTALCSTDYVISLTYEPNLWQCTDPETGALSFKFSREAPTNTETCQWTSVAAERTKLG 175
P.dub_ABI20162   87 SNHFFSVLDRINSSEIFKIIRRMPKGAILHAHDTALCSTDYVVSITYRDHLWQCADPKTGALQFRFSKESPKNTDTCQWTPVSEERKNQG 176
L.lon_AAF78901   86 WNHIFDVLYRINSSELFHIIQKMPKGGILHAHDTALCSTDYVISLTYEPNLWQCADPTTGAFQFLFSREAPTNTDTCTWTLVADERAKQG 175
Active site                                      A A  A
Glycosylation                                                                            O      O
Consensus           WNHFF+VLDRINSSE+FHII++MPKGGILHAHDTALCSTDYV+SLTY++NLWOCADPTTGALOFKFSREAPTNTDTCOWTLVA+ER+KOG

SschwADA1       181 EEEYNSKLRSQLSLYTTDPINHNRDVDSIWRQFMGLFGVNSGLLMYAPVWREYYYQFLTEMMEDGVQYLELRGTLPQLYDLDGKVYNEEE 270
P.per_ALL27025  176 EENYNSGLRSQLSLYTTDPINHNRDVDSIWRQFMGIFGVNDGLLSYAPIWKAYYKQFLKEMMEDGVQYLELRGTLPPLYDLDGKTYHEEE 265
P.dub_ABI20162  177 EEQYNSKLRSQLSLYNTDPINRSRDVDSIWNDFMGLFGVNFGLLTYAPVWKDYYKQFLKEMMEDGVQYLELRGTLPPLYDLDGKIYNEEQ 266
L.lon_AAF78901  176 EENYNSALRSQLSMYNTNPIMHNRDVDSIWRQFMGIFGVNGGLLTYAPVWKAYYLQFLKEMFADGVQYLELRTTLPPLYDLDGKTYNEVE 265
Active site
Glycosylation                      OO
Consensus           EENYNSKLRSOLSLY+TDPINHNRDVDSIWROFMG+FGVN+GLLTYAPVWKAYYKOFLKEMMEDGVOYLELRGTLPPLYDLDGKTYNEEE

SschwADA1       271 NLQIYYEETQRFMRENPTFIGAKFIYAPVRVVTEPDQPALMQKVVDLHNKYPNFMAGFDLVGQEDKGLPLIDFSKNLLSLPDSIDFFFHA 360
P.per_ALL27025  266 ILHIYQDATREFKQENPTFIGAKFIYAPVRVVDDAGLPALMDKVRELHEQYPNFMAGFDLVGQEDKGRPLIDFSKEILGLPDSINFFFHA 355
P.dub_ABI20162  267 VVEIYYNVTEEFKKENSTFIGAKFIYAPVRFVNATGIKTLTTTVKQLHERFPDFLAGFDLVGQEDKGGPLIGFSRELLELPESINFFFHS 356
L.lon_AAF78901  266 IMQIYYDATKEFKKQNPTFIGAKIIYAPVRVVDDAGIPALMAKVRELHEKFPDFMAGFDLVGQEDKGRPLIAFSREILKLPNSIDFYFHA 355
Active site                                                                  A                          A
Glycosylation
Consensus           ILOIYYDAT+EFKKENPTFIGAKFIYAPVRVVDDAGIPALM+KVRELHEK+P+FMAGFDLVGOEDKGRPLIDFS+E+L+LPDSI+FFFHA

SschwADA1       361 GETNWYGETDENLVDAVLLGTKRIGHGYAITKHPRVMREIRRKNIAVEVCPISNQVLRLVSDIRNHPMIYFMAN-DLPIVISSDDPSFWE 449
P.per_ALL27025  356 GETNWNGLTDDNLIDAVLLGTKRIGHGYAVLKHPRVLKQVKRDKIALEVCPVSNQVLRLVADMRNHPGAVLLANKEYPVVISSDDPSFWE 445
P.dub_ABI20162  357 GETNWNGMTDDNLIAAVTLGTKRIGHGYALFKHPRVLKQVKKDKIAIEVCPISNQVLRLVADMRNHPGSILLANKKYPMVISSDDPSFWE 446
L.lon_AAF78901  356 GETNWDGMTDDNLIDAVLLGTKRIGHGYAVLKHPRVLKEVKRNKIAIEVCPASNQVLRLVADYRNHPGSVLLANKEYPVVISSDDPSFWE 445
Active site          A                      A                                                          AA
Glycosylation
Consensus           GETNWNGMTDDNLIDAVLLGTKRIGHGYAVLKHPRVLK+VKRDKIAIEVCPISNOVLRLVADMRNHPGSVLLANKEYPVVISSDDPSFWE

SschwADA1       450 ATPLSHDFYIAFLGMTASYHDLRILKQLAMNSITYSSMNRSEKDRATKLWQAEWDKFINEMAASL----A                     515
P.per_ALL27025  446 AKPLTHDFYMAFLGLASERQDLRLLKQLAINSIKYSAMTPLEKMNAMRLWEAEWKKFIEELSA-------                     508
P.dub_ABI20162  447 ATPLSHDFYMAFMGLASYHQDLRMLKQLAINSLEYSSMTLEEKTNAMKLWEAEWEKFIKELETEVFSLLE                     516
L.lon_AAF78901  446 AKPLSHDFYMAFLGLASSRQDLRLLKQLAINSIKYSAMSPREKLQAMQMWEAEWKKFIDGFNA-------                     508
Active site
Glycosylation
Consensus           A+PLSHDFYMAFLGLASSRQDLRLLKQLAINSIKYS+MTP+EK+NAMKLWEAEWKKFI+EL+A++FSLL+
```

Multiple sequence alignment of *S. schwetzi* and other sand flies’ adenosine deaminases. Name of sequence include sand fly species shortcut (P.per – *P. perniciosus*, P.dub – *P. duboscqi*, L.lon – *L. longipalpis*) and GenBank accession number. Sequence conservation is depicted by shading of purple color. Active sites of enzyme are highlighted in orange, putative glycosylation sites in SschwADA1 sequence are highlighted in blue. Lines below the alignment indicates active site of enzyme by “A”, glycosylation by “O” for O-glycosylation and consensus sequence. Alignment was made by MAFFT with L-INS-i method and visualized in Jalview.
