# Supplementary material for: Sergentomyia schwetzi: Salivary gland transcriptome, proteome and enzymatic activities in two lineages adapted to different blood sources
Source: PLoS One. 2020 Mar 24;15(3):e0230537. doi: 10.1371/journal.pone.0230537 (PMC7092997; doi:10.1371/journal.pone.0230537)
Supplement: S12 Fig — Multiple sequence alignment of S. schwetzi amylases and other sand flies’ amylases. Name of sequence include sand fly species shortcut (P.ara–P. arabicus, P.pap–P. papatasi, L.lon–L. longipalpis, L.nei–L. neivai) and GenBank accession number or UniProtKB accession number. Sequence conservation is depicted by shading of purple color. Active sites of enzyme are highlighted in orange, putative glycosylation sites in SschwAmy sequences are highlighted in blue. Lines below the alignment indicate active site of enzyme by “A”, metal binding site by “&”, glycosylation by “N” for N-glycosylation and “O” for O-glycosylation and consensus sequence. For easier visualization two parts of sequence L. longipalpis (A0A1B0CMM1) were hidden (highlighted by blue vertical lines with arrows, number of hidden aa is displayed below the alignment). Alignment was made by MAFFT with L-INS-i method and visualized in Jalview. (PDF) [file pone.0230537.s012.pdf]

S12 Fig. Multiple sequence alignment of sand flies' amylases

```
SschwAmy1               1 -----------MKVFSALFVIFCLVTISQSQYNPHYIDNRNVIVHLFEWKWNDIADECVNFLGPNGFAGVQVSPVNENWISDERAWYERYQPMSYKLITRS 90
SschwAmy3               1 MSLVGASKSDKMKVLSTFLIICCLLGISKSQFNPNYVDNRTVMVQLFDWTFNDIADECVNFLGPYGFGGVQVSPITECWISEQGFWYERYSVISYKIFTRS 101
SschwAmy2               1 -----------MKLF-IVAL-LTFVSAAQGQFNPNFAAGRNVIVHLFEWKWEDIAAECERFLGPNGFAGVQVSPPNENVIVTNRPWWERYQPMSYRIETRS 88
P.ara_ACS93490          1 ------------------------------------------IVHLFEWKWNDIAKECQDFLGPYGFAGVQVSPVNENWVSDERAWYERYQPISYKLTTRS 59
P.pap_AGE83100          1 -----------MNILIALAVIVTSLSV-RAQFNPHFIDNRNVIVHLFEWKWNDIASECKNFLGPNGFAGVQVSPVNENWVSPERAWYERYQPISYKLTTRS 89
L.lon_AAD32192          1 -----------MKFLIGFGVVLLLVAV-AAQYDPHYLDKRTVMVHLFEWKWNDIAKECQDYLGPNGFGGVQVSPVNENWVSGERAWFERYQPISYKLTTRS 89
L.lon_A0A1B0CMM1        1 -----------MHFISGFGVVLILVAV-AAQYDPHYLDKRTVMVHLFEWKWNDIAKECQDYLGPNGFGGVQVSPVNENWVSGERAWFERYQPISYKLTTRS 525
L.nei_JAV08753            -----------------------------------------------------------------------------------------------------
L.nei_JAV08801          1 -----------MRLV-GVALVLGLCALSSGQHWPNFWGGRTGIVHLFEWKFSDVADECERFLAPKGFAGVQLSPVNENVIIPNRPWWERYQPISYVLTTRS 89
L.nei_JAV08800          1 -----------MRLV-GVALVLGLCALSSGQHWPNFWGGRTGIVHLFEWKFSDVADECERFLAPKGFAGVQLSPVNENVIIPNRPWWERYQPISYVLTTRS 89
Active site                                                                                                         AA  AA
Metal binding site
Glycosylation
                                        ^52 ^386
Consensus                 MSLVGASKSDKMKLLSG+AVVL+LVAVS++O+NP+++D+RTVIVHLFEWKWNDIADEC++FLGPNGFAGVOVSPVNENWISPERAWYERYOPISYKLTTRS

SschwAmy1              91 GTEEEFARMVQICRLQGVRIYVDVVFNHMASGNLGSKIYGTGGSEADPDTFYYPAVPYNKSDFHADCSISDYTDVYQVRNCQLASLRDLNQTIPYVREKII 191
SschwAmy3             102 GTEEEFARMVKVCQEQGVRIYVNVVFNHMTAN-LGPEIYGYGGSVAYPPDYYYPAVPYNKSHFHPECDITNFQNASEVRNCQLYTLRDLNQTIPYVREKII 201
SschwAmy2              89 GNEQQFANMVFRCNNVGVRIYPDIVINHMAASH--PVMIGTGGSTANVANRDYPAVPYSIHDFNPSCPITNYNDRYQVRNCELVGLPDLNQGVAWVRDRIV 187
P.ara_ACS93490         60 GTEQEFASMVETCRQRGVRIFVDVVFNHMASGPLGKTIVGTAGSKAHPDTFDYPAVPFDKCCFHPDCSIIDYKDVNQVRNCQLASLRDLNQTIPYVREKIL 160
P.pap_AGE83100         90 GTQEEFASMVETCRQSGVRIYVDVVFNHMASGSLDTILVGTAGSEAYPQVFDYPAVPFTKADFHPDCTITDYQDVYQIRNCQLSSLRDLNQTSPYVREKIL 190
L.lon_AAD32192         90 GNEDEFAAMVKTCRQNGVRIFVDVVVNHMASGALEDTIYGTGGSEAHPGPFDYPAVPYEKNDFHPDCSISDYQDVYQVRNCQLSSLRDLNQTIPYVREKIL 190
L.lon_A0A1B0CMM1      526 GTEDEFAAMVKTCRQNGVRIFVDIVVNHMASGALEDTIYGTGGSEAHPGPFDYPAVPYEKNDFHPDCSISDYQNVYQVRNCQLSSLRDLNQTIPYVREKIL 626
L.nei_JAV08753          1 ------------------RIYVDVVFNHMAAGE--GEVVGTGGSLVYPEERLYPHVPYGPEDFNPPCIIKDYQDVKQVRNCGLVSLPDLNQKSENVRRSVI 81
L.nei_JAV08801         90 GNENDFLDMSRRCNAVGIRIYVDVLLNHMSADN--DPAHGTAGNHAVTSQRSWPAVPYGPNDFNPRCEIHNWDDPWEIRQCELVGLHDLNQGTEHVRNMLV 188
L.nei_JAV08800         90 GNENDFLDMSRRCNAVGIRIYVDVLLNHMSADN--DPAHGTAGNHAVTSQRSWPAVPYGPNDFNPRCEIHNWDDPWEIRQCELVGLHDLNQGTEHVRNMLV 188
Active site                                       A  A                                                      AAAA
Metal binding site                                  &                                                            &
Glycosylation                                                      OO             N      O
                                                                                    O
Consensus                 GTEEEFA+MVKTCROVGVRIYVDVVFNHMA+GNLGDTIYGTGGSEA+P++FDYPAVPYGKNDFHPDCSI+DYODVYOVRNCOLVSLRDLNOTIPYVREKIL

SschwAmy1             192 DFLNHLVDLGVAGFRVDAAKHMDPSDLRYIYSNIKKLNTSFGFRPTDRAFIAQEVIDLGGEAITSSQYAPLGVVTEFKASNFLGEAFRGIIQLADLVRWGP 292
SschwAmy3             202 GLLDHLIDLGVAGFRIDAAKHMDPSDLGYIYSHTKNLNPLFGFKHTDTALFAQEVIDYGFEPITASEYSPIGVVTEFKACYFLGVAFRGKLKLAVLKTWGP 302
SschwAmy2             188 EYLDHLVALGVAGFRIDAAKHMWPGDLEIIYSRVRNLNTVHGFPGNSRPFFFQEVIDQGGEAVSSTEYIGFGVVTEFKFSAEIGRVFRGNDLLTHLNNWGE 288
P.ara_ACS93490        161 DFLNHLVYLGVAGFRVDAAKHMDPKDLKYIYSHIKNLNTNFGFKATDKAFIVQEVIDLGGEAITSQQYGTLGVVTEFKASDDLGKVFRGQVPMTELDKWGT 261
P.pap_AGE83100        191 GFLNQLVDLGVAGFRVDAAKHMDPQDLRYIYRTIKRLNKDSGFRATDKAFIVQEVIDLGGEAVTSREYTSFGVVTEFKASDELGKAFRGKYPLADLRHWGP 291
L.lon_AAD32192        191 DFLNHLVDLGVAGFRIDAAKHMDPKDLRYIYNHIKKLNKDAEFKAGDKAFIFQEVIDLGGEAVSSREYISLGVVTEFKASDDLGKLFRGQVALSTLERWGP 291
L.lon_A0A1B0CMM1      627 DFLNHLVDLGVAGFRIDAAKHMDPKDLRYIYNHIKKLNKDAE--AGDKAFIFQEVIDLGGEAVSSREYISLGVVTEFKASDDLGKLFRGQVALSTLERWGP 725
L.nei_JAV08753         82 EFLDRLIDYGVAGFRADACKHMWPEDVEFLFGNTKDLNKEFGFPKNARPFLYQEVIDLGQEPISKNEYTSIGVVTEFLFSAEIGNTFRNK-KLKDLMQWGS 181
L.nei_JAV08801        189 NFLDHLVDLGVAGFRVDAAKHVWPHDLEVIYNRVKNLNTDFGFAPGSRAFIYQEVIDLGGDPI-RYEYTPLGAVTEFKFSAEIGAAFRGYNQLRWLYNFGP 288
L.nei_JAV08800        189 NFLDHLVDLGVAGFRVDAAKHVWPHDLEVIYNRVKNLNTDFGFAPGSRAFIYQEVIDLGGDPI-RYEYTPLGAVTEFKFSAEIGGAFRGYNQLRWLYNFGP 288
Active site                             A AA AA                                A A    A
Metal binding site
Glycosylation
Consensus                 DFL+HLVDLGVAGFRVDAAKHMDPKDL+YIY+HIKNLNTDFGFKA+DRAFI+OEVIDLGGEAI+SREYT+LGVVTEFKAS+ELGKAFRG+VOLADL++WGP

SschwAmy1             293 MF---QMLPSNKALVFVENHDNERGHGAGGSNILTYKNEKIYQMAVVFNLAHNYGIPRIMSSYAFDDASQGPPHDSDYNILRPSPFSNGGTTCSNGWVCQH 390
SschwAmy3             303 SFSQLSMLPSNRAFVFVDNHDTER---QGGPDLISYKDGKIYIMAVIFNLAHIYGTPRIMSSYAFDNYDQGPPHDENNNIIGPGPFTNGETTCTNDWICQH 400
SschwAmy2             289 GW---GFLPSNRALVFVENHDNERGHGAGGDQILTYKQAKQYKMAVAFGLAHTYGIPRIMSSFAFTNTEVGPPMDGNQNIVSPS-INADG-TCGNGWVCQH 384
P.ara_ACS93490        262 RP---GLLSSYQALVFVENHDNERGHGAGGDNILTYKNGKIYTMAVVFNLAHSYGIPRVMSSYHFDDPSQGPPHDDYGNILSPE-FSSDGQSCSNGWVCQH 358
P.pap_AGE83100        292 LY---GLLPPNRALVFVENHDNERGHGAGGENILTYKNGKIYTMAVAFNLAHSYGIPRIMSSYEFNDPSQGPPHDDSNNILSPE-FSSNGESCTNGWVCQH 388
L.lon_AAD32192        292 QY---GLLPSNRALAFVENHDNERGHGAGGTNILTYKDGKIYTMAVVFNLAHSYGVPRMMSSYEFNDPSQGPPHDDNSNILTPE-FSADGNSCTNGWVCQH 388
L.lon_A0A1B0CMM1      726 QY---GLLPSNRALAFVENHDNERGHGAGGTNILTYKDGKIYTMAVVFNLAHSYGVPRMMSSYEFNDPSQGPPHDDNNNILTPE-FSADGNSCTNGWVCQH 822
L.nei_JAV08753        182 DK---RFLRSDRALVFVENHDNERGHGAGGKDILTYKDGKLYLLAVLFTISHPYGIPRIMSSYEFSNSDQGPPASANGDIKSPV-MNSRG-ICTNGWICQH 277
L.nei_JAV08801        289 HW---GFVPSDVALVFVDNHDNQRGHGAGGGNVLTYKQPRHYKMATAFTLAWNYGTVRLMSSFAFDHGDQGPPQDANENIISPS-INPDN-SCGNGWVCEH 384
L.nei_JAV08800        289 AW---GFVPSDVALVFVDNHDNQRGHGGGGGNVLTYKQPRHYKMATAFTLAWNYGTVRLMSSFAFDDGDQGPPQDANENIISPS-INPDN-SCGNGWVCEH 384
Active site                                  AA    A  a
Metal binding site
Glycosylation                                                                                                 O O
Consensus                 O+SOLG+LPSNRALVFVENHDNERGHGAGG+NILTYKDGKIYTMAV+FNLAHSYGIPRIMSSYAFDDPSOGPPHDDNNNILSP+PFS+DG+SCTNGWVCOH

SschwAmy1             391 RWRPIRNMVKFRNVVGIAPVTLWYDNGSNQIAFSRGVRGFVAFNN-DVVDFNAKIRTFLPSGTYCDIISGEKLNGGCTGKTLGV-RSGIVGVIIRADDDFG 489
SschwAmy3             401 RWRPIRNMVQFRNVVKEEPLTLWYDNGGNQIAFSRGGRGFVAFNN-EDFAFSSIVKTSLPTGIYCDVISGEKINGICTGKIIAV-MGGEVTVNIPANDEFG 499
SschwAmy2             385 RWRQIFNMVRFRNEVGTAALTNWWSNGSQQIAFARAGRGFIAFNN-QGSNMNVNLQTSLPGGTYCDVISGNVSGGSCTGKTVHVNGDGTANISIGAAEEDG 484
P.ara_ACS93490        359 RWLPIRNMVKFRNDVGTSSVVLWQDNGSNQIGFSRGNRGFVAFNK-DDVDLNKQFSTNLAPGFYCDVISGEKSGNSCTGKVLFV-KNSKVSVIIRADDPYG 457
P.pap_AGE83100        389 RWLAIRNMVKFRNVVRKTSVTLWYDNGSNQIAFSRGTRGFIAFNG-DKVDFNAKVRTYLRSGTYCDVISGEKNGNSCTGKTVNV-RRSEVAVIIEADDPYG 487
L.lon_AAD32192        389 RWRPMRNMVKFRNIVGRKSVRKWYDNGSNQIAFSRGNQGFVAFNL-DIVDFNQQVPTDLPDGVYCDVISGEKNGNECTGKVVIV-SKRKAAVILRADDNYG 487
L.lon_A0A1B0CMM1      823 RWRPMRNMVKFRNIVGRKPVRKWYDNGSNQIAFSRGNQGFVAFNM-DIVDFNQQVPTDLPDGVYCDVITGEKNGNECTGKVVIV-SKRKAAVILRADDDYG 921
L.nei_JAV08753        278 RWPGVVGMVQFRNAVAGRGILNWMDNGEQQIAFCRGDLGFVAFNGYTMANLNSTVKVCLPPGIYCDVITGDVTDAGCTGQEVLVNKSGYANIFIPGDSPTG 378
L.nei_JAV08801        385 RWRQIYNMIGFANQVRGTSVNDWWDNGNSQIAFCRGNQGFIAFNN-EQHNFSHTLQTCLPAGTYCDIISGENTGNGCSGVTITVNWDQTAHIFIPVDHYDG 484
L.nei_JAV08800        385 RWRQIYNMIGFANQVRGTSVNDWFDNGNSQIAFCRGDQGFIAFNN-EQHNFSHTLQTCLPAGTYCDIISGENTGNGCSGVTITVHWDQTAHIFIPVDHYDG 484
Active site
Metal binding site
Glycosylation                                      N
Consensus                 RWRPIRNMVKFRNVVGGTSVTLWYDNGSNOIAFSRGNRGFVAFNNYD+VDFN++VOTCLP+GTYCDVISGEK+GNGCTGKTV+VN+DG+AAVII+ADDPYG

SschwAmy1             490 VVAIHRDSKL--- 499
SschwAmy3             500 TIAIHTNSKL--- 509
SschwAmy2             485 VLAIHVGARL--- 494
P.ara_ACS93490        458 VVAIHTESKL--- 467
P.pap_AGE83100        488 VIAIHAESKL--- 497
L.lon_AAD32192        488 VLAIHSESKL--- 497
L.lon_A0A1B0CMM1      922 VLAIHSESKL--- 931
L.nei_JAV08753        379 VLAIHWGSVYLLD 391
L.nei_JAV08801        485 VIAIHQGPTSKVN 497
L.nei_JAV08800        485 VIAIHRGPTSRVN 497
Active site
Metal binding site
Glycosylation
Consensus                 V+AIH++SKL+VN
```

Multiple sequence alignment of *S. schwetzi* amylases and other sand flies' amylases. Name of sequence include sand fly species shortcut (P.ara – *P. arabicus*, P.pap – *P. papatasi*, L.lon – *L. longipalpis*, L.nei – *L. neivai*) and GenBank accession number or UniProtKB accession number. Sequence conservation is depicted by shading of purple color. Active sites of enzyme are highlighted in orange, putative glycosylation sites in SschwAmy sequences are highlighted in blue. Lines below the alignment indicates active site of enzyme by "A", metal binding site by "&", glycosylation by "N" for N-glycosylation and "O" for O-glycosylation and consensus sequence. For easier visualisation two parts of sequence *L. longipalpis* (A0A1B0CMM1) were hidden (highlighted by blue vertical lines with arrows, number of hidden aa is displayed below the alignment). Alignment was made by MAFFT with L-INS-i method and visualized in Jalview.
