# Supplementary material for: Sergentomyia schwetzi: Salivary gland transcriptome, proteome and enzymatic activities in two lineages adapted to different blood sources
Source: PLoS One. 2020 Mar 24;15(3):e0230537. doi: 10.1371/journal.pone.0230537 (PMC7092997; doi:10.1371/journal.pone.0230537)
Supplement: S15 Fig — Multiple sequence alignment of S. schwetzi pyrophosphatase with other sand flies pyrophosphatases. Name of sequence include sand fly species shortcut (P.per–P. perniciosus, P.ori–P. orientalis, P.ara–P. arabicus, P.dub–P. duboscqi, P.arg–P. argentipes) and GenBank accession number. Sequence conservation is depicted by shading of purple color. Active sites of enzyme are highlighted in orange, putative glycosylation sites in SschwPP1 sequence are highlighted in blue. Lines below the alignment indicate active site of enzyme by “A”, metal binding site by “&”, glycosylation by “N” for N-glycosylation and “O” for O-glycosylation and consensus sequence. Alignment was made by MAFFT with L-INS-i method and visualized in Jalview. (PDF) [file pone.0230537.s015.pdf]

S15 Fig. Multiple sequence alignment of sand flies' pyrophosphatases

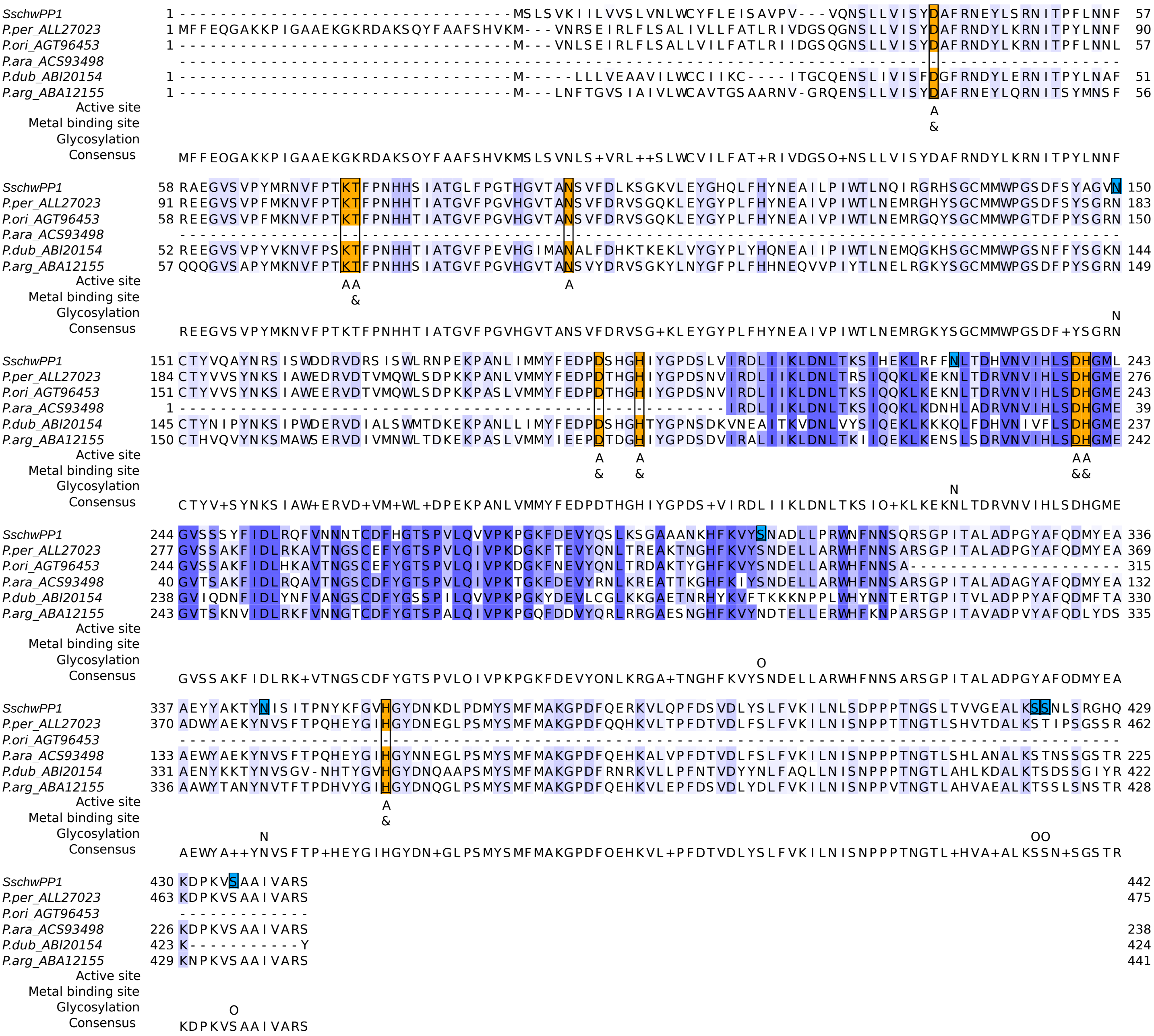

Multiple sequence alignment of *S. schwetzi* pyrophosphatase with other sand flies pyrophosphatases. Name of sequence include sand fly species shortcut (P.per – *P. perniciosus*, P.ori – *P. orientalis*, P.ara – *P. arabicus*, P.dub – *P. duboscqi*, P.arg – *P. argentipes*) and GenBank accession number. Sequence conservation is depicted by shading of purple color. Active sites of enzyme are highlighted in orange, putative glycosylation sites in SschwPP1 sequence are highlighted in blue. Lines below the alignment indicates active site of enzyme by "A", metal binding site by "&", glycosylation by "N" for N-glycosylation and "O" for O-glycosylation and consensus sequence. Alignment was made by MAFFT with L-INS-i method and visualized in Jalview.
