# Supplementary material for: Sergentomyia schwetzi: Salivary gland transcriptome, proteome and enzymatic activities in two lineages adapted to different blood sources
Source: PLoS One. 2020 Mar 24;15(3):e0230537. doi: 10.1371/journal.pone.0230537 (PMC7092997; doi:10.1371/journal.pone.0230537)
Supplement: S16 Fig — Multiple sequence alignment of S. schwetzi 71 kDa-like protein with other sand flies 71 kDa-like proteins. Name of sequence include sand fly species shortcut (L.lon–L. longipalpis, L.aya–L. ayacuchensis, L.olm–L. olmeca) and GenBank accession number or UniProtKB accession number. Sequence conservation is depicted by shading of purple color. Active sites of enzyme are highlighted in orange, putative glycosylation sites in Sschw71kDa1 sequence are highlighted in blue. Lines below the alignment indicate active site of enzyme by “A”, metal binding site by “&”, glycosylation by “N” for N-glycosylation, “O” for O-glycosylation, “C” for C-glycosylation and consensus sequence. Alignment was made by MAFFT with L-INS-i method and visualized in Jalview. (PDF) [file pone.0230537.s016.pdf]

S16 Fig. Multiple sequence alignment of sand flies' 71 kDa-like proteins

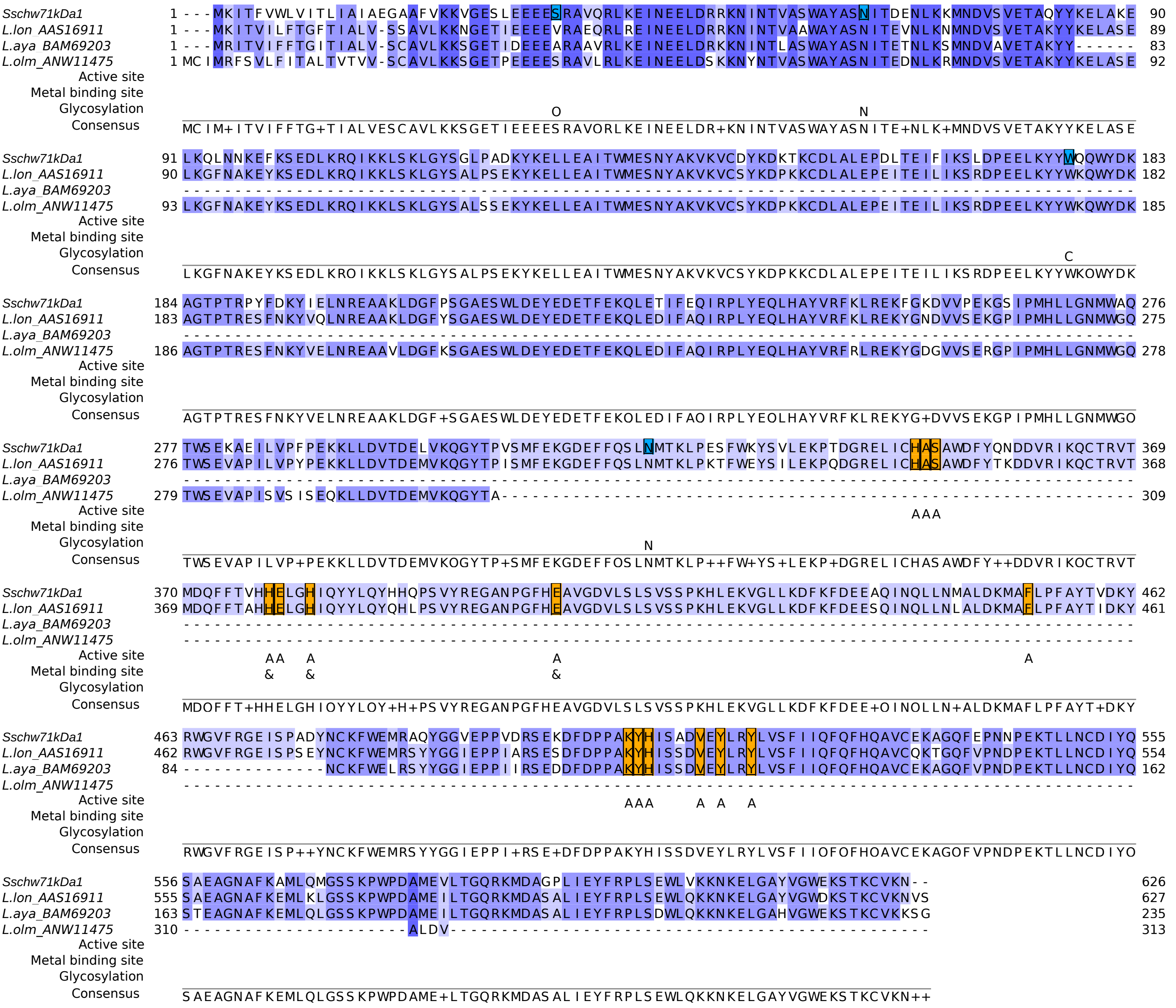

Multiple sequence alignment of S. schwetzi 71 kDa-like protein with other sand flies 71 kDa-like proteins. Name of sequence include sand fly species shortcut (L.lon – *L. longipalpis*, L.aya – *L. ayacuchensis*, L.olm – *L. olmeca*) and GenBank accession number or UniProtKB accession number. Sequence conservation is depicted by shading of purple color. Active sites of enzyme are highlighted in orange, putative glycosylation sites in Sschw71kDa1 sequence are highlighted in blue. Lines below the alignment indicates active site of enzyme by "A", metal binding site by "&", glycosylation by "N" for N-glycosylation, "O" for O-glycosylation, "C" for C-glycosylation and consensus sequence. Alignment was made by MAFFT with L-INS-i method and visualized in Jalview.
