# Supplementary material for: Sergentomyia schwetzi: Salivary gland transcriptome, proteome and enzymatic activities in two lineages adapted to different blood sources
Source: PLoS One. 2020 Mar 24;15(3):e0230537. doi: 10.1371/journal.pone.0230537 (PMC7092997; doi:10.1371/journal.pone.0230537)

S17 Fig. RNA-seq transcriptome analysis of *S. schwetzi* salivary glands

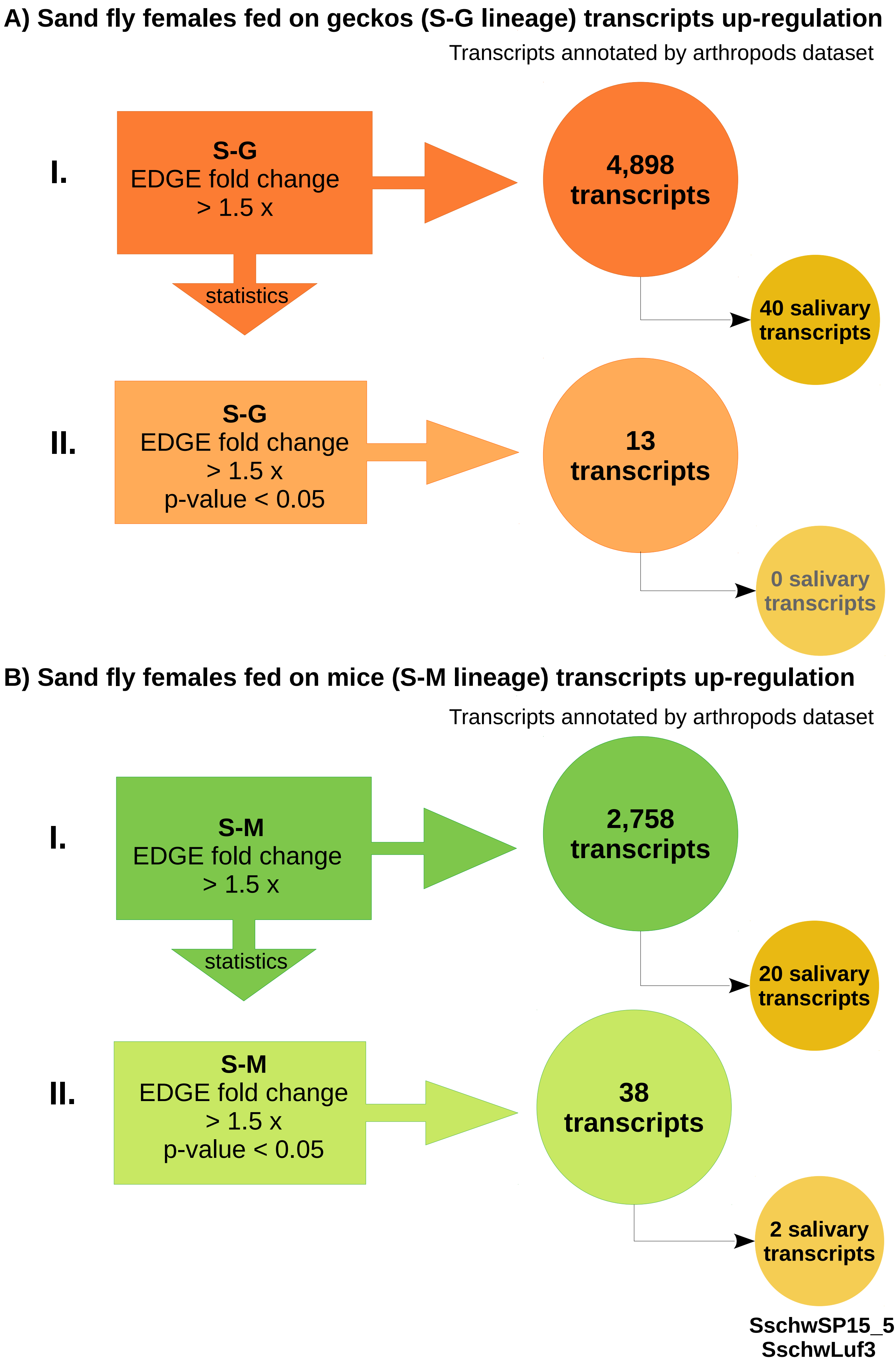

Supplement: S17 Fig — (PDF) [file pone.0230537.s017.pdf]
