# Supplementary material for: Sergentomyia schwetzi: Salivary gland transcriptome, proteome and enzymatic activities in two lineages adapted to different blood sources
Source: PLoS One. 2020 Mar 24;15(3):e0230537. doi: 10.1371/journal.pone.0230537 (PMC7092997; doi:10.1371/journal.pone.0230537)

S18 Fig. Proteome analysis of *S. schwetzi* salivary glands

A) Distribution of the proteins from whole proteome dataset

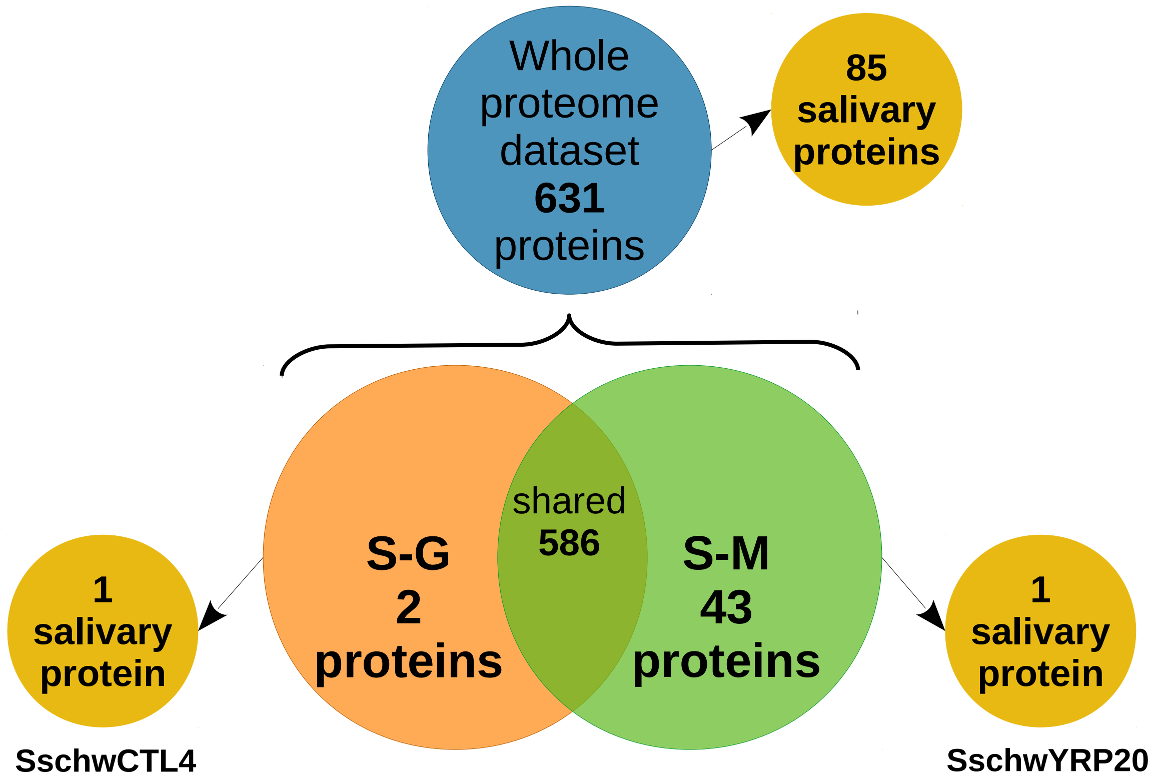

B) Distribution of the enriched proteins either in S-G or S-M sialome

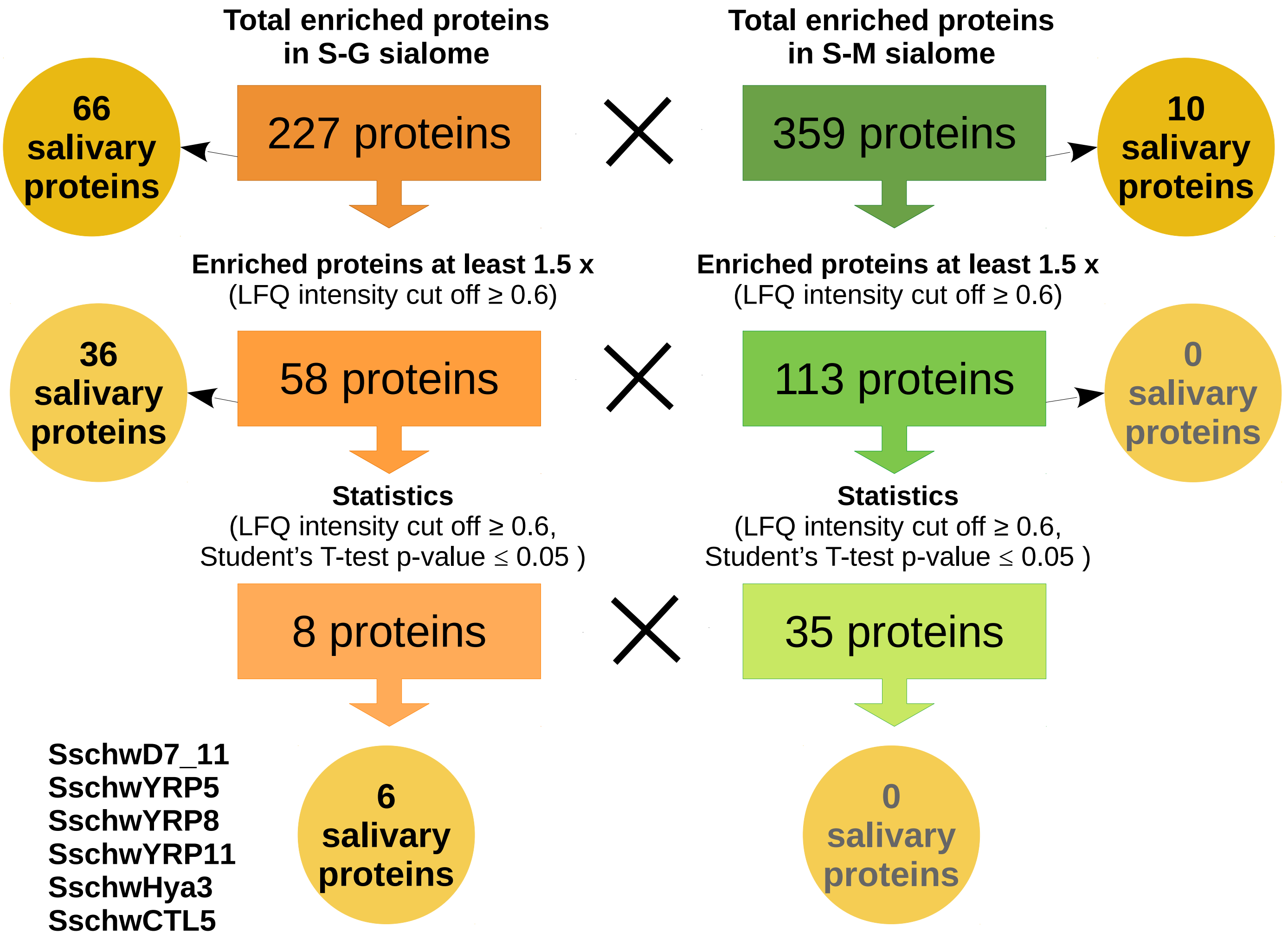

Supplement: S18 Fig — (PDF) [file pone.0230537.s018.pdf]
