# Supplementary material for: Sergentomyia schwetzi: Salivary gland transcriptome, proteome and enzymatic activities in two lineages adapted to different blood sources
Source: PLoS One. 2020 Mar 24;15(3):e0230537. doi: 10.1371/journal.pone.0230537 (PMC7092997; doi:10.1371/journal.pone.0230537)
Supplement: S19 Fig — Comparison of ATPase and ADPase activities and their pH optima in two S. schwetzi lineages maintained on different blood-meal sources, geckos (S-G) and mice (S-M). Results represent the mean of five independent measurements. (PDF) [file pone.0230537.s019.pdf]

S19 Fig. Comparison of ATPase and ADPase activities and their pH optima in two *S. schwetzi* lineages

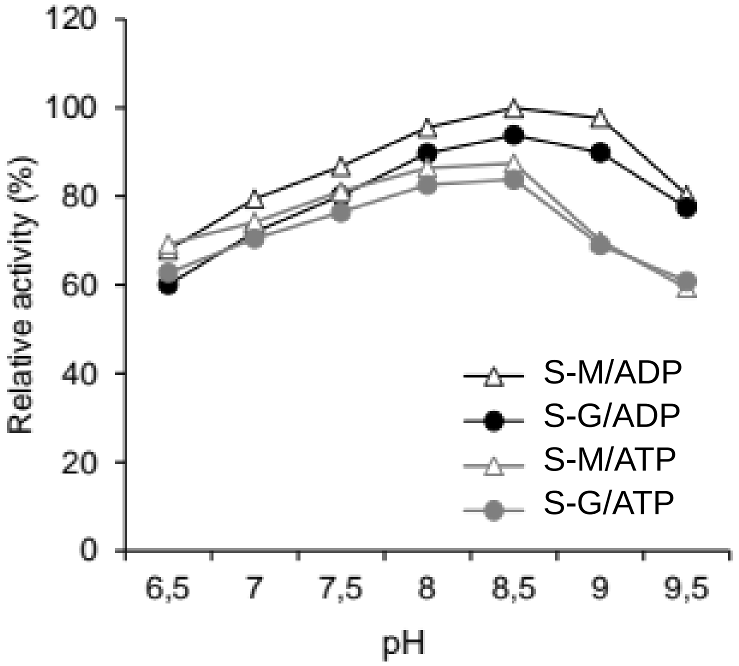

Comparison of ATPase and ADPase activities and their pH optima in two *S. schwetzi* lineages maintained on different blood-meal sources, geckos (S-G) and mice (S-M). Results represent the mean of five independent measurements.
